# Discovery of Staircase Chirality through the Design of Unnatural Amino Acid Derivatives

**DOI:** 10.34133/research.0550

**Published:** 2024-12-19

**Authors:** Anis U. Rahman, Yu Wang, Ting Xu, Kambham Devendra Reddy, Shengzhou Jin, Jasmine X. Yan, Qingkai Yuan, Daniel Unruh, Ruibin Liang, Guigen Li

**Affiliations:** ^1^ School of Chemistry and Chemical Engineering, Nanjing University, Nanjing 210093, China.; ^2^ Department of Chemistry and Biochemistry, Texas Tech University, Lubbock, TX 79409-1061, USA.; ^3^ Iowa Advanced Technology Laboratories, University of Iowa, Iowa City, IA 52242, USA.

## Abstract

Chirality has garnered significant attention in the scientific community since its discovery by Louis Pasteur over a century ago. It has been showing a profound impact on chemical, biomedical, and materials sciences. Significant progress has been made in controlling molecular chirality, as evidenced by the several Nobel Prizes in chemistry awarded in this area, particularly for advancements in the asymmetric catalytic synthesis of molecules with central and axial chirality. However, the exploration of new types of chirality has been largely stagnant for more than half a century, likely due to the complexity and challenges inherent in this field. In this work, we present the discovery of a novel type of chirality—staircase chirality as inspired by the design and synthesis of unnatural amino acid derivatives. The architecture of staircase chirality is characterized by 2 symmetrical phenyl rings anchored by a naphthyl pier, with the rings asymmetrically displaced due to the influence of chiral auxiliaries at their para positions. This unique staircase chiral framework has been thoroughly characterized using spectroscopic techniques, with its absolute configuration definitively confirmed by x-ray diffraction analysis. Remarkably, one of the staircase molecules exhibits 4 distinct types of chirality: central, orientational, turbo, and staircase chirality, a combination that has not been previously documented in the literature. Computational studies using density functional theory (DFT) calculations were conducted to analyze the relative energies of individual staircase isomers, and the results are in agreement with our experimental findings. We believe that this discovery will open up a new research frontier in asymmetric synthesis and catalysis, with the potential to make a substantial impact on the fields of chemistry, medicine, and materials science.

## Introduction

Molecular chirality has long been recognized as closely tied to the origin of life on Earth, as essential biomolecules in animals and plants—such as DNA, RNA, proteins, peptides, and carbohydrates—possess chiral structural elements vital to biological processes [1–4]. Chirality manifests in diverse forms, ranging from microscopic organisms like helical bacteria to macroscopic objects like seashells [5]. The fascination with chirality, in both scientific and public communities, has thus been well-founded [6,7]. Over the past century, research on chirality has transformed the fields of biology, medicine, chemistry, and materials science [8–15]. In chemical sciences, significant progress has been made in controlling molecular chirality [16–24] as evidenced by multiple Nobel Prize in chemistry awarded in recognition.

**Fig. 1.**
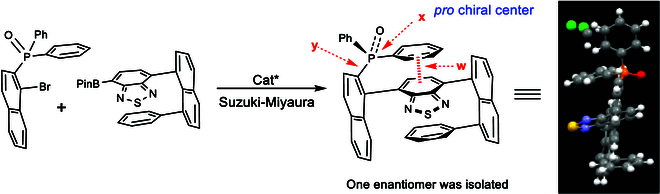
Turbo chirality in multilayer frameworks (x, y, and w show pro chiral center, orientational axis, and aromatic interaction, respectively).

Molecular chirality is generally classified into several categories. At the small-molecule level, it includes central chirality [25–35], axial chirality [36–46], spiral chirality [16,36], sandwich chirality (metallic [47,48] and organo [49–56]), turbo or propeller chirality[55], and orientational chirality [56–59]. At the macro and polymeric molecular level, it encompasses multilayer chirality (rigid helical [13,60] and flexible folding [61,62]), as well as topological and inherent chirality [63,64]. Among these categories, orientational chirality is the most recent addition, defined by the C(sp^2^)–C(sp^3^) or C(sp)–C(sp^3^) axis with remotely anchored blockers [56–59]. The orientational model exhibits 3 major energy barriers, contrasting with the 6 found in classic Felkin-Ahn or Cram models. This new form of chirality was revealed through our study of multilayer chiral frameworks via asymmetric catalytic C–C bond formation (Fig. 1) [49].

**Fig. 2.**
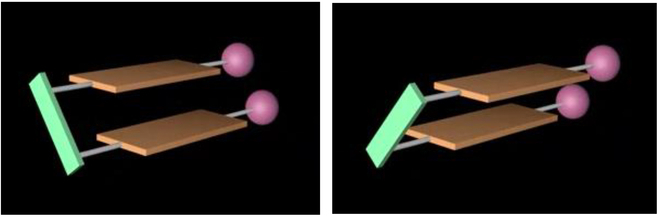
Logos representing the staircase chirality of 2 isomers.

X-ray diffraction of the multilayer framework displays orientational and turbo chiral patterns through 3 propellers (Fig. 2) [49]. Specifically, aromatic–aromatic interactions differentiate the 2 phenyl groups on the P=O moiety and produce a turbo chiral pattern, with 3 aromatic rings surrounding the P=O axis. The bridge between the 2 piers is a non-symmetrical benzo[c][1,2,5]thiadiazole ring, which also acts as a significant asymmetric factor. When we attempted to explore new chiral architectures by replacing the benzo[c][1,2,5]thiadiazole ring with a symmetric phenyl ring, the asymmetric catalytic C–C bond formation was unsuccessful, resulting in minimal product formation under various conditions. Shifting focus, we investigated simpler 2-layer frameworks attached to chiral amide auxiliaries. We discovered that 2 phenyl rings on the naphthyl pier could be arranged asymmetrically, leading to the identification of a new chirality pattern, which we are provisionally calling “staircase chirality”. This chirality is illustrated in Fig. 2, featuring 2 symmetrical layers that slip in opposite directions. Two functional groups, represented by 2 spheres, may be identical or different. In this report, we present our preliminary findings on this novel chirality element.

## Results and Discussion

**Fig. 3.**
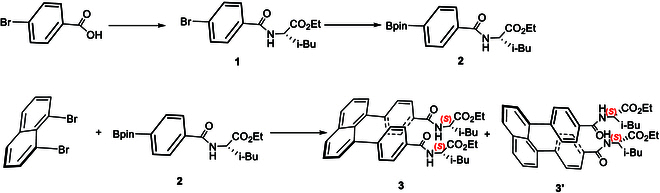
Asymmetric assembly of staircase amide.

In the initial phase, chiral amino acids were selected as the 2 auxiliaries at the para positions of the phenyl rings due to their potential for use in peptide drug design and synthesis [8,9]. Another key factor in this choice was the likelihood of obtaining high-quality crystals suitable for x-ray diffraction analysis, which is critical for this project as the slipped isomeric structures are challenging to distinguish by other analytical methods. To assemble the first framework (Fig. 3), (*S*)-ethyl *L*-leucinate hydrochloride was protected with 4-bromobenzoyl chloride, yielding the precursor ethyl (4-bromobenzoyl)-(*S*)-leucinate (**1**). This precursor was then converted to ethyl (4-(4,4,5,5-tetramethyl-1,3,2-dioxaborolan-2-yl)benzoyl)-*D*-leucinate (**2**) via a coupling reaction involving bis(pinacolato)diboron, using Pd(dppf)Cl₂ as a catalyst and potassium acetate. The resulting building block (**2**) was coupled with 1,8-dibromonaphthalene through a slightly modified Suzuki–Miyaura cross-coupling reaction, using Pd(PPh₃)₄ as a catalyst and K₂CO₃ as a base in a THF/H₂O (6:1, v/v) co-solvent system. The final product, ethyl (4-(8-(4-(((*R*)-1-ethoxy-4-methyl-1-oxopentan-2-yl)carbamoyl)phenyl)naphthalen-1-yl)benzoyl)-*L*-leucinate (**3**), was obtained with an overall chemical yield of 36% from the starting material (**1**).

**Fig. 4.**
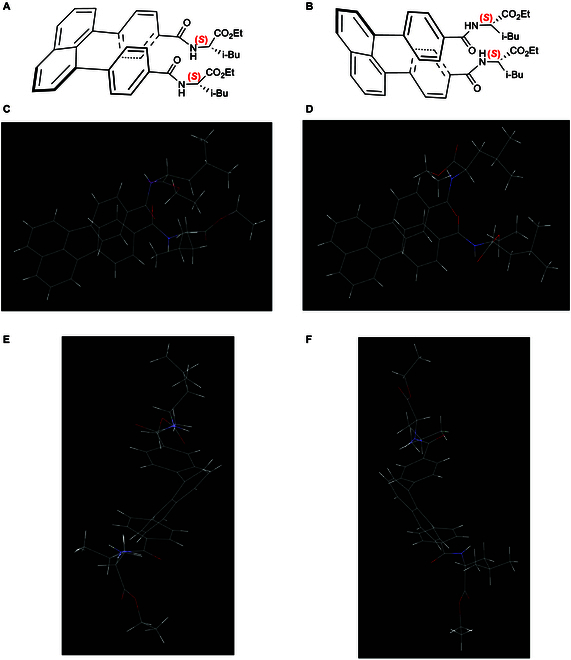
Two staircase isomers of target x and their x-ray diffraction analysis. (A and B) Chemical structures of two staircase isomers. (C and D) Views of their x-ray structures from top to bottom. (E and F) Views of their x-ray structures from side to side.

As anticipated, the product formed high-quality crystals, enabling x-ray diffraction analysis. As shown in Fig. 4, its x-ray structure reveals 2 co-existing diastereomers, each displaying 2 types of chirality: central chirality and staircase chirality. The 2 symmetrical phenyl rings exhibit asymmetric slippage; when the naphthyl anchor is positioned as shown in Fig. 4, the top phenyl ring slips either to the right (Fig. 4A) or to the left (Fig. 4B). Since there is no established nomenclature for this new form of chirality, we propose a provisional naming system as follows: (a) Observe the “stairs” from the naphthyl anchor to the other end of the phenyl ring, which is attached to the auxiliaries. (b) Imagine climbing the stairs from the bottom to the top. (c) The *P*-configuration is assigned to rightward climbing, while the *M*-configuration is assigned to leftward climbing. Based on this temporary system, isomers a and b would be classified as *P*- and *M*-configurations, respectively. Thus, the full names of these diastereomers are *P*- and *M*-diethyl 2,2′-((4,4′-(naphthalene-1,8-diyl)bis(benzoyl))bis(azanediyl))(2*S*,2′*S*)-bis(4-methylpentanoate), respectively.

Although the same chiral central auxiliaries are attached to the para-positions of the 2 phenyl rings, their functional group orientations differ noticeably. Additionally, the carbonyl groups on the 2 phenyl rings are arranged differently. In the *M*- and *P*-isomers, the C=O groups are oriented clockwise and counterclockwise, respectively, opposite to the rotation of their parent phenyl rings. These 2 factors likely contribute to the slipped staircase chirality observed. Interestingly, the 2 isobutyl groups in the *M-*isomer face the same direction, while those in the *P*-isomer face opposite directions, with a remote CH–CH₂ bond angle of approximately 85°. In both (*S*)-leucine residues of these 2 targets, a comparison of the 2 planar units of [C(O)NH] connected to the C(sp^3^) center of [CH(iBu)(CO₂Et)] shows that the *M*-isomer follows a Felkin-Ahn-type orientation. However, in the *P*-isomer, 2 distinct rotations occur between the [C(O)NH] and [CH(iBu)(CO₂Et)] units, where the C=O group is either aligned with or oriented away from the C*–H bond (where * indicates the chiral center). These conformational differences result in varying distances between the phenyl rings of the *M*- and *P*-diastereomers, measuring 4.041 and 4.192 Å, respectively, as determined from the centers of the phenyl rings.

**Fig. 5.**
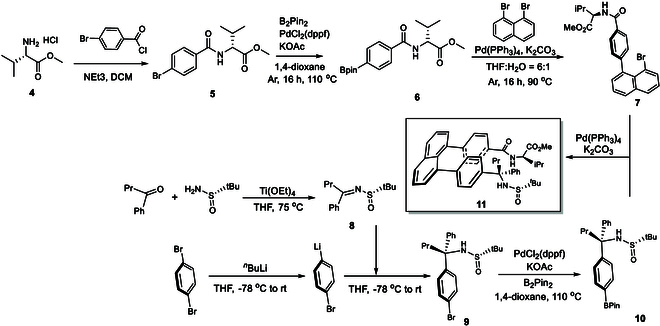
Asymmetric synthesis of target 11 by using (*R*)-valine/(*S*)-sulfinamide auxiliaries.

**Fig. 6.**
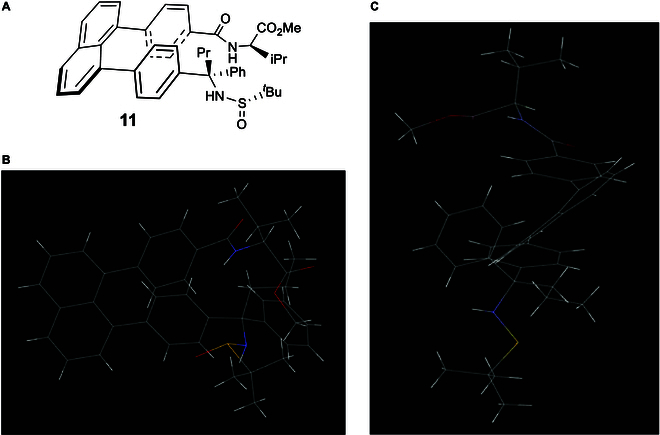
Staircase isomer 11 and its x-ray structural analysis. (A) Chemical structure of one staircase isomer. (B) View of its x-ray structure from top to bottom. (C) View of its x-ray structures from side to side.

Inspired by these initial results, we next explored the stereoselective formation of single staircase isomers, rather than a mixture of *M*- and *P*-diastereomers. We designed and synthesized a new staircase target containing (*R*)-valine amide and chiral sulfonimine-derived amine auxiliaries. The synthesis involved assembling 2 building blocks: methyl (4-(8-bromonaphthalen-1-yl)benzoyl)-(*R*)-valinate (**7**) and (*S*)-2-methyl-*N*-((*S*)-1-phenyl-1-(4-(4,4,5,5-tetramethyl-1,3,2-dioxaborolan-2-yl)phenyl)butyl)propane-2-sulfinamide (**10**) (Fig. 5). The first building block (**7**), methyl (4-(8-bromonaphthalen-1-yl)benzoyl)-(*R*)-valinate, was synthesized in 3 steps: amide formation, Pd-catalyzed C-Bpin coupling [65], and Suzuki–Miyaura C–C coupling [66,67]. The second building block (**10**), (*S*)-*N*-((*S*)-1-(4-bromophenyl)-1-phenylbutyl)-2-methylpropane-2-sulfinamide, was also obtained through a 3-step process: aryl lithium formation, dehydration, and asymmetric carbonyl addition, with the absolute configuration of the chiral C(sp^3^) center assigned by following a similar asymmetric induction method reported in the literature [68,69]. The final step involved a Suzuki–Miyaura cross-coupling reaction between the 2 building blocks, yielding methyl (4-(8-(4-((*S*)-1-(((*S*)-tert-butylsulfinyl)amino)-1-phenylbutyl)phenyl)naphthalen-1-yl)benzoyl)-(*R*)-valinate (**11**), which formed crystalline structures suitable for x-ray diffraction analysis (Fig. 6).

As shown in Fig. 6, the 2 chiral auxiliaries were able to stereoselectively control the chirality of the staircase target (**11**) in the *P-*configuration. Notably, unlike the 2 isomers in Fig. 4, where the [C(=O)NH] scaffolds lie in the same plane as the phenyl ring as expected, in this case, the scaffold is distorted by about 30° away from the phenyl ring. This distortion is caused by repulsion between the 2 terminal auxiliary groups. The chiral carbon center connected to the scaffold does not follow the typical Felkin-Ahn model arrangement; instead, the planar scaffold is aligned along the C–H bond of the C(sp^3^) center. The steric effects of the 2 auxiliaries push the 2 phenyl plates to slip relative to each other, resulting in staircase chirality. The distance between the 2 phenyl rings, measured from their centers, is 3.952 Å, which is shorter than the distances observed in the 2 isomers shown in Fig. 4.

At the same time, these effects force the second alkyl (*i*-Pr) group and the *N*-*t*-Bu-sulfinyl group to move away from each other; this belongs to orientational chirality as recently established by our lab [56–59]. Consequently, the present chiral target exhibits 3 types of chirality: central, orientational, and staircase chirality.

**Fig. 7.**
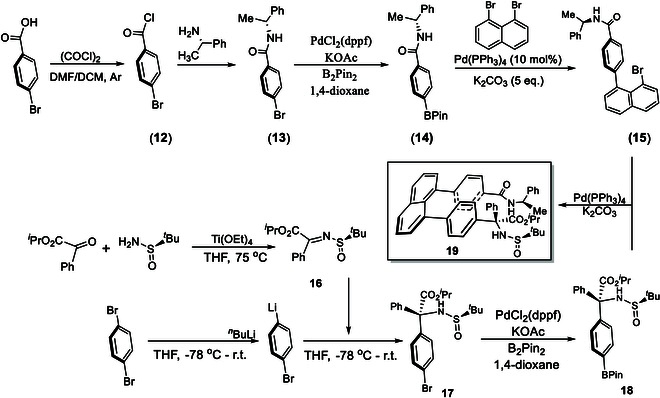
Asymmetric synthesis of target 19 by using (*R*)-4-*N*-(1-phenylethyl)amide/sulfinamide auxiliaries.

With this success on hand, we then turned our attention to the staircase target containing chiral tertiary amino acid and (*R*)- or (*S*)-1-phenylethan-1-amine auxiliaries; the latter auxiliary has been proven to be efficient on controlling orientational chirality [39–42]. The synthesis of the first building block (**15**) was started from commercially available 4-bromobenzoic acid, which was subjected to the treatment with oxalyl dichloride to give 4-bromobenzoyl chloride (**12**) (Fig. 7). This step was followed by nucleophilic reaction of (**12**) with (*R*)-1-phenylethan-1-amine to give (*R*)-4-bromo-*N*-(1-phenylethyl)benzamide (**13**) [57]. (*R*)-*N*-(1-phenylethyl)-4-(4,4,5,5-tetramethyl-1,3,2-dioxaborolan-2-yl)benzamide (**14**) was obtained by treating with B_2_Pin_2_ in the presence of PdCl_2_(dppf) as the catalyst and KOAc as an additive in 1,4-dioxane. The synthesis of the first building block (**15**), (*R*)-4-(8-bromonaphthalen-1-yl)-*N*-(1-phenylethyl)benzamide, was performed by conducting Suzuki–Miyaura cross-coupling. The synthesis of the second building block (**18**) was started from dehydration of isopropyl 2-oxo-2-phenylacetate with (*R*)-2-methylpropane-2-sulfinamide by using Ti(OEt)_4_ in dry THF at 75 °C to room temperature. 1,4-Dibromobenzene was converted into (4-bromophenyl)lithium precursor via the treatment with *n*-BuLi in THF, which was followed by the treatment with isopropyl (*S,E*)-2-((*tert*-butylsulfinyl)imino)-2-phenylacetate (**16**) to give isopropyl (*R*)-2-(4-bromophenyl)-2-(((*S*)-*tert*-butylsulfinyl)amino)-2-phenylacetate (**17**); it was then transformed into its BPin derivative, isopropyl (*R*)-2-(((*S*)-*tert*-butylsulfinyl)amino)-2-phenyl-2-(4-(4,4,5,5-tetramethyl-1,3,2-dioxaborolan-2-yl)phenyl)acetate (**18**) by reacting with B_2_Pin_2_ under the above catalytic system involving PdCl_2_(dppf) and KOAc. The assembly of 2 key building blocks **15** and **18** was furnished by performing Suzuki–Miyaura cross-coupling to afford target **19**, isopropyl (*R*)-2-(((*R*)-*tert*-butylsulfinyl)amino)-2-phenyl-2-(4-(8-(4-(((*R*)-1-phenylethyl)carbamoyl)phenyl)naphthalen-1-yl)phenyl)acetate.

Since (*R*)- or (*S*)-1-phenylethan-1-amine auxiliary was found to predominantly control previous orientational chirality as aforementioned, we also designed and synthesized isopropyl (*R*)-2-(((*R*)-tert-butylsulfinyl)amino)-2-phenyl-2-(4-(8-(4-(((*S*)-1-phenylethyl)carbamoyl)phenyl)naphthalen-1-yl)phenyl)acetate, i.e., to change (*R*)-4-*N*-(1-phenylethyl)amide to its (*S*)-counterpart while the sulfinamide and chiral carbon center are retained on the other side. The synthetic procedures for these 2 targets are the same as for those of target **20** in which only (*S*)-4-*N*-(1-phenylethyl)amide auxiliary was employed to replace its (*R*)-counterpart.

**Fig. 8.**
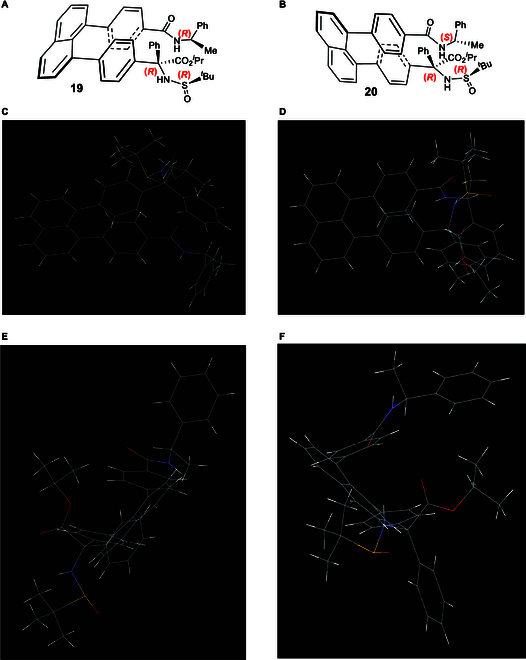
Chiral staircase targets 19 and 20 and their x-ray structural analysis. (A and B) Chemical structures of two staircase isomers. (C and D) Views of their x-ray structures from top to bottom. (E and F) Views of their x-ray structures from side to side.

Figure 8 displays absolute assignments based on x-ray diffraction analysis for staircase isomers **19** and **20**. Chiral tertiary amino acid auxiliaries of these 2 targets are the same involving (*R*)-carbon and (*R*)-sulfur chiral centers, but their 1-phenylethan-1-amine auxiliaries are different; i.e., targets **19** and **20** contain (*R*)- and (*S*)-1-phenylethan-1-amine-derived amides, respectively. (*R*)-1-Phenylethan-1-amine auxiliary was found to give *P*-staircase isomer, whereas its (*S*)-counterpart was found to give *M*-staircase isomer, which is similar to orientation chirality control in our previous work [39–42]. Obviously, the present staircase chirality is predominantly controlled by chiral amide auxiliary without being affected by chiral centers of C*(sp3) and S*(=O)*t*Bu of sulfinamide auxiliary.

In *P*-target **19**, the scaffold of [C(=O)NH] is placed in the same plane of phenyl ring as anticipated. However, in *M*-target **20**, this scaffold is distorted about 30° away from the phenyl ring due to the repulsion by 2 terminal auxiliary groups, which is like the situation observed in target **11** as shown in Fig. 6. In *P*-target **19**, the Felkin–Anh model fits both [C(=O)NH] scaffold and *N*-sulfinyl amino carbon centers, but in *M*-target **20**, the Felkin–Anh model only fits *N*-sulfinyl amino carbon center while the [C(=O)NH] scaffold is arranged nearly as long as the C–H bond of the chiral carbon. Obviously, the remote steric effects of these 2 auxiliaries are responsible for the present stereochemical arrangements. The distances between 2 centers of phenyl rings were measured to be 3.747 Å for *P*-target **19** and 3.473 Å for *M*-target **20**, respectively. Interestingly, the orientational chirality of 3 functional groups on tertiary carbon of *P* isomer **19** and *M*-isomer **20** is different, i.e., *N*-sulfinyl amino group is pushed away from (*R*)-amide auxiliary in the former, but the phenyl group is away from the (*S*)-amide auxiliary in the latter. Very interestingly, *M*-isomer **20** displays an obvious C(sp3)-centered turbo chirality by 3 propellers in *M,M,M-*configuration. However, there is no such structural arrangement in *P*-isomer **19**. Therefore, *M-*isomer **20** contains 4 types of chirality elements: central, orientational, turbo, and staircase chirality, which has not been documented in literature yet.

It should be noted that previous multilayer or organo sandwich frameworks contain non-symmetrical aromatic ring. However, the present staircase structures require the rings on 1,8-positions of naphthalene to be symmetrical. The current approach using staircase chirality will be applied to the design of unnatural amino acids for research on peptides and peptidomimetics, specifically by synthesizing individual *M*- and *P*-isomers. This approach could yield preferred conformations that better target macro biological molecules, such as receptors on cell surfaces, leading to peptide drug candidates with enhanced biomedical activity and fewer side and unwanted effects. In parallel, circularly polarized luminescent multilayer polymers and materials based on staircase chirality units offer promising research avenues through interdisciplinary collaboration. Adjusting distances between aromatic rings and functional groups on phenyl and naphthyl rings is expected to influence absorption and emission wavelengths of multilayer materials. Preliminary results in our lab indicate that multilayer polymers and their oligomers exhibit fluorescence in both solution and solid forms, demonstrating the potential of staircase chirality for practical applications.

### Computational Study

We performed quantum mechanical calculations using density functional theory (DFT) to evaluate the energies of a single molecule that is a derivative of **20** in the vacuum, together with its stereoisomers with different types of center and staircase chirality. It is observed that in the crystal structure, the -NH moiety on the amide group is involved in the intermolecular hydrogen bonds across neighboring cells, which is absent in the calculations performed here for an isolated molecule in the vacuum. For this reason, the -NH moiety on the amide group was replaced with the -NCH_3_ moiety to simplify the energy comparison among different stereoisomers. All calculations in this study were performed for this derivative.

**Fig. 9.**
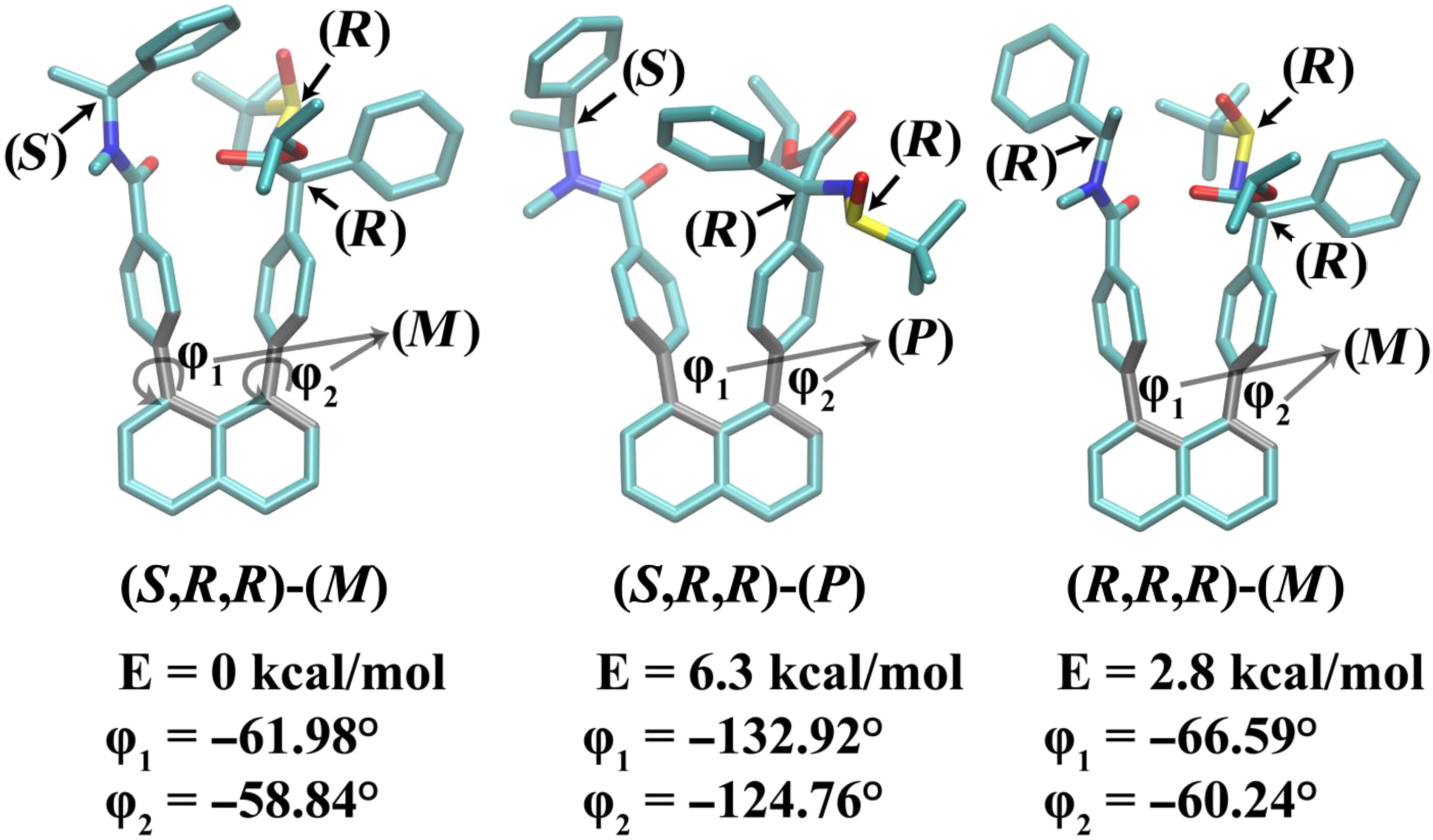
DFT optimized structures of different stereoisomers and their relative energies for a derivative of molecule 20 (see the main text). The 2 key torsions that characterize the staircase chirality are labeled as φ_1_ and φ_2_, and the atoms defining the 2 torsions are highlighted in gray. The chiral configuration, energy, and the values of the 2 torsions are summarized below each structure. “(*M*)” indicates *M*-staircase isomer, and “(*P*)” indicates *P*-staircase isomer. Black arrows and text labels indicate the chiral configuration of each center in each structure.

The structure of the (*S,R,R*)-(*M*) isomer (see Fig. 9 for definition) was constructed using the corresponding crystal structure of molecule **20** by replacing the NH moiety with the -NCH_3_ moiety. The structure of the (*R,R,R*)-(*M*) isomer was constructed from the (*S,R,R*)-(*M*) isomer by inverting the chirality of the carbon atom connected to the NCH_3_ moiety. The structure of the (*S,R,R*)-(*P*) isomer was constructed from the crystal structure of molecule **19** (the (*R,R,R*)-(*P*) isomer) by first replacing the -NH moiety with the -NCH_3_ moiety followed by inverting the chirality of the carbon atom connected to the -NCH_3_ moiety.

Following the generation of the initial structures of different isomers, their geometries were optimized using DFT with the B3LYP functional [70–73], the def2-SVP basis set [74], and the D3 dispersion correction [75] (B3LYP-D3/def2-SVP). At the optimized geometries, the energies were re-evaluated using the def2-TZVP basis set plus D3 dispersion correction (B3LYP-D3/def2-TZVP). The normal mode analysis and zero-point energy (ZPE) corrections were performed on these minima at the B3LYP-D3/def2-SVP level of theory. The final energy reported for each optimized structure was the B3LYP-D3/def2-TZVP energy plus the ZPE correction.

All geometry optimizations were performed with the DL-FIND [76] plugin in the ChemShell [77] software package. All DFT calculations were performed with the TURBOMOLE [78] software package.

Our computational results revealed that by changing from the (*S*,*R*,*R*)-(*M*) isomer to the (*R,R,R*)-(*M*) isomer, the energy increases by 2.8 kcal/mol (Fig. 9). This result suggests that the staircase chirality influences the relative thermal stability of the chirality of the carbon atom adjacent to the -NCH_3_ group. Additionally, changing from the (*S,R,R*)-(*M*) isomer to the (*S,R,R*)-(*P*) isomer features the large displacements of the 2 torsions connecting the naphthalene ring and the 2 benzyl rings (φ_1_, φ_2_, Fig. 9), and an energy increase of 6.3 kcal/mol (Fig. 9). This result suggests that the chirality of the carbon atom adjacent to the -NCH_3_ group is correlated with the thermal stability of the staircase chirality: when this carbon atom adopts the S chirality, the *M*-staircase isomer is more stable. Thus, the calculations are consistent with the crystal structures of the original compound with the amide group and support the experimentally observed coupling between the staircase chirality and the center chirality.

## Conclusion

We have discovered a novel chiral element—staircase chirality. Individual staircase isomers were selectively synthesized by simultaneously using 2 chiral auxiliaries: an amide and a sulfinamide. This chiral framework features 2 symmetrical phenyl rings bridged by a naphthyl core, with these phenyl rings asymmetrically displaced due to the chiral auxiliaries at their para positions. The resulting staircase chiral targets were fully characterized through spectroscopy, with their absolute configurations unambiguously confirmed by x-ray diffraction analysis. A nomenclature system was proposed for the staircase isomers, and one isomer was found to exhibit 4 distinct types of chirality elements: central, orientational, turbo, and staircase chirality—an unprecedented combination. Geometry optimizations using DFT were performed to assess the relative energies of the individual staircase isomers, aligning with our experiments. The present 2-layer unit will be utilized to build higher staircase oligomers and polymers. This discovery is expected to pave the way for new research in asymmetric synthesis and catalysis, with broad implications for the fields of chemistry, medicinal chemistry, and materials sciences.

## Materials and Methods

Unless otherwise stated, all reactions were magnetically stirred and conducted in oven-dried glassware in anhydrous solvents under Ar, applying standard Schlenk techniques. Solvents and liquid reagents, as well as solutions of solid or liquid reagents were added via syringes, stainless steel, or polyethylene cannulas through rubber septa or through a weak Ar counter-flow. Solvents were removed under reduced pressure at 40 to 65 °C using a rotavapor. All given yields are isolated yields of chromatographic and nuclear magnetic resonance (NMR) spectroscopic materials. All commercially available chemicals were used as received without further purification.

^1^H and ^13^C NMR spectra were recorded in CDCl_3_ on 400-MHz instruments with TMS as internal standard. For referencing of the ^1^H NMR spectra, the residual solvent signal (δ = 7.26 parts per million [ppm] for CDCl_3_) was used. In the case of the ^13^C NMR spectra, the signal of solvent (δ = 77.0 ppm for CDCl_3_) was used. Chemical shifts (δ) were reported in ppm with respect to TMS. Data are represented as follows: chemical shift, multiplicity (s = singlet, d = doublet, t = triplet, m = multiplet), coupling constant (*J*, Hz), and integration. Optical rotations were measured with a Rudolph Research Analytical APIV/2W Polarimeter at the indicated temperature with a sodium lamp. Measurements were performed in a 2-mL vessel with the concentration unit of g/100 mL in the corresponding solvents.

## Data Availability

All data are available in the manuscript or Supplementary Materials.
