# Supplementary material for: Discovery of Staircase Chirality through the Design of Unnatural Amino Acid Derivatives
Source: Research (Wash D C). 2024 Dec 19;7:0550. doi: 10.34133/research.0550 (PMC11658802; doi:10.34133/research.0550)
Supplement: Supplementary 1 — Figs. S1 to S10 [file research.0550.f1.pdf]

Discovery of Staircase Chirality through the Design of Unnatural Amino Acid Derivatives

Anis U. Rahman,[a,†] Yu Wang,[a,†] Ting Xu,[a] Kambham Devendra Reddy,[b] Shengzhou Jin,[a] Xianghua Yan,[b] Qingkai Yuan,[b] Daniel Unruh[c] Ruibin Liang,[*,b] and Guigen Li[*,a,b]

[a] *School of Chemistry and Chemical Engineering, Nanjing University, Nanjing, 210093, China.*
[b] *Department of Chemistry and Biochemistry, Texas Tech University, Lubbock, Texas 79409-1061, USA.*
[c] *Iowa Advanced Technology Laboratories, University of Iowa, 205 N. Madison Street, IA 52242, USA.*

[**]Correspondence should be addressed to Ruibin Liang and Guigen Li: rliang@ttu.edu *(RL)* guigenli@nju.edu.cn, guigen.li@ttu.edu *(GL)*

[†]These authors contributed equally to this work.

Supporting Information

Contents

1. General information S1
2. General Procedure A for the Synthesis of substrate **2** S1
3. General Procedure B for the Synthesis of substrate **3** S2
4. General Procedure C for the Synthesis of substrate **7** S3
5. General Procedure D for the Synthesis of substrate **10** S4
6. General Procedure E for the Synthesis of substrate **11** S5
7. General Procedure F for the Synthesis of substrate **19** and **20** S7
8. References S9
9. Copies of ^{1}H and ^{13}C spectra of compounds S10
10. X-ray Diffraction Data S10

1. General information

For general Information, all melting points are uncorrected. The NMR spectra were recorded in $CDCl_3$ on a 400 MHz instrument with TMS as the internal standard. Chemical shifts (δ) are reported in ppm with respect to TMS. Data are represented as follows: chemical shift, multiplicity (s = singlet, d = doublet, t = triplet, m = multiplet), coupling constant (J, Hz), and integration. High-resolution mass spectrometry (HRMS) analyses were carried out using a time-of-flight mass spectrometry (TOF-MS) instrument with an electrospray ionization (ESI) source. X-ray crystallographic analysis was performed with a SMART CCD and a P4 diffractometer. All commercially sourced starting materials were used without further purification.

2. General Procedure A for the Synthesis of substrate 2

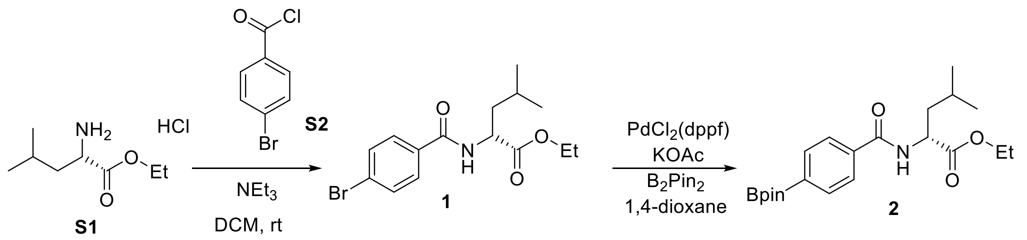

The substrates **2** was prepared according to the reported procedures and related literature.[1,2] To a solution of ethyl *L*-leucinate hydrochloride **S1** (15 mmol, 2.9353 g) and 4-bromobenzoyl chloride **S2** (1.1 equiv, 16.5 mmol, 3.6211 g) in dry DCM (50 mL) was added Et_3N (45 mmol, 3 equiv, 4.5535 g) at room temperature and stirred for 2 h. Wash the mixture with aq. HCl (1 M, 30 mL), saturated aq. $NaHCO_3$ (30 mL) successively. The combined organic layers were dried by $MgSO_4$ and concentrated the resulting mixture under reduced pressure. Finally, the **1** (92%, 4.7228 g) was obtained by recrystallizing the crude product.

The mixture of **1** (3.4223 g, 10 mmol), bis(pinacolato)diboron (3.0473 g, 12 mmol), potassium acetate (1.9628 g, 20 mmol), Pd(dppf)Cl_2 (0.7312 g, 1 mmol) was protected with Ar, and then the 80 mL dry 1,4-dioxane was added. The mixture was heated at 110 °C (oil bath) for 20 h. After cooling down the mixture was filtered through a pad of celite. Removal of solvent under reduced pressure afforded a residue which is purified by chromatography on silica gel (PE: EA= 4:1) to afford the white product **2** (50%, 1.3117 g).

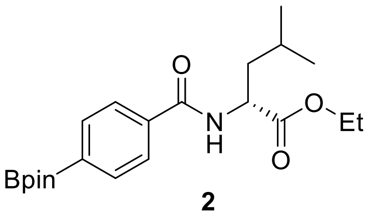

2

ethyl (4-(4,4,5,5-tetramethyl-1,3,2-dioxaborolan-2-yl)benzoyl)-*D*-leucinate 2: Purified by using a flash column chromatography (PE/EA = 4/1); isolated yield = 50%, 1.3117 g; white solid. ^{1}H NMR (400 MHz, $CDCl_3$) δ 7.88 (d, J = 8.2 Hz, 2H), 7.79 (d, J = 8.2 Hz, 2H), 6.63 (d, J = 8.2 Hz, 1H), 4.89 – 4.82 (m, 1H), 4.24 (q, J = 7.1 Hz, 2H), 1.79 – 1.65 (m, 3H), 1.37 (s, 12H), 1.31 (t, J = 7.1 Hz, 3H), 1.03 – 0.96 (m, 7H) ppm. ^{13}C NMR (101 MHz, $CDCl_3$) δ 173.2, 167.0, 136.3, 134.9, 126.1, 84.1, 61.4, 51.3, 42.0, 25.0, 24.9, 24.8, 22.8, 22.2, 14.1 ppm.

3. General Procedure B for the Synthesis of substrate 3

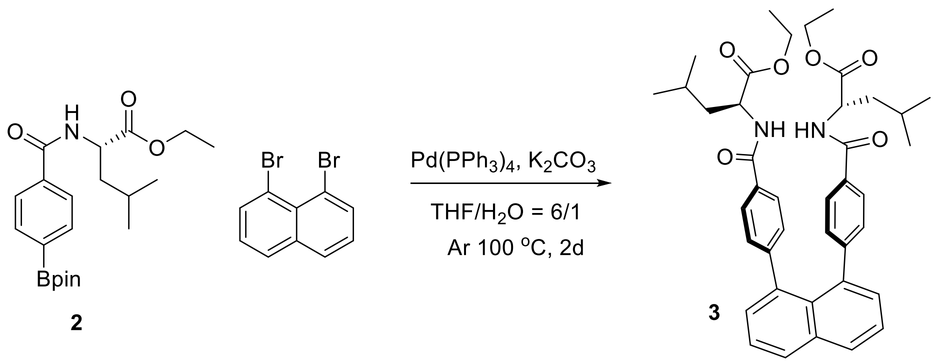

Schlenk tube was added 1,8-dibromonaphtalene (28.6 mg, 0.1 mmol), **2** (78.7 mg, 0.3 mmol), K_2CO_3 (82.9 mg, 0.6 mmol), and $Pd(PPh_3)_4$ (23.1 mg, 0.02 mmol). The THF (3 mL) and water (0.5 mL) were injected under Argon. The mixture was heated in an oil bath at 100 °C and stirred for 2 d. After cooling to room temperature, the liquid was separated with EA and water. The organic phase was taken and purified after concentration on silica gel (PE : EA = 3 : 1) to get the desired product **3**.

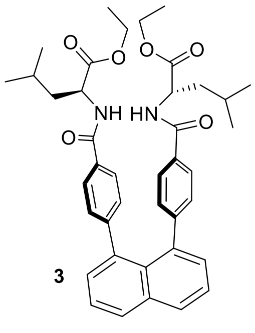

diethyl 2,2'-((4,4'-(naphthalene-1,8-diyl)bis(benzoyl))bis(azanediyl))(2*S*,2'*S*)-bis(4-methylpentanoate) 3: Purified by using a flash column chromatography (PE/EA = 3/1); isolated yield = 81%, 52.7 mg; white solid. $[\alpha]_D^{25}$ = 7.4 (c = 2.7, CH_2Cl_2). ^{1}H NMR (400 MHz, $CDCl_3$) δ 7.96 (dd, J = 8.2, 1.3 Hz, 2H), 7.54 (dd, J = 8.2, 7.0 Hz,

2H), 7.35 (dd, J = 7.0, 1.3 Hz, 2H), 7.30–7.23 (m, 4H), 6.93 (d, J = 8.1 Hz, 4H), 6.76 (d, J = 8.1 Hz, 2H), 4.70 (q, J = 7.3 Hz, 2H), 4.26–4.18 (m, 4H), 1.78–1.75 (m, 4H), 1.30 (t, J = 7.1 Hz, 6H), 1.04–0.98 (m, 12H) ppm. ^{13}C NMR (101 MHz, $CDCl_3$) δ 173.0, 168.3, 146.1, 139.2, 135.1, 132.8, 130.5, 123.0, 129.8, 129.4, 129.0, 126.0, 125.6, 125.1, 74.9, 61.1, 51.5, 41.3, 25.0, 24.8, 22.7, 22.2, 14.1 ppm. HRMS (ESI): *m/z* calcd. for $C_{40}H_{46}N_2O_6$ $[M + Na]^+$ 673.3249, found673.3232.

4. General Procedure C for the Synthesis of substrate 7

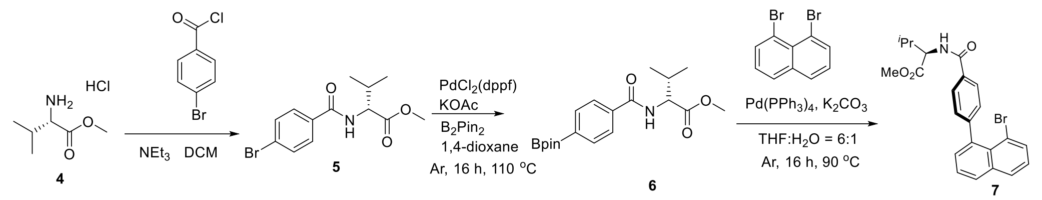

The substrates **7** was prepared according to the reported procedures and related literature.[3] To a solution of ethyl *L*-valine methyl ester hydrochloride **4** (15 mmol, 2.5144 g) and 4-bromobenzoyl chloride (1.1 equiv, 16.5 mmol, 3.6211 g) in dry DCM (50 mL) was added Et_3N (45 mmol, 3 equiv, 4.5535 g) at room temperature and stirred for 2 h. Wash the mixture with aq. HCl (1 M, 30 mL), saturated aq. $NaHCO_3$ (30 mL) successively. The combined organic layers were dried by $MgSO_4$ and concentrated the resulting mixture under reduced pressure. Finally, the **5** (89%, 4.1943 g) was obtained by recrystallizing the crude product.

The mixture of **5** (3.1418 g, 10 mmol), bis(pinacolato)diboron (3.0473 g, 12 mmol), potassium acetate (1.9628 g, 20 mmol), Pd(dppf)Cl_2 (0.7312 g, 1 mmol) was protected with Ar, and then the 80 mL dry 1,4-dioxane was added. The mixture was heated at 110 °C (oil bath) for 20 h. After cooling down the mixture was filtered through a pad of celite. Removal of solvent under reduced pressure afforded a residue which is purified by chromatography on silica gel (PE: EA= 4:1) to afford the white product **6** (52%, 1.2183 g).

Place 1,8-dibromonaphtalene (0.2860 g, 1 mmol), **6** (0.2343 g, 1 mmol), K_2CO_3 (0.4146 g, 3 mmol) and $Pd(PPh_3)_4$ (0.1156 g, 0.1 mmol) successively to a flask under an argon atmosphere. Add THF (12 mL) and water (2 mL). Heat the mixture in an oil bath at 90 °C. Stir the mixture for 16 h. Cooled the mixture to room temperature and the liquid was separated with EA and water. The organic phase was taken and purified after concentration on silica gel (PE : EA = 5 : 1) to get the desired product **7**.

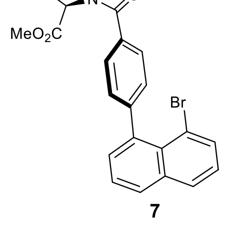

7

methyl (4-(8-bromonaphthalen-1-yl)benzoyl)-*L*-valinate 7: Purified by using a flash column chromatography (PE/EA = 5/1); isolated yield = 70%, 308.2 mg; white solid. ^{1}H NMR (400 MHz, $CDCl_3$) δ 7.92–7.88 (m, 2H), 7.87–7.82 (m, 2H), 7.79 (dd, J = 7.4, 1.3 Hz, 1H), 7.51 (dd, J = 8.2, 7.1 Hz, 1H), 7.45–7.41 (m, 2H), 7.39 (dd, J = 7.1, 1.4 Hz, 1H), 7.31 (t, J = 7.8 Hz, 1H), 6.72 (d, J = 8.6 Hz, 1H), 4.84 (dd, J = 8.6, 4.9 Hz, 1H), 3.80 (s, 3H), 2.36–2.27 (m, 1H), 1.04 (dd, J = 9.2, 6.9 Hz, 6H) ppm. ^{13}C NMR (101 MHz, $CDCl_3$) δ 172.7, 167.2, 146.7, 139.3, 136.1, 133.9, 132.6, 131.1, 130.4, 129.5, 129.4, 128.9, 126.3, 126.2, 126.1, 125.3, 112.0, 57.5, 52.2, 31.7, 19.0, 18.1 ppm.

5. General Procedure D for the Synthesis of substrate 10

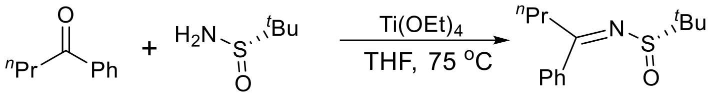

8

The substrates **10** was prepared according to the reported procedures and related literature.[4] To a solution of (*S*)-(+)-2-methyl-2-propane sulfinamide (0.6 g, 5 mmol, 1 eq) and 1-phenylbutan-1-one (0.8892 g, 6 mmol, 1.2 eq) in anhydrous THF (30 mL) at room temperature was added $Ti(OEt)_4$ (6 mL, 15 mmol, 3 eq). The mixture was heated at 75 °C for 24 h. After cooling to room temperature, the mixture was poured into 30 mL of brine under vigourous stirring. The resulting suspension was filtered through a pad of Celite and the solid was washed with EtOAc (3 x 20 mL). The filtrate was washed with brine (30 mL), dried (Na_2SO_4), and concentrated in vacuo. The residue was chromatographed on silica by eluting with PE/EA (20:1 to 10:1) to get the pure product **8** (1.1313 g, 90% yield).

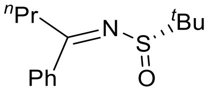

8

(*S*, *Z*)-2-methyl-N-(1-phenylbutylidene)propane-2-sulfinamide 8: Purified by using a flash column chromatography (PE/EA = 20/1); isolated yield = 90%, 1.1313 g; yellow oil. ^{1}H NMR (400 MHz, $CDCl_3$) δ 7.82 (s, 2H), 7.47–7.39 (m, 3H), 3.23–3.14 (m, 2H), 1.78–1.65 (m, 2H), 1.31 (s, 9H), 1.02 (t, J = 7.2 Hz, 3H) ppm. ^{13}C NMR (101 MHz, $CDCl_3$) δ 180.1, 138.0, 131.4, 128.5, 127.4, 57.4, 34.4, 22.6, 22.2, 14.2 ppm.

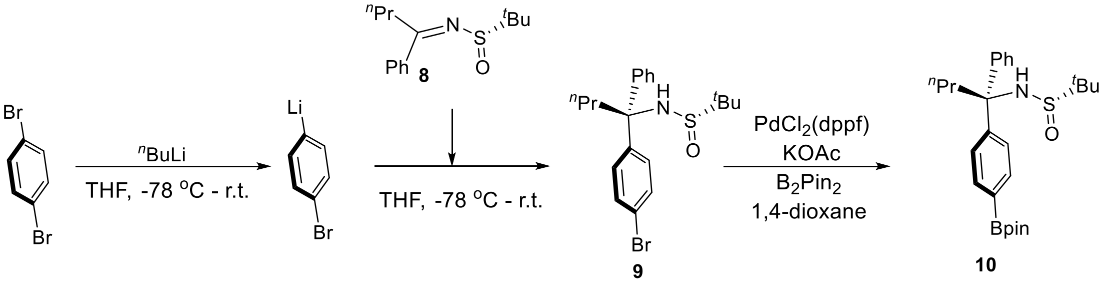

To a solution of 1,4-Dibromobenzene (2.3590 g, 10 mmol) in dry THF (30 mL) at -78 °C was added *n*-butyllithium (6.25 mL, 1.6 M in hexanes). The resulting solution

was stirred for 2 hours, a solution of **8** (2.5139 g, 10 mmol, 1 equiv) in dry THF (30 mL) was slowly added via syringe. Stirring was continued at –78 °C for 2 hours, and then heat the solution to room temperature for 14 hours. Subsequently, the saturated aqueous NH_4Cl was added to the solution, the organic phase was extracted with EA and dried over anhydrous $MgSO_4$. Chromatography (PE/EA= 10/1) afforded the diastereomerically pure propargyl **9**.

The mixture of **9** (1.5520 g, 3.8 mmol, 1 equiv), bis(pinacolato)diboron (2.2918 g, 9.5 mmol, 2.5 equiv), potassium acetate (1.1187 g, 11.4 mol, 3 equiv), Pd(dppf)Cl_2 (0.2778 g, 0.38 mmol, 0.1 equiv) was protected with Ar, and then the 60 mL dry 1,4-dioxane was added. The mixture was heated at 110 °C (oil bath) for 20 h. After cooling down the mixture was filtered through a pad of celite. Removal of solvent under reduced pressure afforded a residue which is purified by chromatography on silica gel (PE: EA= 5:1) to afford the coupling product **10**.

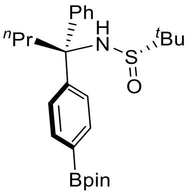

10

(*S*)-2-methyl-*N*-((*S*)-1-phenyl-1-(4-(4,4,5,5-tetramethyl-1,3,2-dioxaborolan-2-yl)phenyl)butyl)propane-2-sulfinamide 10: Purified by using a flash column chromatography (PE/EA = 5/1); isolated yield = 60%, 1.0384 g; pale yellow oil. ^{1}H NMR (400 MHz, $CDCl_3$) δ 7.77 (d, J = 8.0 Hz, 1H), 7.39–7.33 (m, 3H), 7.31–7.26 (m, 5H), 4.09 (s, 1H), 2.68–2.60 (m, 1H), 2.55–2.47 (m, 1H), 1.34 (s, 9H), 1.32–1.27 (m, 2H), 1.24 (s, 12H), 0.94 (t, J = 5.6 Hz, 3H) ppm. ^{13}C NMR (101 MHz, $CDCl_3$) δ 145.3, 143.2, 133.8, 129.2, 128.8, 128.2, 127.5, 127.0, 83.8, 70.6, 56.6, 33.5, 24.8, 22.8, 18.6, 18.3 ppm.

6. General Procedure E for the Synthesis of substrate 11

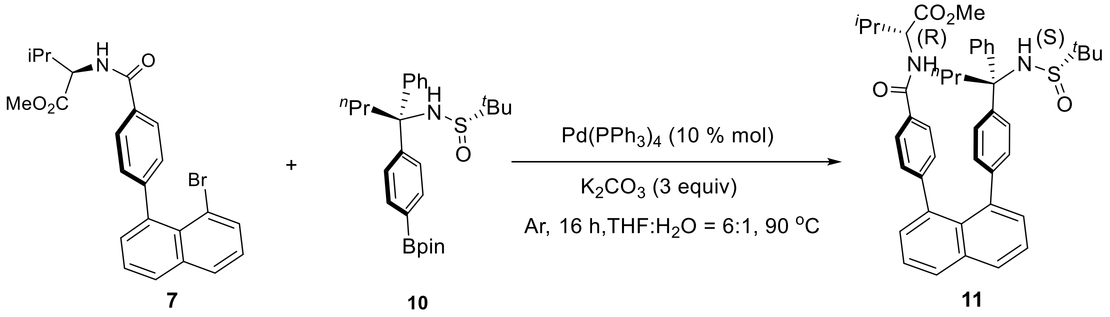

7 **10** **11**

Place **7** (88.1 mg, 0.2 mmol), **10** (65.7 mg, 0.2 mmol), K_2CO_3 (82.9 mg, 0.6 mmol) and Pd(PPh_3)$_4$ (23.1 mg, 0.02 mmol) successively to a flask under an argon atmosphere. Add THF (3 mL) and water (0.5 mL). Heat the mixture in an oil bath at 90 °C. Stir the mixture for 16 h. Cooled the mixture to room temperature and the liquid was separated with EA and water. The organic phase was taken and purified after concentration on

silica gel (PE : EA = 3 : 1) to get the desired product **11**.

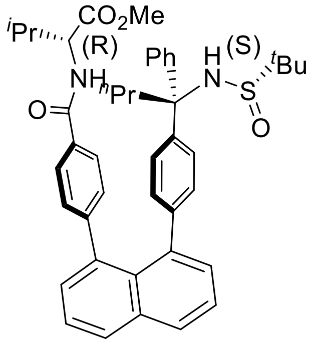

11

methyl (4-(8-(4-((*S*)-1-(((*S*)-tert-butylsulfinyl)amino)-1-phenylbutyl)phenyl)naphthalen-1-yl)benzoyl)-*D*-valinate 11: Purified by using a flash column chromatography (PE/EA = 3/1); isolated yield = 53%, 36.5 mg; white solid. $[\alpha]_D^{25}$ = 2.3 (c = 0.6, CH_2Cl_2). ^{1}H NMR (400 MHz, Chloroform-*d*) δ 7.96 (td, J = 8.3, 1.4 Hz, 2H), 7.59–7.55 (m, 2H), 7.55–7.51 (m, 2H), 7.46 (dd, J = 7.1, 1.4 Hz, 1H), 7.37–7.33 (m, 3H), 7.27–7.23 (m, 1H), 7.20–7.12 (m, 5H), 7.05 (s, 2H), 6.96 (d, J = 8.0 Hz, 1H), 6.77 (d, J = 7.7 Hz, 1H), 6.62 (d, J = 8.5 Hz, 1H), 4.77 (dd, J = 8.5, 5.0 Hz, 1H), 3.92 (s, 1H), 3.77 (s, 3H), 2.35 (dd, J = 10.4, 4.4 Hz, 2H), 2.28–2.20 (m, 1H), 1.21 (s, 9H), 0.98 (t, J = 7.0 Hz, 6H), 0.91–0.86 (m, 4H) ppm. ^{13}C NMR (101 MHz, $CDCl_3$) δ ^{13}C NMR (101 MHz, $CDCl_3$) δ 172.7, 166.5, 147.3, 144.9, 144.0, 141.7, 139.8, 139.3, 135.5, 131.4, 131.2, 131.1, 129.9, 129.1, 129.0, 128.4, 128.1, 127.9, 127.0, 126.0, 125.4, 125.1, 66.4, 57.5, 56.3, 52.1, 42.3, 31.7, 24.8, 22.8, 18.8, 18.2, 17.2, 14.2 ppm. HRMS (ESI): *m/z* calcd. for $C_{43}H_{48}N_2O_4S$ $[M + Na]^+$ 711.3227, found711.3209.

7. **General Procedure F for the Synthesis of substrate 19 and 20**

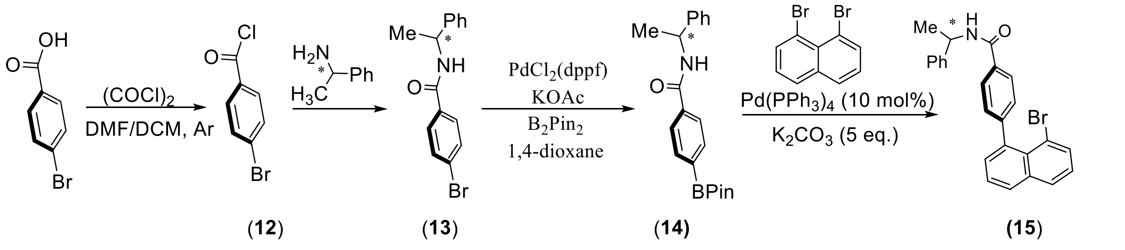

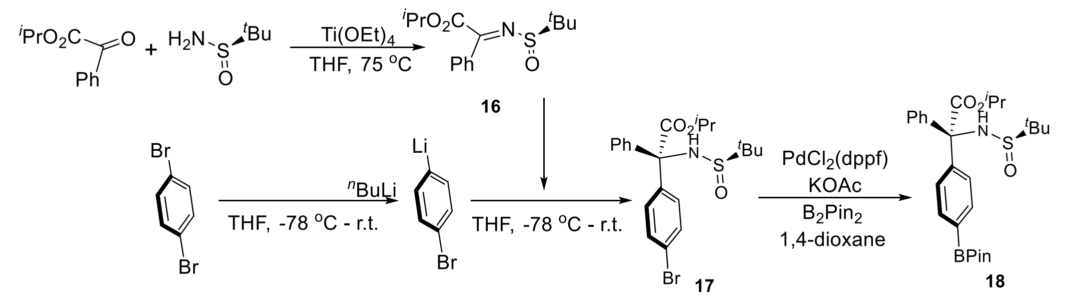

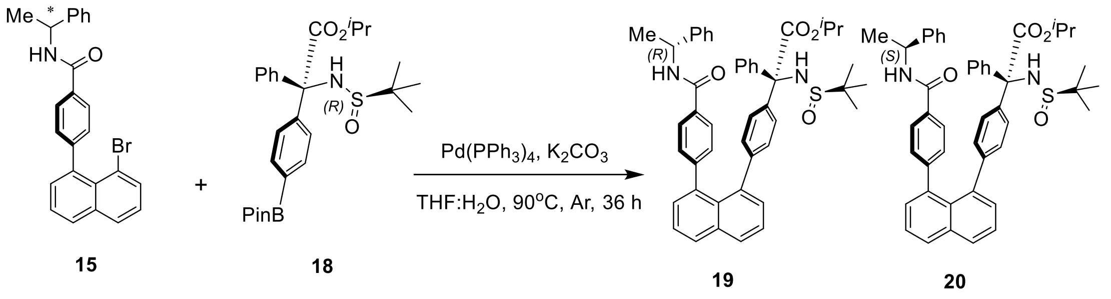

The substrates **15** were prepared according to the reported procedures and their characterization data is in agreement with the literature.[3] The substrate **18** was prepared according to the general procedure D by using isopropyl 2-oxo-2-phenylacetate as the starting material.

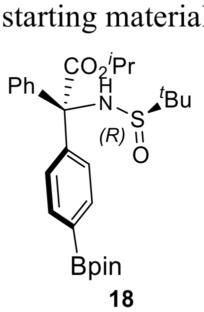

18

isopropyl (*R*)-2-(((*R*)-tert-butylsulfinyl)amino)-2-phenyl-2-(4-(4,4,5,5-tetramethyl-1,3,2-dioxaborolan-2-yl)phenyl)acetate 18: Purified by using a flash column chromatography (PE/EA = 5/1); isolated yield = 56%; pale yellow oil. ^{1}H NMR (400 MHz, $CDCl_3$) δ 7.81–7.73 (m, 2H), 7.55–7.49 (m, 2H), 7.37–7.30 (m, 5H), 5.28 (s, 1H), 5.15–5.03 (m, 1H), 1.34 (s, 12H), 1.21–1.13 (m, 15H) ppm; ^{13}C NMR (101 MHz, $CDCl_3$) δ 171.9, 143.9, 138.9, 134.3, 129.9, 129.2, 128.9, 128.3, 127.9, 83.9, 72.4, 70.6, 56.5, 30.9, 24.9, 24.8, 22.8, 21.3 ppm.

15 (0.21 mmol, 90mg, 1.0 equiv.), **18** (0.21 mmol, 105 mg, 1.0 equiv.), $Pd(PPh_3)_4$ (23 mg, 0.02 mmol, 10 mol%), K_2CO_3 (0.63 mmol, 87 mg, 3.0 equiv.) were dissolved in THF:H_2O (10:2 mL) under Ar atmosphere and the mixture was heated to 90 °C for 36 h. After being cooled down to room temperature, 10 mL of water was added, and the solution was extracted three times with each 20 mL of ethyl acetate. The combined organic layers were washed with brine, dried over anhydrous Na_2SO_4 and concentrated under vacuum. The crude products were purified by column chromatography (silica gel, *n*-hexane/EtOAc = 2:1) to obtain the desired product **19** as a white solid. The amide product **20** of the opposite configuration is also obtained by the same general procedure.

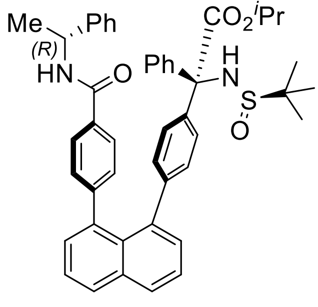

19

isopropyl (*R*)-2-(((*R*)-*tert*-butylsulfinyl)amino)-2-phenyl-2-(4-(8-(4-(((*R*)-1-phenylethyl)carbamoyl)phenyl)naphthalen-1-yl)phenyl)acetate 19: $[\alpha]_D^{25}$ = –37 (*c* = 0.1, THF); white solid, yield 76%, ^{1}H NMR (400 MHz, $CDCl_3$) δ 7.94 (dd, *J* = 8.2, 1.3 Hz, 2H), 7.57–7.44 (m, 4H), 7.42–7.33 (m, 7H), 7.32–7.25 (m, 4H), 7.24–7.19 (m, 1H), 7.06 (d, *J* = 8.7 Hz, 2H), 6.99–6.92 (m, 4H), 5.32 (p, *J* = 7.0 Hz, 1H), 5.04 (s, 1H), 4.99 (p, *J* = 6.2 Hz, 1H), 1.61 (d, *J* = 6.9 Hz, 3H), 1.24–1.18 (m, 12H), 1.12 (d, *J* = 6.2 Hz, 3H) ppm; ^{13}C NMR (101 MHz, $CDCl_3$) δ 172.0, 166.7, 146.8, 143.8, 143.1, 139.8, 139.6, 139.2, 138.4, 135.5, 132.7, 131.2, 130.8, 129.8, 129.7, 129.6, 129.4, 129.2, 129.1, 128.8, 128.55, 128.47, 128.3, 128.21, 128.16, 128.1, 128.0, 127.9, 127.0, 126.4, 126.2, 126.1, 125.23, 125.16, 74.9, 72.5, 70.4, 56.5, 49.4, 22.9, 21.9, 21.4, 21.3 ppm. HRMS (ESI-TOF) *m/z* $[C_{43}H_{41}NO_3S +H]^+$ calcd for 723.3257, found 723.3257.

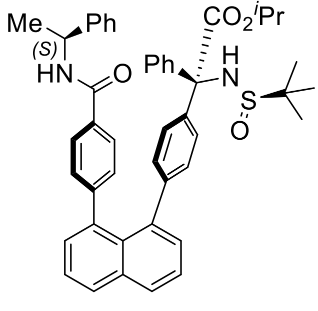

20

isopropyl (*R*)-2-(((*R*)-*tert*-butylsulfinyl)amino)-2-phenyl-2-(4-(8-(4-(((*S*)-1-phenylethyl)carbamoyl)phenyl)naphthalen-1-yl)phenyl)acetate: $[\alpha]_D^{22}$ = 80.5 (*c* = 0.2, $CHCl_3$); white solid, yield 73%, ^{1}H NMR (400 MHz, $CDCl_3$) δ 7.95 (dd, *J* = 8.2, 1.3 Hz, 2H), 7.62 – 7.52 (m, 3H), 7.45 – 7.31 (m, 12H), 7.25 (tt, *J* = 6.3, 1.5 Hz, 1H), 7.17 – 7.09 (m, 2H), 6.99 (dt, *J* = 8.2, 4.6 Hz, 2H), 6.90 (dd, *J* = 11.6, 8.0 Hz, 2H), 6.79 (d, *J* = 10.1 Hz, 1H), 5.33 – 5.26 (m, 1H), 5.08 – 5.01 (m, 1H), 4.99 (s, 1H), 1.45 (d, *J*

= 6.9 Hz, 3H), 1.28 (d, *J* = 6.2 Hz, 3H), 1.16 (s, 9H), 1.13 (d, *J* = 6.3 Hz, 3H) ppm; ^{13}C NMR (101 MHz, $CDCl_3$) δ 171.8, 166.9, 146.5, 143.6, 142.9, 139.4, 139.2, 139.1, 138.1, 135.3, 132.4, 131.4, 131.0, 129.8, 129.6, 129.3, 129.0, 128.9, 128.8, 128.6, 128.5, 128.2, 128.0, 127.1, 126.6, 126.2, 125.5, 125.25, 125.20, 72.7, 70.3, 56.5, 49.1, 22.7, 21.7, 21.6, 21.3 ppm. HRMS (ESI-TOF) *m*/*z* $[C_{43}H_{41}NO_3S + H]^+$ calcd for 723.3257, found 723.3304.

8. References

1. Tu, G.; Yan, Y.; Chen, X.-Y.; Lv, Q.-L.; Wang, J.-Q.; Li, S.-H.; *Drug Discoveries & Therapeutics* **2013**, *7*, 58.

2. Thakellapalli, H.; Farajidizaji, B.; Butcher, T. W.; Akhmedov, N. G.; Popp, B. V.; Petersen, J. L.; Wang, K. K. *Org. Lett.* **2015**, *17*, 3470.

3. Jin, S.; Wang, Y.; Tang, Y.; Wang, J.-Y.; Xu, T.; Pan, J.; Zhang, S.; Yuan, Q.; Rahman, A. U.; McDonald, J. D.; Wang, G.-Q.; Li, S.; Li, G. *Research* **2022**, *2022*, 0013.

4. Wang, Y.; Xu, T.; Jin, S.; Wang, J. Y.; Yuan, Q.; Liu, H.; Tang, Y.; Zhang, S.; Yan, W.; Jiao, Y.; Li, G. *Chem. Eur. J.* **2024**, *30,* e202400005.

9. Copies of ^{1}H and ^{13}C spectra of compounds

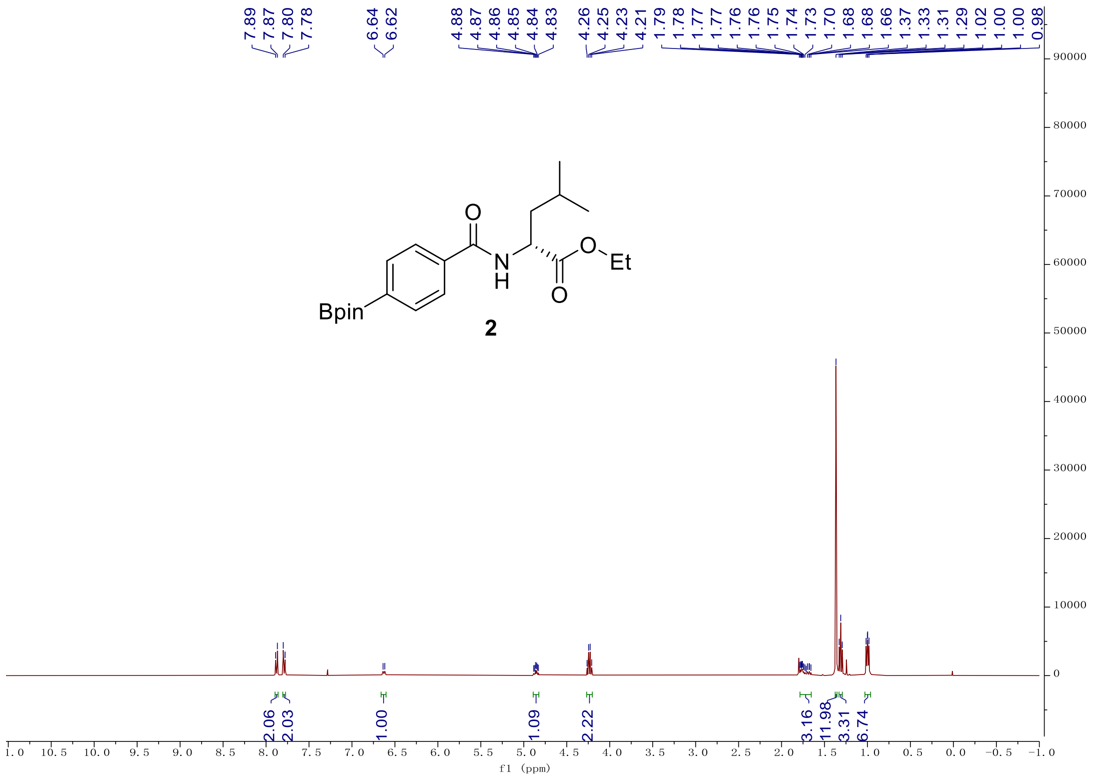

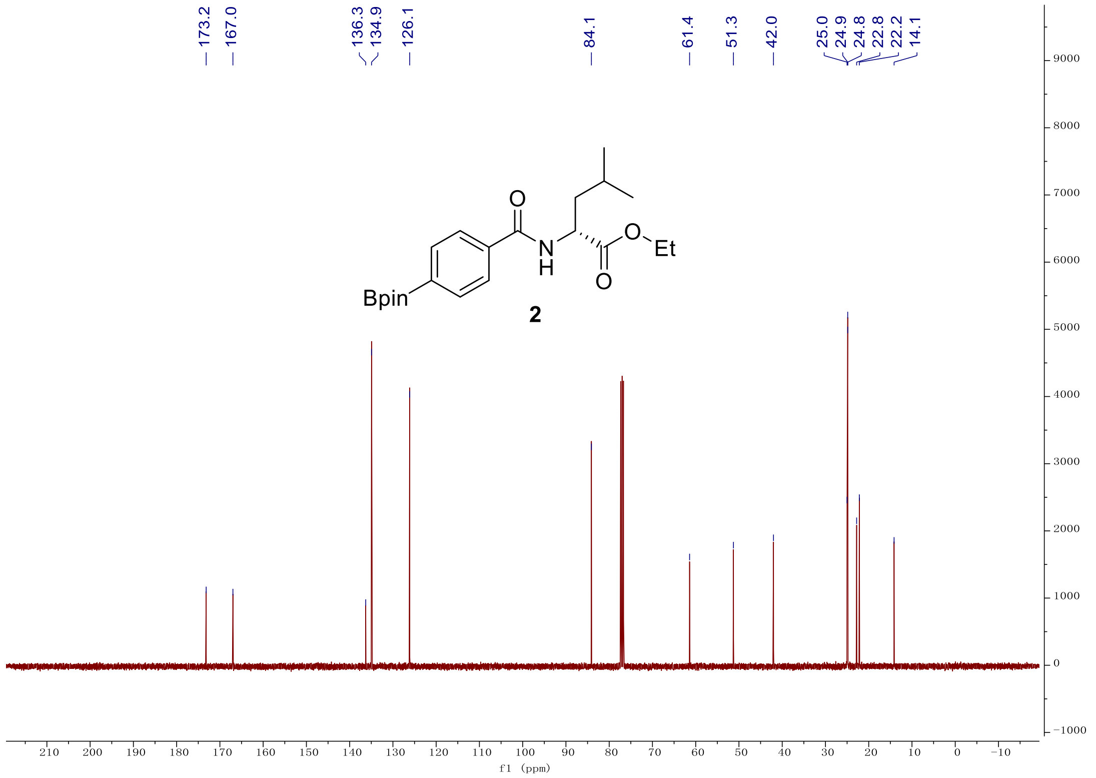

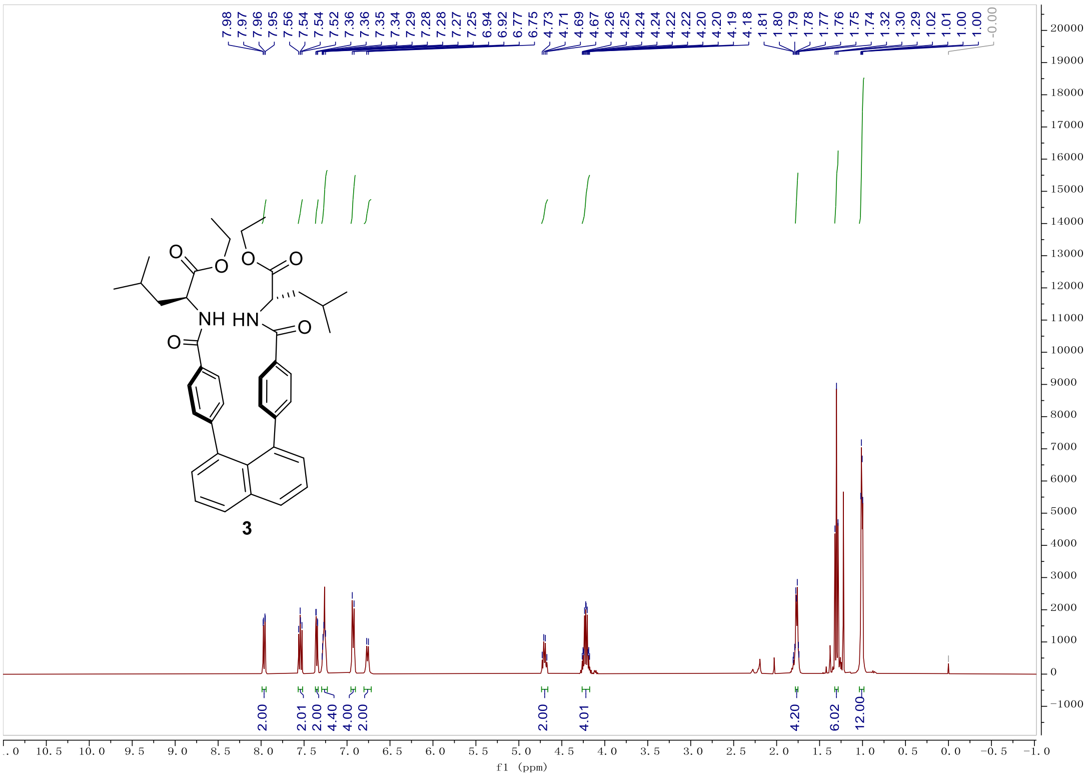
7.98 7.97 7.96 7.95 7.56 7.54 7.54 7.52 7.36 7.36 7.35 7.34 7.29 7.28 7.28 7.27 7.25 6.94 6.92 6.77 6.75 4.73 4.71 4.69 4.67 4.26 4.25 4.24 4.24 4.22 4.22 4.20 4.20 4.19 4.18 1.81 1.80 1.79 1.78 1.77 1.76 1.75 1.74 1.32 1.30 1.29 1.02 1.01 1.00 1.00 -0.00
2.00 2.01 2.00 4.40 4.00 2.00 2.00 4.01 4.20 6.02 12.00
O
O
NH
HN
3
f1 (ppm)

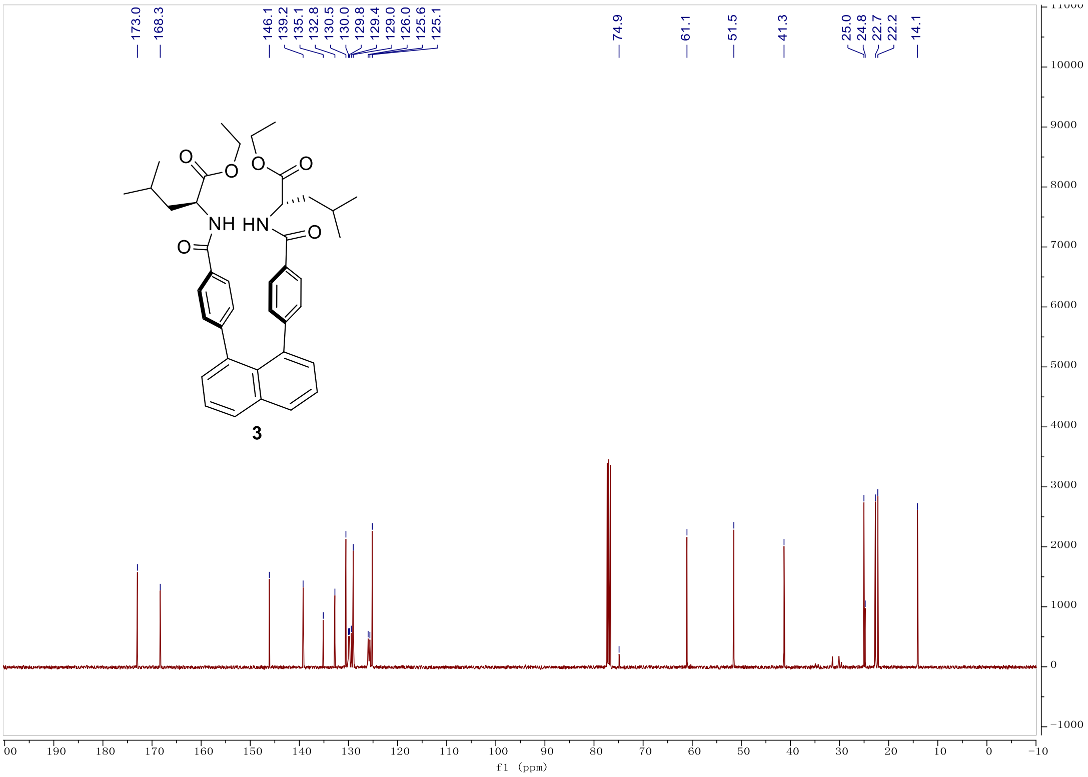
173.0 168.3 146.1 139.2 135.1 132.8 130.5 130.0 129.8 129.4 129.0 126.0 125.6 125.1 74.9 61.1 51.5 41.3 25.0 24.8 22.7 22.2 14.1
O
O
NH
HN
3
f1 (ppm)

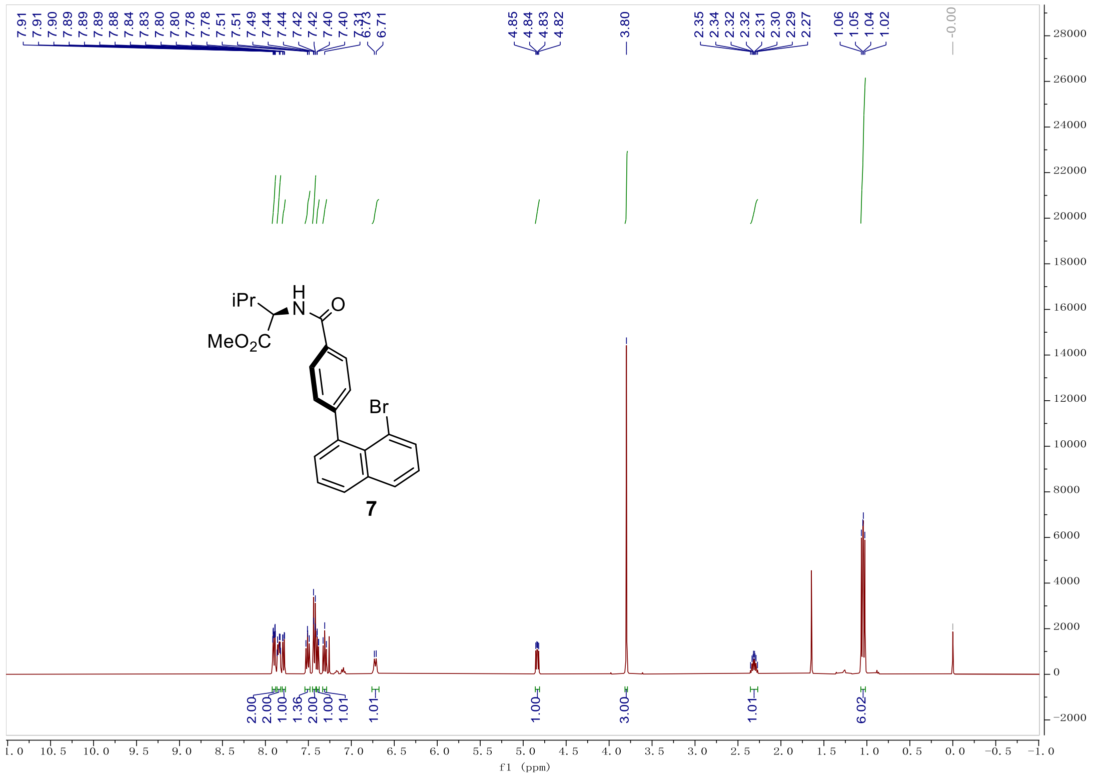

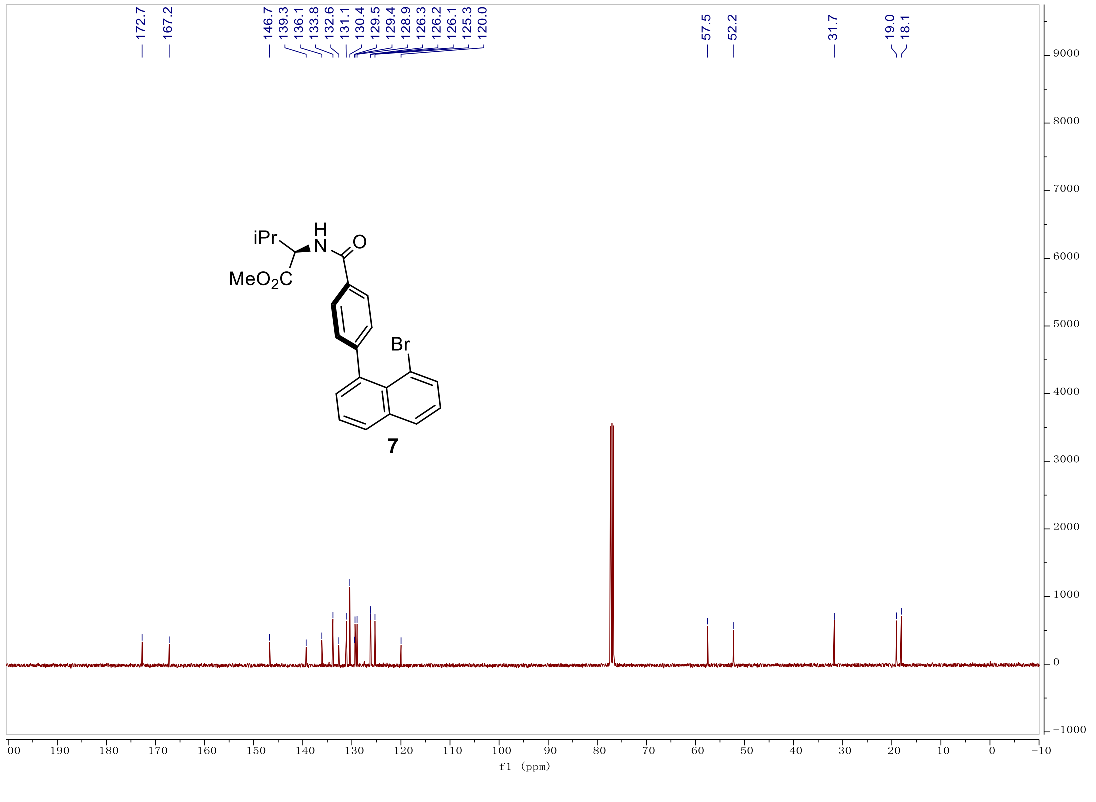

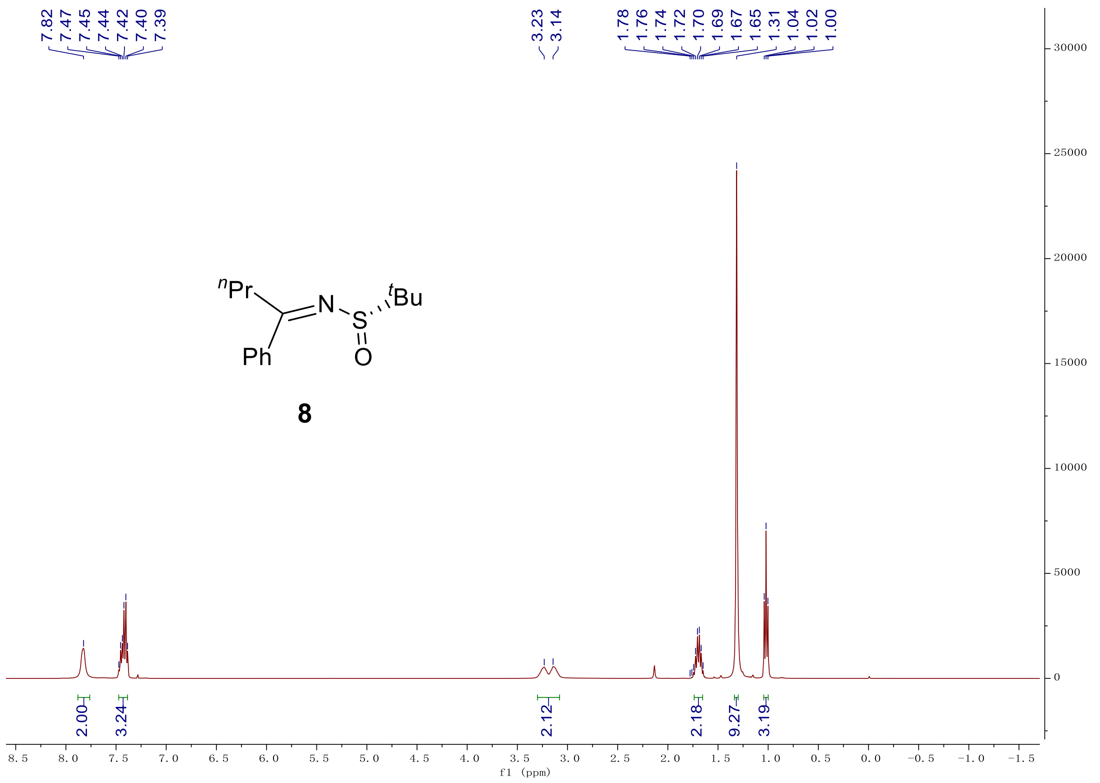
7.82
7.47
7.45
7.44
7.42
7.40
7.39
3.23
3.14
1.78
1.76
1.74
1.72
1.70
1.69
1.67
1.65
1.31
1.04
1.02
1.00
^{n}Pr
N
S
^{t}Bu
Ph
O
8
2.00
3.24
2.12
2.18
9.27
3.19
f1 (ppm)

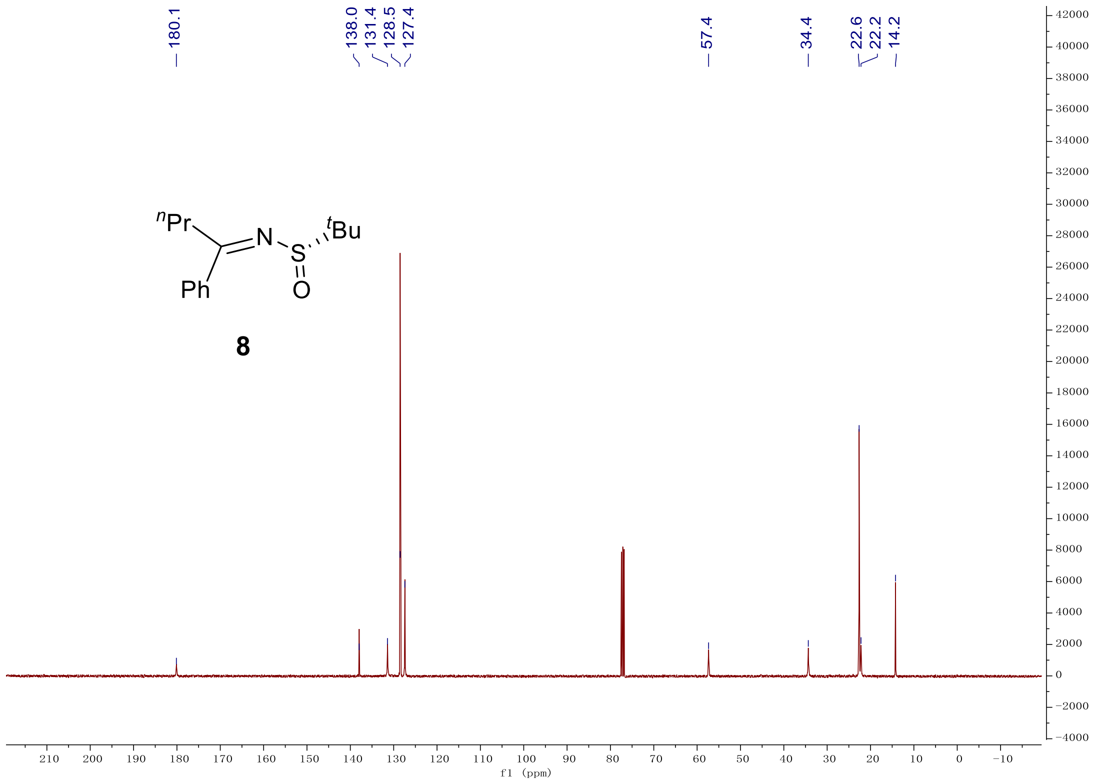
180.1
138.0
131.4
128.5
127.4
57.4
34.4
22.6
22.2
14.2
^{n}Pr
N
S
^{t}Bu
Ph
O
8
f1 (ppm)

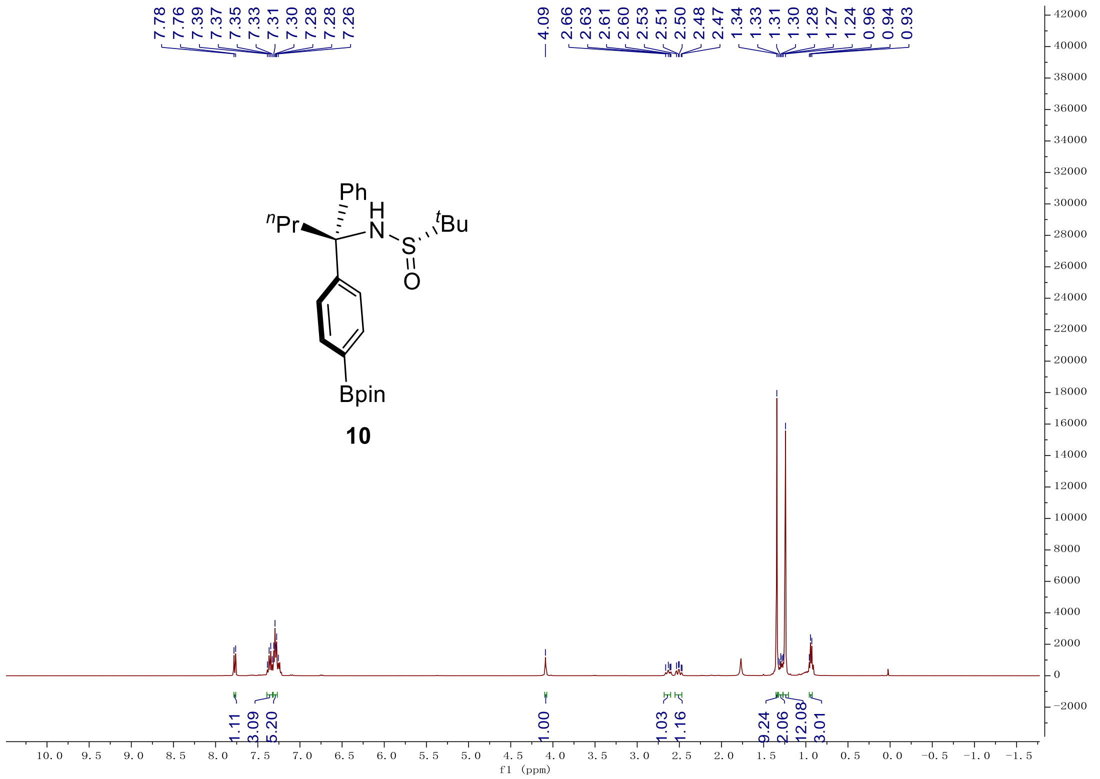
7.78 7.76 7.39 7.37 7.35 7.33 7.31 7.30 7.28 7.28 7.26
4.09
2.66 2.63 2.61 2.60 2.53 2.51 2.50 2.48 2.47
1.34 1.33 1.31 1.30 1.28 1.27 1.24 0.96 0.94 0.93
Ph
H
N
S
O
^{n}Pr
^{t}Bu
Bpin
10
1.11 3.09 5.20 1.00 1.03 1.16 9.24 2.06 12.08 3.01
f1 (ppm)

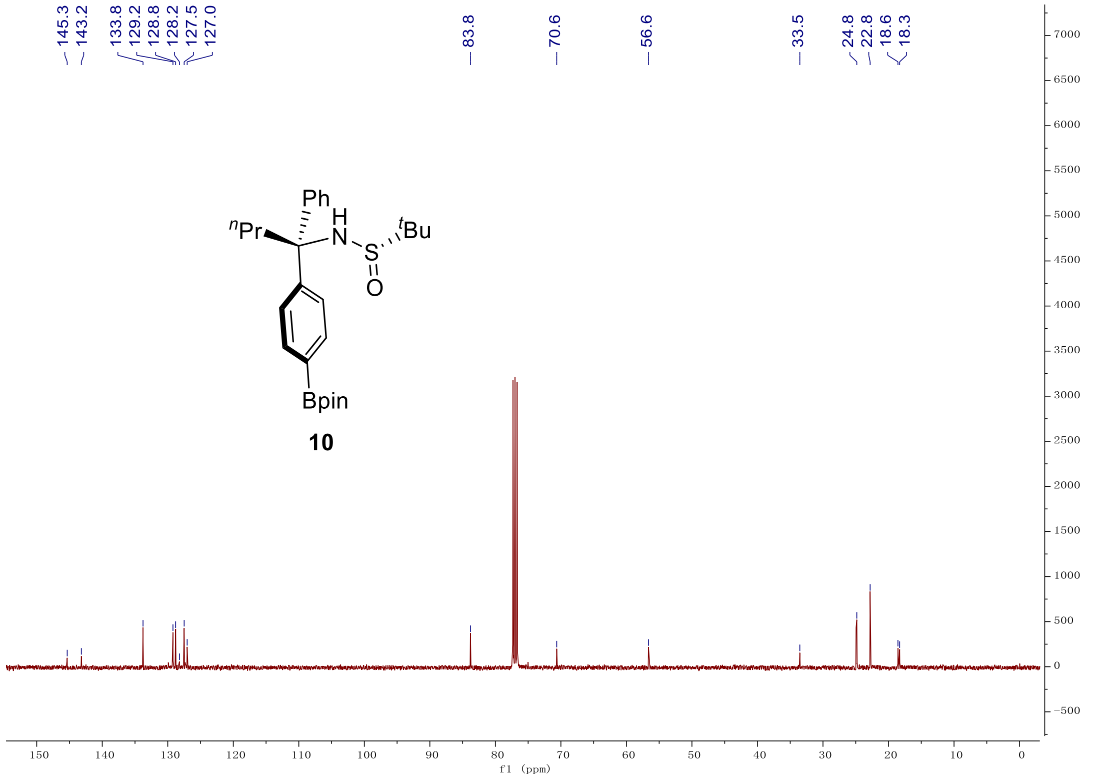
145.3 143.2 133.8 129.2 128.8 128.2 127.5 127.0
83.8
70.6
56.6
33.5
24.8 22.8 18.6 18.3
Ph
H
N
S
O
^{n}Pr
^{t}Bu
Bpin
10
f1 (ppm)

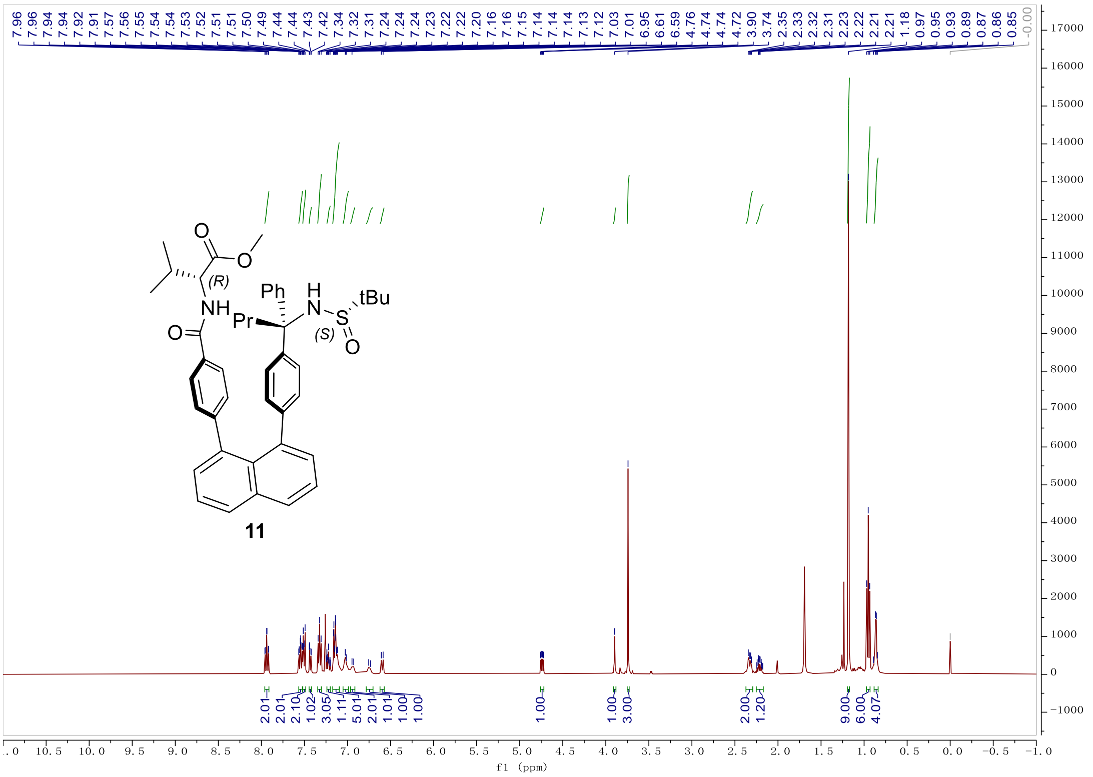
Ph
H
N
S
tBu
Pr
(R)
(S)
NH
O
11
f1 (ppm)

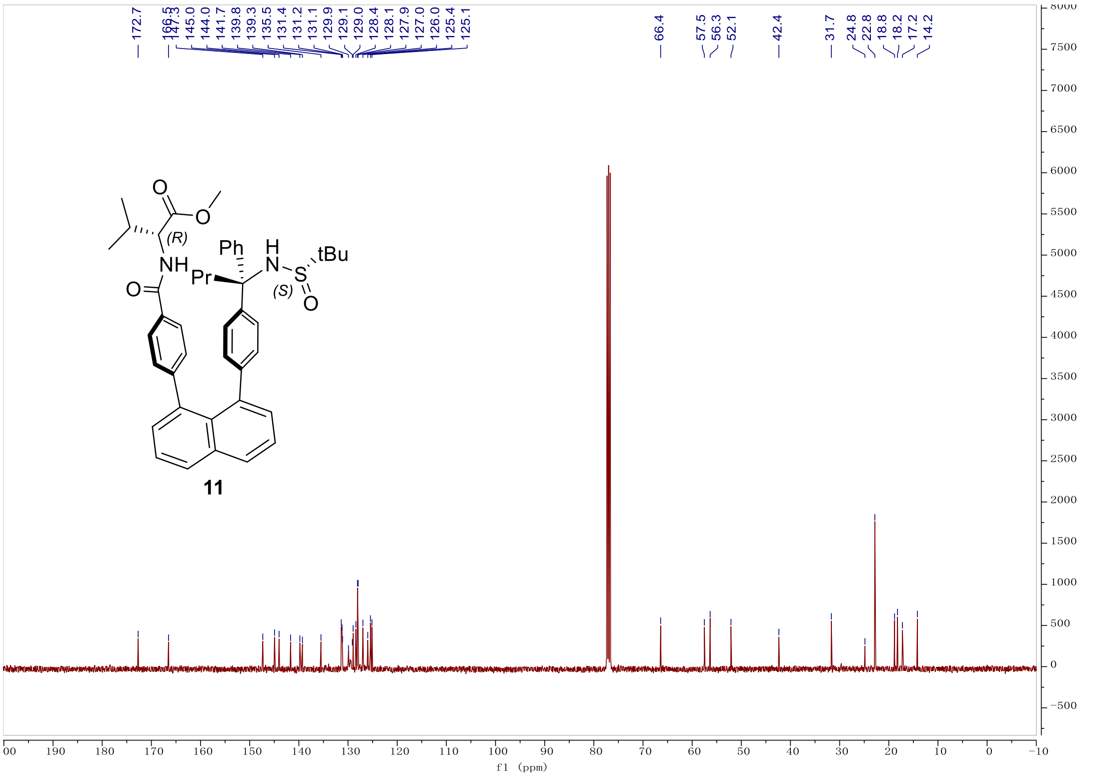
Ph
H
N
S
tBu
Pr
(R)
(S)
NH
O
11
f1 (ppm)

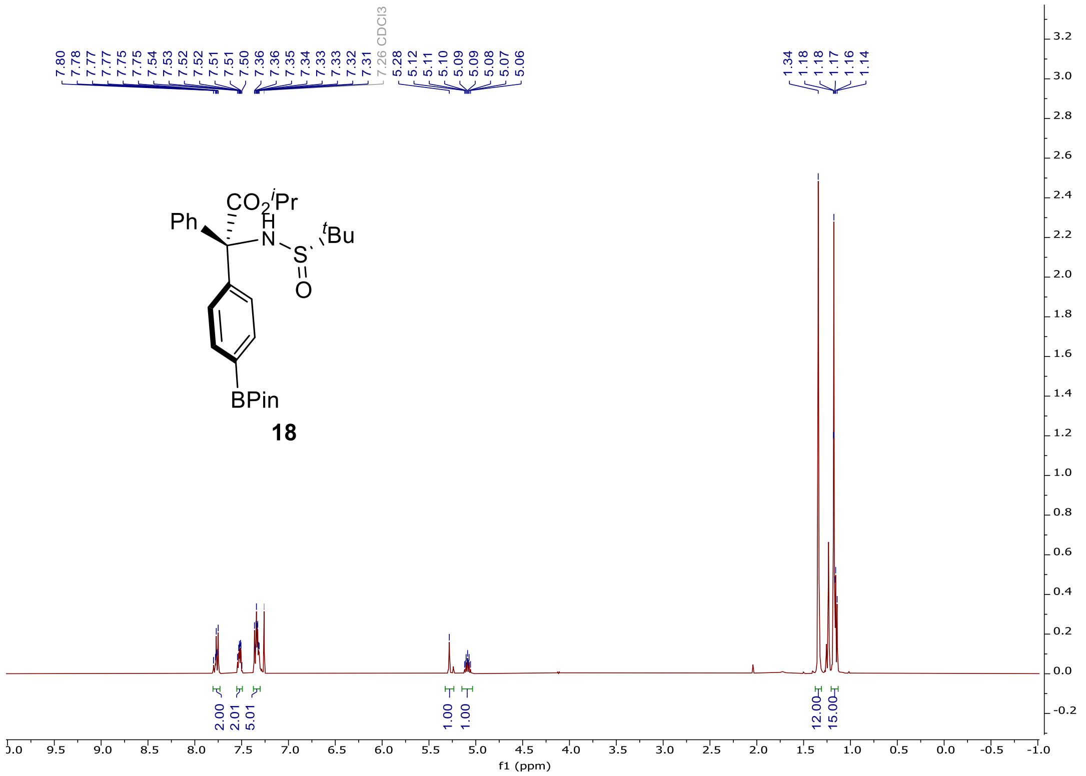

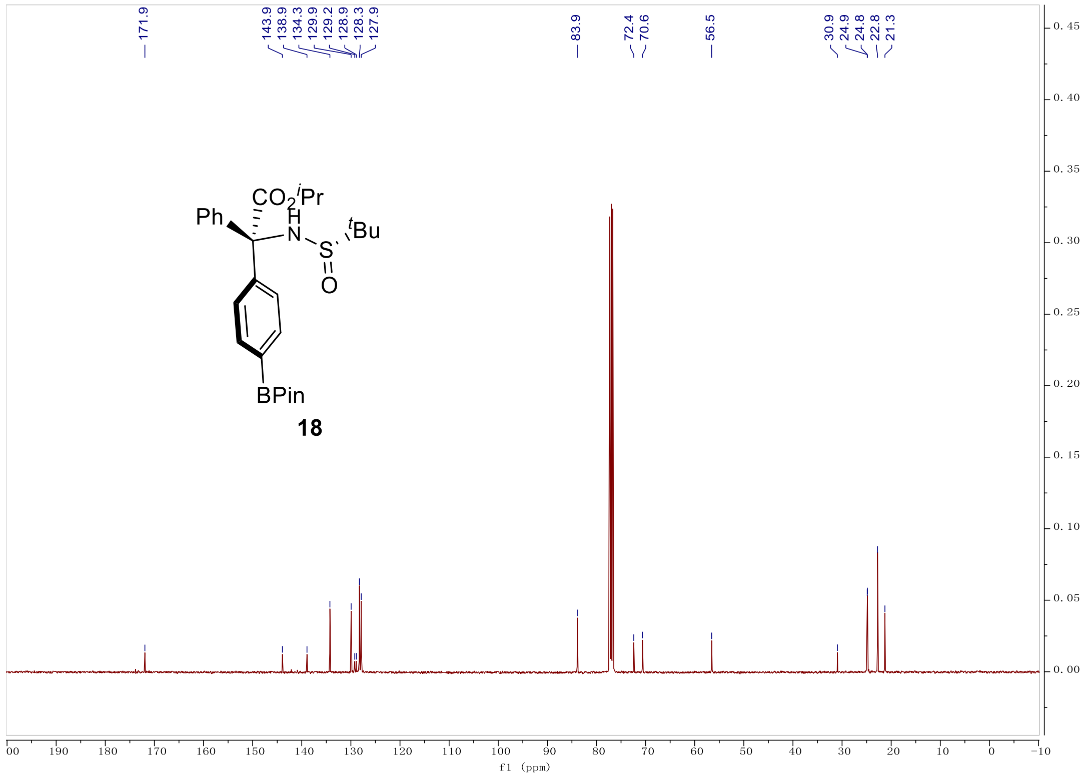

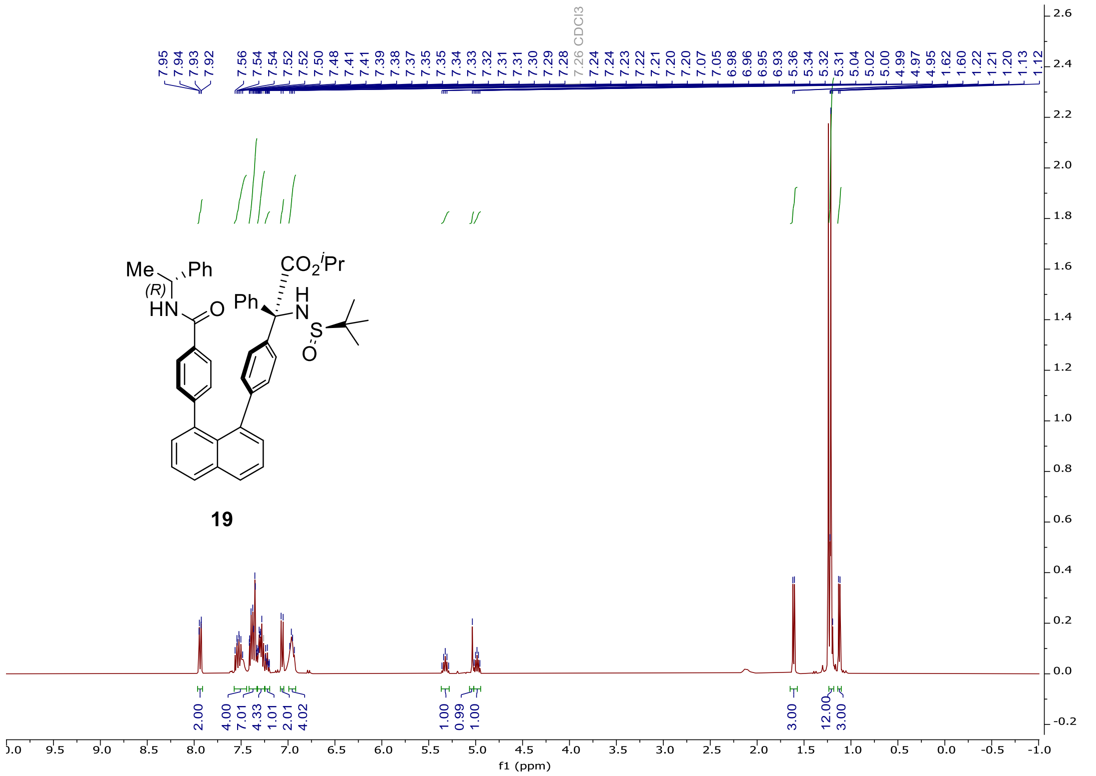

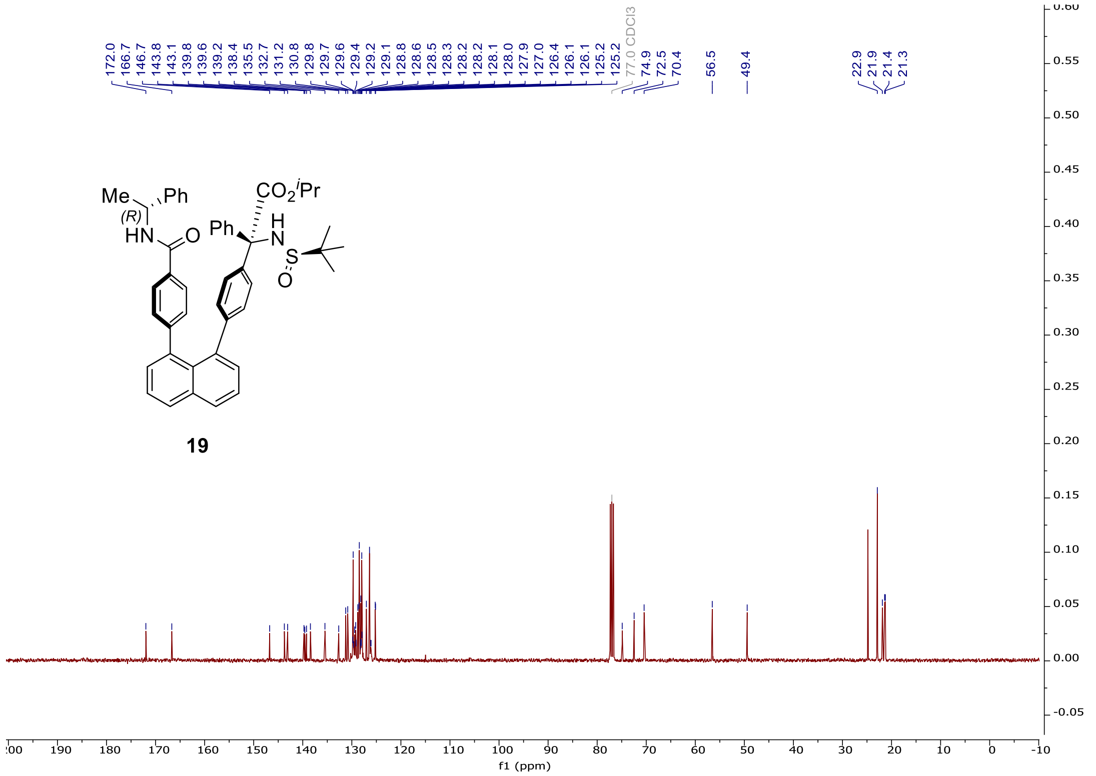

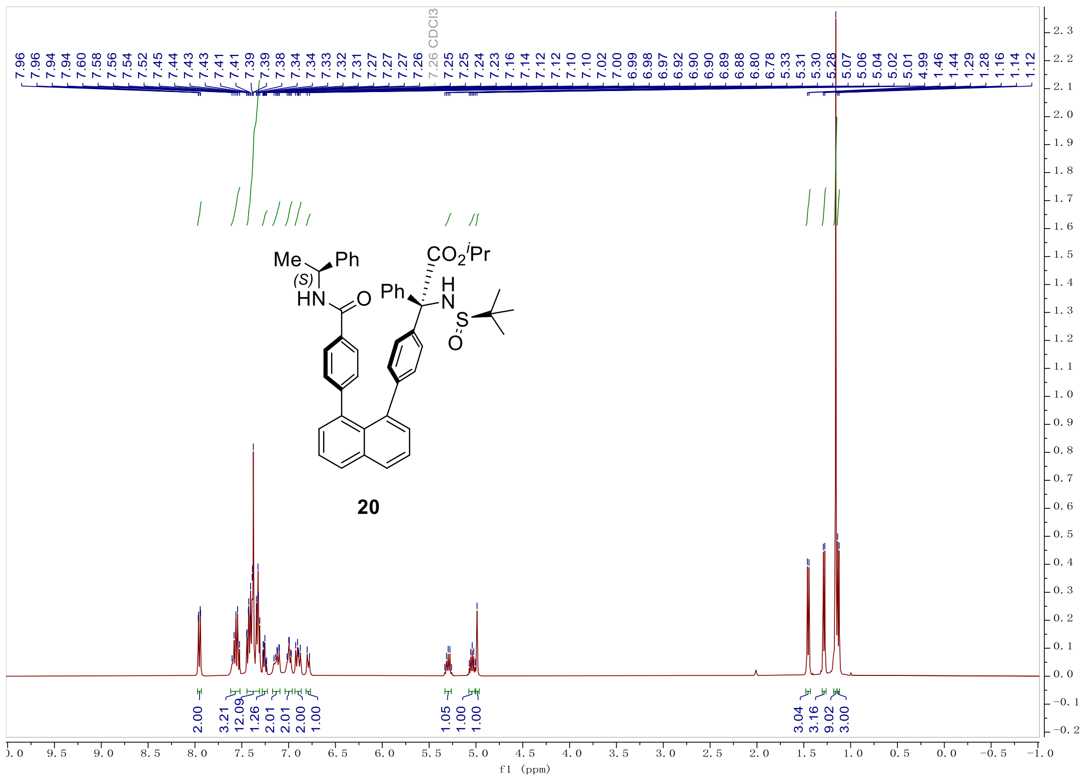

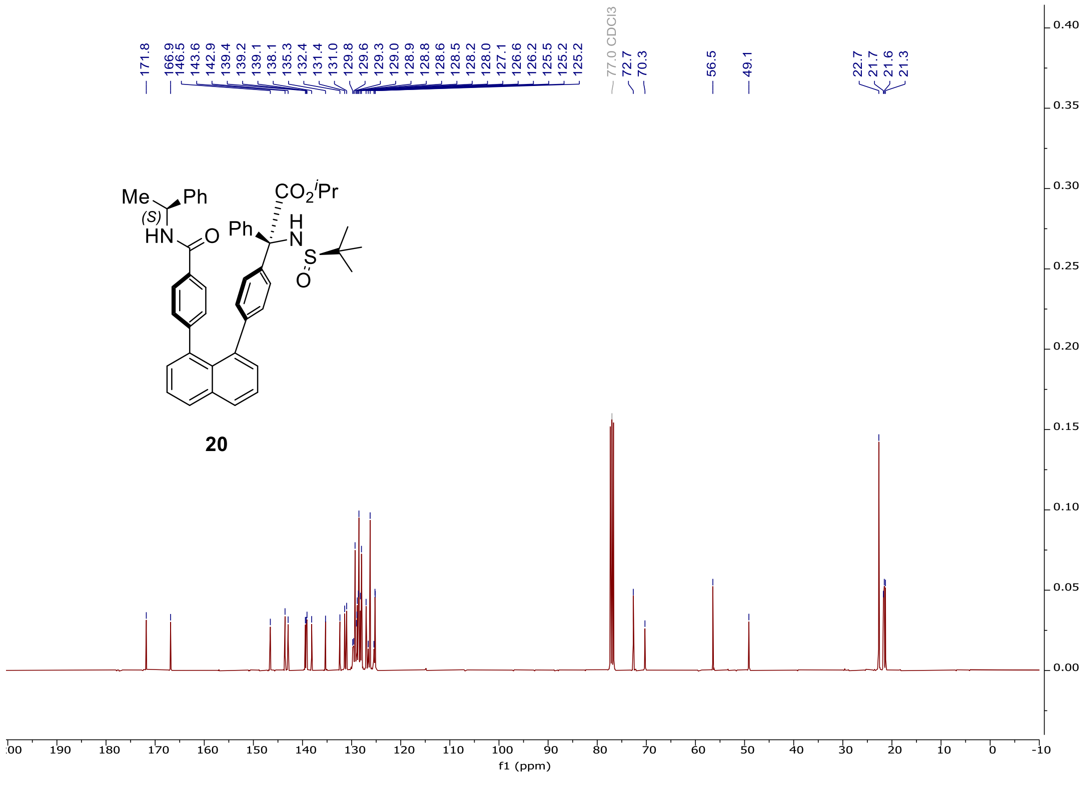

10. X-ray Diffraction Data

-----Write up for **3** and **3’**

Table 1. Crystal data and structure refinement for **3** and **3’**.

Identification code	230519a	
Empirical formula	C40 H46 N2 O6	
Formula weight	650.79	
Temperature	293(2) K	
Wavelength	1.54178 Å	
Crystal system	Monoclinic	
Space group	P2(1)	
Unit cell dimensions	a = 15.8265(9) Å	α= 90°.
	b = 9.8500(5) Å	β= 98.857(5)°.
	c = 23.6988(12) Å	γ = 90°.
Volume	3650.4(3) $Å^3$	
Z	4	
Density (calculated)	1.184 Mg/m^3	
Absorption coefficient	0.635 mm^{-1}	
F(000)	1392	
Crystal size	0.35 x 0.10 x 0.03 mm^3	
Theta range for data collection	3.15 to 66.05°.	
Index ranges	-15<=h<=18, -7<=k<=11, -28<=l<=27	
Reflections collected	13093	
Independent reflections	8856 [R(int) = 0.0450]	
Completeness to theta = 66.05°	99.9 %	
Absorption correction	Semi-empirical from equivalents	
Max. and min. transmission	0.9812 and 0.8083	
Refinement method	Full-matrix least-squares on F^2	
Data / restraints / parameters	8856 / 408 / 890	
Goodness-of-fit on F^2	1.050	
Final R indices [I>2sigma(I)]	R1 = 0.0643, wR2 = 0.1398	
R indices (all data)	R1 = 0.1061, wR2 = 0.1654	
Absolute structure parameter	0.0(4)	
Extinction coefficient	0.00178(14)	
Largest diff. peak and hole	0.355 and -0.235 e.$Å^{-3}$	

Table 2. Atomic coordinates (x 10^4) and equivalent isotropic displacement parameters ($Å^2$x 10^3)
for 230519a. U(eq) is defined as one third of the trace of the orthogonalized U^{ij} tensor.

	x	y	z	U(eq)
N(1)	8011(3)	2495(5)	7608(2)	54(1)
N(2)	3376(3)	1947(5)	7938(2)	64(1)
N(3)	6853(3)	2005(6)	2086(2)	69(2)
N(4)	11867(3)	2377(5)	2543(2)	57(1)
O(1)	9766(3)	4529(5)	8185(2)	92(2)
O(2)	9598(3)	3340(6)	7384(2)	98(2)
O(3)	7580(3)	4611(4)	7422(2)	76(1)
O(4)	1852(3)	742(6)	7751(2)	101(2)
O(5)	1527(4)	1673(8)	8544(3)	159(3)
O(6)	3830(3)	-163(4)	7758(2)	64(1)
O(7)	7313(4)	3290(7)	1157(2)	114(2)
O(8)	6729(5)	1703(9)	598(3)	164(3)
O(9)	7337(3)	-77(4)	2343(2)	72(1)
O(10)	13248(3)	4471(5)	1909(2)	83(1)
O(11)	13490(3)	3560(6)	2761(2)	95(2)
O(12)	11577(2)	4570(4)	2631(2)	55(1)
C(1)	9383(4)	3606(7)	7823(3)	67(2)
C(2)	8663(3)	2932(6)	8067(2)	57(1)
C(3)	8986(4)	1728(7)	8449(2)	67(2)
C(4)	9647(4)	2022(7)	8967(3)	72(2)
C(5)	9956(5)	703(9)	9262(3)	114(3)
C(6)	9342(6)	3000(11)	9375(3)	142(4)
C(7)	10550(5)	5140(10)	8048(4)	121(3)
C(8)	11015(7)	5658(14)	8561(5)	189(6)
C(9)	2049(5)	1339(9)	8263(4)	89(2)
C(10)	2995(4)	1511(7)	8433(3)	71(2)
C(11)	3191(5)	2483(8)	8937(3)	93(2)
C(12)	4029(7)	2341(10)	9267(4)	122(3)
C(13)	4119(7)	3330(11)	9785(4)	174(5)
C(14)	4295(7)	974(10)	9527(4)	146(4)
C(15)	951(5)	372(10)	7547(4)	110(3)
C(16)	781(5)	-973(11)	7813(4)	127(3)

C(17)	7522(4)	3394(6)	7290(2)	55(2)
C(18)	6918(3)	2941(6)	6777(2)	46(1)
C(19)	6953(3)	1653(6)	6540(2)	53(1)
C(20)	6446(4)	1341(6)	6024(2)	59(2)
C(21)	5895(3)	2288(6)	5744(2)	47(1)
C(22)	5830(3)	3548(6)	5994(2)	53(1)
C(23)	6342(3)	3877(6)	6499(2)	51(1)
C(24)	3702(3)	1025(7)	7606(3)	56(2)
C(25)	3868(3)	1510(6)	7039(2)	51(1)
C(26)	3659(4)	2784(7)	6822(3)	68(2)
C(27)	3729(4)	3110(7)	6263(3)	68(2)
C(28)	4022(3)	2175(6)	5902(2)	52(1)
C(29)	4268(4)	901(7)	6124(3)	59(2)
C(30)	4190(4)	583(6)	6682(2)	58(2)
C(31)	5463(3)	2029(6)	5140(2)	51(1)
C(32)	6004(4)	1740(7)	4759(3)	66(2)
C(33)	5734(5)	1740(7)	4164(3)	77(2)
C(34)	4915(5)	2090(7)	3961(3)	73(2)
C(35)	4330(4)	2319(6)	4326(3)	63(2)
C(36)	4576(3)	2270(6)	4936(2)	54(1)
C(37)	3932(3)	2460(6)	5287(2)	55(1)
C(38)	3128(4)	2829(8)	5030(3)	79(2)
C(39)	2879(5)	2904(8)	4440(3)	84(2)
C(40)	3460(5)	2648(7)	4098(3)	77(2)
C(41)	6872(6)	2322(10)	1082(3)	91(2)
C(42)	6438(5)	1610(8)	1535(3)	82(2)
C(43)	5491(5)	2016(10)	1429(3)	99(2)
C(44)	4908(7)	1207(14)	1750(4)	149(4)
C(45)	5063(6)	1297(12)	2386(4)	141(4)
C(46)	4589(9)	25(13)	1535(5)	199(5)
C(47)	7309(8)	1995(15)	173(5)	182(5)
C(48)	6763(9)	2780(20)	-223(7)	282(8)
C(49)	13084(4)	3580(7)	2299(3)	64(2)
C(50)	12375(3)	2608(7)	2082(2)	59(2)
C(51)	11832(4)	2934(7)	1513(2)	73(2)
C(52)	12225(6)	2515(9)	980(3)	74(2)
C(53)	11772(8)	3219(14)	446(4)	131(5)
C(54)	12192(12)	979(12)	893(8)	143(4)

C(52')	11538(12)	1663(15)	1183(7)	74(2)
C(53')	10859(16)	2080(30)	682(9)	131(5)
C(54')	12300(20)	970(40)	974(19)	143(4)
C(55)	13977(5)	5338(10)	2088(4)	116(3)
C(56)	13999(7)	6356(13)	1648(5)	197(6)
C(57)	7293(4)	1118(7)	2454(3)	57(2)
C(58)	7688(3)	1681(6)	3020(2)	52(1)
C(59)	8123(4)	812(7)	3414(3)	62(2)
C(60)	8461(4)	1269(7)	3954(3)	63(2)
C(61)	8391(3)	2601(6)	4124(2)	53(1)
C(62)	7964(4)	3479(7)	3721(3)	66(2)
C(63)	7615(4)	3006(7)	3179(3)	67(2)
C(64)	11513(3)	3401(6)	2795(2)	45(1)
C(65)	11059(3)	3063(6)	3285(2)	46(1)
C(66)	10773(3)	4126(6)	3594(2)	51(1)
C(67)	10428(3)	3882(6)	4085(2)	53(1)
C(68)	10346(3)	2578(6)	4279(2)	51(1)
C(69)	10578(4)	1519(6)	3956(3)	66(2)
C(70)	10925(4)	1756(7)	3457(3)	64(2)
C(71)	8610(3)	3027(6)	4731(2)	54(2)
C(72)	7957(4)	3566(8)	4976(3)	75(2)
C(73)	7995(4)	3716(9)	5562(3)	87(2)
C(74)	8713(4)	3284(7)	5915(3)	75(2)
C(75)	9436(4)	2814(6)	5691(2)	60(2)
C(76)	9396(3)	2731(6)	5083(2)	53(2)
C(77)	10170(3)	2365(6)	4879(2)	52(1)
C(78)	10866(4)	1954(7)	5266(3)	69(2)
C(79)	10875(4)	2006(7)	5859(3)	74(2)
C(80)	10179(4)	2468(7)	6068(3)	70(2)

Table 3. Bond lengths [Å] and angles [°] for 230519a.

N(1)-C(17)	1.331(7)
N(1)-C(2)	1.444(6)
N(1)-H(1)	0.8600
N(2)-C(24)	1.355(7)
N(2)-C(10)	1.464(7)
N(2)-H(2)	0.8600
N(3)-C(57)	1.349(7)
N(3)-C(42)	1.424(7)
N(3)-H(3)	0.8600
N(4)-C(64)	1.338(6)
N(4)-C(50)	1.470(6)
N(4)-H(4)	0.8600
O(1)-C(1)	1.329(7)
O(1)-C(7)	1.459(8)
O(2)-C(1)	1.175(7)
O(3)-C(17)	1.238(7)
O(4)-C(9)	1.339(9)
O(4)-C(15)	1.478(9)
O(5)-C(9)	1.186(8)
O(6)-C(24)	1.232(7)
O(7)-C(41)	1.179(9)
O(8)-C(41)	1.287(9)
O(8)-C(47)	1.492(12)
O(9)-C(57)	1.211(7)
O(10)-C(49)	1.330(7)
O(10)-C(55)	1.446(8)
O(11)-C(49)	1.181(7)
O(12)-C(64)	1.225(6)
C(1)-C(2)	1.508(8)
C(2)-C(3)	1.531(8)
C(2)-H(2A)	0.9800
C(3)-C(4)	1.513(8)
C(3)-H(3A)	0.9700
C(3)-H(3B)	0.9700
C(4)-C(6)	1.496(10)
C(4)-C(5)	1.520(9)

C(4)-H(4A)	0.9800
C(5)-H(5A)	0.9600
C(5)-H(5B)	0.9600
C(5)-H(5C)	0.9600
C(6)-H(6A)	0.9600
C(6)-H(6B)	0.9600
C(6)-H(6C)	0.9600
C(7)-C(8)	1.416(11)
C(7)-H(7A)	0.9700
C(7)-H(7B)	0.9700
C(8)-H(8A)	0.9600
C(8)-H(8B)	0.9600
C(8)-H(8C)	0.9600
C(9)-C(10)	1.499(10)
C(10)-C(11)	1.526(9)
C(10)-H(10)	0.9800
C(11)-C(12)	1.439(11)
C(11)-H(11A)	0.9700
C(11)-H(11B)	0.9700
C(12)-C(14)	1.513(12)
C(12)-C(13)	1.557(11)
C(12)-H(12)	0.9800
C(13)-H(13A)	0.9600
C(13)-H(13B)	0.9600
C(13)-H(13C)	0.9600
C(14)-H(14A)	0.9600
C(14)-H(14B)	0.9600
C(14)-H(14C)	0.9600
C(15)-C(16)	1.509(12)
C(15)-H(15A)	0.9700
C(15)-H(15B)	0.9700
C(16)-H(16A)	0.9600
C(16)-H(16B)	0.9600
C(16)-H(16C)	0.9600
C(17)-C(18)	1.493(7)
C(18)-C(23)	1.390(7)
C(18)-C(19)	1.393(7)
C(19)-C(20)	1.390(7)

C(19)-H(19)	0.9300
C(20)-C(21)	1.376(8)
C(20)-H(20)	0.9300
C(21)-C(22)	1.386(7)
C(21)-C(31)	1.509(7)
C(22)-C(23)	1.376(7)
C(22)-H(22)	0.9300
C(23)-H(23)	0.9300
C(24)-C(25)	1.488(8)
C(25)-C(26)	1.377(8)
C(25)-C(30)	1.394(7)
C(26)-C(27)	1.383(8)
C(26)-H(26)	0.9300
C(27)-C(28)	1.384(8)
C(27)-H(27)	0.9300
C(28)-C(29)	1.392(8)
C(28)-C(37)	1.470(7)
C(29)-C(30)	1.382(7)
C(29)-H(29)	0.9300
C(30)-H(30)	0.9300
C(31)-C(32)	1.366(8)
C(31)-C(36)	1.433(7)
C(32)-C(33)	1.410(8)
C(32)-H(32)	0.9300
C(33)-C(34)	1.355(9)
C(33)-H(33)	0.9300
C(34)-C(35)	1.381(8)
C(34)-H(34)	0.9300
C(35)-C(40)	1.436(9)
C(35)-C(36)	1.438(7)
C(36)-C(37)	1.425(8)
C(37)-C(38)	1.373(8)
C(38)-C(39)	1.397(8)
C(38)-H(38)	0.9300
C(39)-C(40)	1.339(9)
C(39)-H(39)	0.9300
C(40)-H(40)	0.9300
C(41)-C(42)	1.531(10)

C(42)-C(43)	1.534(10)
C(42)-H(42)	0.9800
C(43)-C(44)	1.511(11)
C(43)-H(43A)	0.9700
C(43)-H(43B)	0.9700
C(44)-C(46)	1.339(13)
C(44)-C(45)	1.491(11)
C(44)-H(44)	0.9800
C(45)-H(45A)	0.9600
C(45)-H(45B)	0.9600
C(45)-H(45C)	0.9600
C(46)-H(46A)	0.9600
C(46)-H(46B)	0.9600
C(46)-H(46C)	0.9600
C(47)-C(48)	1.406(9)
C(47)-H(47A)	0.9700
C(47)-H(47B)	0.9700
C(48)-H(48A)	0.9600
C(48)-H(48B)	0.9600
C(48)-H(48C)	0.9600
C(49)-C(50)	1.505(8)
C(50)-C(51)	1.517(6)
C(50)-H(50)	0.9800
C(51)-C(52')	1.511(10)
C(51)-C(52)	1.547(7)
C(51)-H(51A)	0.9700
C(51)-H(51B)	0.9700
C(51)-H(51C)	0.9700
C(51)-H(51D)	0.9700
C(52)-C(53)	1.523(8)
C(52)-C(54)	1.527(10)
C(52)-H(51C)	1.2097
C(52)-H(52)	0.9800
C(53)-H(53A)	0.9600
C(53)-H(53B)	0.9600
C(53)-H(53C)	0.9600
C(54)-H(54A)	0.9600
C(54)-H(54B)	0.9600

C(54)-H(54C)	0.9600
C(52')-C(53')	1.529(10)
C(52')-C(54')	1.539(11)
C(52')-H(52')	0.9800
C(53')-H(53D)	0.9600
C(53')-H(53E)	0.9600
C(53')-H(53F)	0.9600
C(54')-H(54D)	0.9600
C(54')-H(54E)	0.9600
C(54')-H(54F)	0.9600
C(55)-C(56)	1.452(8)
C(55)-H(55A)	0.9700
C(55)-H(55B)	0.9700
C(56)-H(56A)	0.9600
C(56)-H(56B)	0.9600
C(56)-H(56C)	0.9600
C(57)-C(58)	1.496(8)
C(58)-C(63)	1.368(8)
C(58)-C(59)	1.373(8)
C(59)-C(60)	1.382(8)
C(59)-H(59)	0.9300
C(60)-C(61)	1.382(8)
C(60)-H(60)	0.9300
C(61)-C(62)	1.383(8)
C(61)-C(71)	1.489(7)
C(62)-C(63)	1.399(8)
C(62)-H(62)	0.9300
C(63)-H(63)	0.9300
C(64)-C(65)	1.492(7)
C(65)-C(70)	1.377(8)
C(65)-C(66)	1.394(7)
C(66)-C(67)	1.381(7)
C(66)-H(66)	0.9300
C(67)-C(68)	1.377(8)
C(67)-H(67)	0.9300
C(68)-C(69)	1.377(8)
C(68)-C(77)	1.504(7)
C(69)-C(70)	1.399(8)

C(69)-H(69)	0.9300
C(70)-H(70)	0.9300
C(71)-C(72)	1.368(8)
C(71)-C(76)	1.417(7)
C(72)-C(73)	1.389(8)
C(72)-H(72)	0.9300
C(73)-C(74)	1.371(9)
C(73)-H(73)	0.9300
C(74)-C(75)	1.411(8)
C(74)-H(74)	0.9300
C(75)-C(80)	1.406(9)
C(75)-C(76)	1.435(7)
C(76)-C(77)	1.430(7)
C(77)-C(78)	1.381(8)
C(78)-C(79)	1.405(8)
C(78)-H(78)	0.9300
C(79)-C(80)	1.354(9)
C(79)-H(79)	0.9300
C(80)-H(80)	0.9300

C(17)-N(1)-C(2)	121.0(5)
C(17)-N(1)-H(1)	119.5
C(2)-N(1)-H(1)	119.5
C(24)-N(2)-C(10)	120.6(6)
C(24)-N(2)-H(2)	119.7
C(10)-N(2)-H(2)	119.7
C(57)-N(3)-C(42)	122.4(6)
C(57)-N(3)-H(3)	118.8
C(42)-N(3)-H(3)	118.8
C(64)-N(4)-C(50)	122.0(5)
C(64)-N(4)-H(4)	119.0
C(50)-N(4)-H(4)	119.0
C(1)-O(1)-C(7)	117.3(6)
C(9)-O(4)-C(15)	118.8(6)
C(41)-O(8)-C(47)	117.8(9)
C(49)-O(10)-C(55)	114.7(6)
O(2)-C(1)-O(1)	123.9(7)
O(2)-C(1)-C(2)	125.5(6)

O(1)-C(1)-C(2)	110.6(6)
N(1)-C(2)-C(1)	109.7(5)
N(1)-C(2)-C(3)	110.5(5)
C(1)-C(2)-C(3)	111.4(5)
N(1)-C(2)-H(2A)	108.4
C(1)-C(2)-H(2A)	108.4
C(3)-C(2)-H(2A)	108.4
C(4)-C(3)-C(2)	117.3(5)
C(4)-C(3)-H(3A)	108.0
C(2)-C(3)-H(3A)	108.0
C(4)-C(3)-H(3B)	108.0
C(2)-C(3)-H(3B)	108.0
H(3A)-C(3)-H(3B)	107.2
C(6)-C(4)-C(3)	113.3(6)
C(6)-C(4)-C(5)	111.4(7)
C(3)-C(4)-C(5)	110.1(6)
C(6)-C(4)-H(4A)	107.2
C(3)-C(4)-H(4A)	107.2
C(5)-C(4)-H(4A)	107.2
C(4)-C(5)-H(5A)	109.5
C(4)-C(5)-H(5B)	109.5
H(5A)-C(5)-H(5B)	109.5
C(4)-C(5)-H(5C)	109.5
H(5A)-C(5)-H(5C)	109.5
H(5B)-C(5)-H(5C)	109.5
C(4)-C(6)-H(6A)	109.5
C(4)-C(6)-H(6B)	109.5
H(6A)-C(6)-H(6B)	109.5
C(4)-C(6)-H(6C)	109.5
H(6A)-C(6)-H(6C)	109.5
H(6B)-C(6)-H(6C)	109.5
C(8)-C(7)-O(1)	107.8(8)
C(8)-C(7)-H(7A)	110.2
O(1)-C(7)-H(7A)	110.2
C(8)-C(7)-H(7B)	110.2
O(1)-C(7)-H(7B)	110.2
H(7A)-C(7)-H(7B)	108.5
C(7)-C(8)-H(8A)	109.5

C(7)-C(8)-H(8B)	109.5
H(8A)-C(8)-H(8B)	109.5
C(7)-C(8)-H(8C)	109.5
H(8A)-C(8)-H(8C)	109.5
H(8B)-C(8)-H(8C)	109.5
O(5)-C(9)-O(4)	123.0(8)
O(5)-C(9)-C(10)	124.8(8)
O(4)-C(9)-C(10)	112.1(6)
N(2)-C(10)-C(9)	109.6(6)
N(2)-C(10)-C(11)	112.6(6)
C(9)-C(10)-C(11)	110.9(6)
N(2)-C(10)-H(10)	107.9
C(9)-C(10)-H(10)	107.9
C(11)-C(10)-H(10)	107.9
C(12)-C(11)-C(10)	115.1(7)
C(12)-C(11)-H(11A)	108.5
C(10)-C(11)-H(11A)	108.5
C(12)-C(11)-H(11B)	108.5
C(10)-C(11)-H(11B)	108.5
H(11A)-C(11)-H(11B)	107.5
C(11)-C(12)-C(14)	118.8(9)
C(11)-C(12)-C(13)	109.4(8)
C(14)-C(12)-C(13)	104.4(8)
C(11)-C(12)-H(12)	107.9
C(14)-C(12)-H(12)	107.9
C(13)-C(12)-H(12)	107.9
C(12)-C(13)-H(13A)	109.5
C(12)-C(13)-H(13B)	109.5
H(13A)-C(13)-H(13B)	109.5
C(12)-C(13)-H(13C)	109.5
H(13A)-C(13)-H(13C)	109.5
H(13B)-C(13)-H(13C)	109.5
C(12)-C(14)-H(14A)	109.5
C(12)-C(14)-H(14B)	109.5
H(14A)-C(14)-H(14B)	109.5
C(12)-C(14)-H(14C)	109.5
H(14A)-C(14)-H(14C)	109.5
H(14B)-C(14)-H(14C)	109.5

O(4)-C(15)-C(16)	107.8(7)
O(4)-C(15)-H(15A)	110.2
C(16)-C(15)-H(15A)	110.2
O(4)-C(15)-H(15B)	110.2
C(16)-C(15)-H(15B)	110.2
H(15A)-C(15)-H(15B)	108.5
C(15)-C(16)-H(16A)	109.5
C(15)-C(16)-H(16B)	109.5
H(16A)-C(16)-H(16B)	109.5
C(15)-C(16)-H(16C)	109.5
H(16A)-C(16)-H(16C)	109.5
H(16B)-C(16)-H(16C)	109.5
O(3)-C(17)-N(1)	119.1(5)
O(3)-C(17)-C(18)	120.5(6)
N(1)-C(17)-C(18)	120.4(5)
C(23)-C(18)-C(19)	118.4(5)
C(23)-C(18)-C(17)	118.6(5)
C(19)-C(18)-C(17)	122.7(5)
C(20)-C(19)-C(18)	120.3(5)
C(20)-C(19)-H(19)	119.9
C(18)-C(19)-H(19)	119.9
C(21)-C(20)-C(19)	120.8(6)
C(21)-C(20)-H(20)	119.6
C(19)-C(20)-H(20)	119.6
C(20)-C(21)-C(22)	118.9(5)
C(20)-C(21)-C(31)	120.2(5)
C(22)-C(21)-C(31)	120.3(5)
C(23)-C(22)-C(21)	120.7(5)
C(23)-C(22)-H(22)	119.6
C(21)-C(22)-H(22)	119.6
C(22)-C(23)-C(18)	120.8(5)
C(22)-C(23)-H(23)	119.6
C(18)-C(23)-H(23)	119.6
O(6)-C(24)-N(2)	121.8(6)
O(6)-C(24)-C(25)	121.7(6)
N(2)-C(24)-C(25)	116.5(6)
C(26)-C(25)-C(30)	117.4(5)
C(26)-C(25)-C(24)	124.4(6)

C(30)-C(25)-C(24)	118.0(5)
C(25)-C(26)-C(27)	121.3(6)
C(25)-C(26)-H(26)	119.4
C(27)-C(26)-H(26)	119.4
C(28)-C(27)-C(26)	121.3(6)
C(28)-C(27)-H(27)	119.3
C(26)-C(27)-H(27)	119.3
C(27)-C(28)-C(29)	117.9(5)
C(27)-C(28)-C(37)	119.7(5)
C(29)-C(28)-C(37)	121.7(5)
C(30)-C(29)-C(28)	120.3(6)
C(30)-C(29)-H(29)	119.9
C(28)-C(29)-H(29)	119.9
C(29)-C(30)-C(25)	121.7(6)
C(29)-C(30)-H(30)	119.1
C(25)-C(30)-H(30)	119.1
C(32)-C(31)-C(36)	119.4(5)
C(32)-C(31)-C(21)	115.0(5)
C(36)-C(31)-C(21)	124.8(5)
C(31)-C(32)-C(33)	122.5(6)
C(31)-C(32)-H(32)	118.7
C(33)-C(32)-H(32)	118.7
C(34)-C(33)-C(32)	118.8(6)
C(34)-C(33)-H(33)	120.6
C(32)-C(33)-H(33)	120.6
C(33)-C(34)-C(35)	121.1(6)
C(33)-C(34)-H(34)	119.5
C(35)-C(34)-H(34)	119.5
C(34)-C(35)-C(40)	119.9(6)
C(34)-C(35)-C(36)	121.3(6)
C(40)-C(35)-C(36)	118.8(6)
C(37)-C(36)-C(31)	125.2(5)
C(37)-C(36)-C(35)	118.4(5)
C(31)-C(36)-C(35)	116.5(5)
C(38)-C(37)-C(36)	118.3(5)
C(38)-C(37)-C(28)	115.5(5)
C(36)-C(37)-C(28)	125.9(5)
C(37)-C(38)-C(39)	123.9(6)

C(37)-C(38)-H(38)	118.1
C(39)-C(38)-H(38)	118.1
C(40)-C(39)-C(38)	118.8(7)
C(40)-C(39)-H(39)	120.6
C(38)-C(39)-H(39)	120.6
C(39)-C(40)-C(35)	121.5(6)
C(39)-C(40)-H(40)	119.2
C(35)-C(40)-H(40)	119.2
O(7)-C(41)-O(8)	122.5(8)
O(7)-C(41)-C(42)	125.7(8)
O(8)-C(41)-C(42)	111.8(8)
N(3)-C(42)-C(41)	109.0(6)
N(3)-C(42)-C(43)	112.3(6)
C(41)-C(42)-C(43)	107.4(6)
N(3)-C(42)-H(42)	109.4
C(41)-C(42)-H(42)	109.4
C(43)-C(42)-H(42)	109.4
C(44)-C(43)-C(42)	116.0(8)
C(44)-C(43)-H(43A)	108.3
C(42)-C(43)-H(43A)	108.3
C(44)-C(43)-H(43B)	108.3
C(42)-C(43)-H(43B)	108.3
H(43A)-C(43)-H(43B)	107.4
C(46)-C(44)-C(45)	115.5(11)
C(46)-C(44)-C(43)	119.3(10)
C(45)-C(44)-C(43)	117.3(8)
C(46)-C(44)-H(44)	99.4
C(45)-C(44)-H(44)	99.4
C(43)-C(44)-H(44)	99.4
C(44)-C(45)-H(45A)	109.5
C(44)-C(45)-H(45B)	109.5
H(45A)-C(45)-H(45B)	109.5
C(44)-C(45)-H(45C)	109.5
H(45A)-C(45)-H(45C)	109.5
H(45B)-C(45)-H(45C)	109.5
C(44)-C(46)-H(46A)	109.5
C(44)-C(46)-H(46B)	109.5
H(46A)-C(46)-H(46B)	109.5

C(44)-C(46)-H(46C)	109.5
H(46A)-C(46)-H(46C)	109.5
H(46B)-C(46)-H(46C)	109.5
C(48)-C(47)-O(8)	100.2(12)
C(48)-C(47)-H(47A)	111.7
O(8)-C(47)-H(47A)	111.7
C(48)-C(47)-H(47B)	111.7
O(8)-C(47)-H(47B)	111.7
H(47A)-C(47)-H(47B)	109.5
C(47)-C(48)-H(48A)	109.5
C(47)-C(48)-H(48B)	109.5
H(48A)-C(48)-H(48B)	109.5
C(47)-C(48)-H(48C)	109.5
H(48A)-C(48)-H(48C)	109.5
H(48B)-C(48)-H(48C)	109.5
O(11)-C(49)-O(10)	121.3(7)
O(11)-C(49)-C(50)	125.3(6)
O(10)-C(49)-C(50)	113.4(5)
N(4)-C(50)-C(49)	108.0(5)
N(4)-C(50)-C(51)	113.2(4)
C(49)-C(50)-C(51)	117.4(5)
N(4)-C(50)-H(50)	105.8
C(49)-C(50)-H(50)	105.8
C(51)-C(50)-H(50)	105.8
C(52')-C(51)-C(50)	111.9(8)
C(52')-C(51)-C(52)	59.1(9)
C(50)-C(51)-C(52)	115.2(5)
C(52')-C(51)-H(51A)	139.1
C(50)-C(51)-H(51A)	108.5
C(52)-C(51)-H(51A)	108.5
C(52')-C(51)-H(51B)	53.1
C(50)-C(51)-H(51B)	108.5
C(52)-C(51)-H(51B)	108.5
H(51A)-C(51)-H(51B)	107.5
C(52')-C(51)-H(51C)	109.0
C(50)-C(51)-H(51C)	108.9
C(52)-C(51)-H(51C)	51.4
H(51A)-C(51)-H(51C)	62.4

H(51B)-C(51)-H(51C)	142.6
C(52')-C(51)-H(51D)	109.8
C(50)-C(51)-H(51D)	109.5
C(52)-C(51)-H(51D)	134.8
H(51A)-C(51)-H(51D)	48.3
H(51B)-C(51)-H(51D)	61.3
H(51C)-C(51)-H(51D)	107.8
C(53)-C(52)-C(54)	109.6(9)
C(53)-C(52)-C(51)	111.2(7)
C(54)-C(52)-C(51)	111.4(9)
C(53)-C(52)-H(51C)	94.3
C(54)-C(52)-H(51C)	148.8
C(51)-C(52)-H(51C)	38.8
C(53)-C(52)-H(52)	108.2
C(54)-C(52)-H(52)	108.2
C(51)-C(52)-H(52)	108.2
H(51C)-C(52)-H(52)	81.6
C(51)-C(52')-C(53')	107.8(12)
C(51)-C(52')-C(54')	109.9(14)
C(53')-C(52')-C(54')	111.3(15)
C(51)-C(52')-H(52')	109.3
C(53')-C(52')-H(52')	109.3
C(54')-C(52')-H(52')	109.3
C(52')-C(53')-H(53D)	109.5
C(52')-C(53')-H(53E)	109.5
H(53D)-C(53')-H(53E)	109.5
C(52')-C(53')-H(53F)	109.5
H(53D)-C(53')-H(53F)	109.5
H(53E)-C(53')-H(53F)	109.5
C(52')-C(54')-H(54D)	109.5
C(52')-C(54')-H(54E)	109.5
H(54D)-C(54')-H(54E)	109.5
C(52')-C(54')-H(54F)	109.5
H(54D)-C(54')-H(54F)	109.5
H(54E)-C(54')-H(54F)	109.5
O(10)-C(55)-C(56)	107.6(8)
O(10)-C(55)-H(55A)	110.2
C(56)-C(55)-H(55A)	110.2

O(10)-C(55)-H(55B)	110.2
C(56)-C(55)-H(55B)	110.2
H(55A)-C(55)-H(55B)	108.5
C(55)-C(56)-H(56A)	109.5
C(55)-C(56)-H(56B)	109.5
H(56A)-C(56)-H(56B)	109.5
C(55)-C(56)-H(56C)	109.5
H(56A)-C(56)-H(56C)	109.5
H(56B)-C(56)-H(56C)	109.5
O(9)-C(57)-N(3)	122.1(6)
O(9)-C(57)-C(58)	121.7(6)
N(3)-C(57)-C(58)	116.2(6)
C(63)-C(58)-C(59)	117.5(6)
C(63)-C(58)-C(57)	124.0(6)
C(59)-C(58)-C(57)	118.4(6)
C(58)-C(59)-C(60)	120.6(6)
C(58)-C(59)-H(59)	119.7
C(60)-C(59)-H(59)	119.7
C(59)-C(60)-C(61)	122.8(6)
C(59)-C(60)-H(60)	118.6
C(61)-C(60)-H(60)	118.6
C(62)-C(61)-C(60)	116.5(6)
C(62)-C(61)-C(71)	120.4(6)
C(60)-C(61)-C(71)	122.1(6)
C(61)-C(62)-C(63)	120.5(6)
C(61)-C(62)-H(62)	119.8
C(63)-C(62)-H(62)	119.8
C(58)-C(63)-C(62)	122.2(6)
C(58)-C(63)-H(63)	118.9
C(62)-C(63)-H(63)	118.9
O(12)-C(64)-N(4)	120.4(5)
O(12)-C(64)-C(65)	122.0(5)
N(4)-C(64)-C(65)	117.7(5)
C(70)-C(65)-C(66)	118.0(5)
C(70)-C(65)-C(64)	123.6(5)
C(66)-C(65)-C(64)	118.4(5)
C(67)-C(66)-C(65)	121.1(5)
C(67)-C(66)-H(66)	119.5

C(65)-C(66)-H(66)	119.5
C(68)-C(67)-C(66)	120.9(5)
C(68)-C(67)-H(67)	119.6
C(66)-C(67)-H(67)	119.6
C(67)-C(68)-C(69)	118.3(5)
C(67)-C(68)-C(77)	119.1(5)
C(69)-C(68)-C(77)	121.6(5)
C(68)-C(69)-C(70)	121.1(6)
C(68)-C(69)-H(69)	119.4
C(70)-C(69)-H(69)	119.4
C(65)-C(70)-C(69)	120.4(6)
C(65)-C(70)-H(70)	119.8
C(69)-C(70)-H(70)	119.8
C(72)-C(71)-C(76)	118.9(5)
C(72)-C(71)-C(61)	116.2(5)
C(76)-C(71)-C(61)	124.3(5)
C(71)-C(72)-C(73)	123.1(6)
C(71)-C(72)-H(72)	118.4
C(73)-C(72)-H(72)	118.4
C(74)-C(73)-C(72)	118.7(6)
C(74)-C(73)-H(73)	120.6
C(72)-C(73)-H(73)	120.6
C(73)-C(74)-C(75)	121.1(6)
C(73)-C(74)-H(74)	119.5
C(75)-C(74)-H(74)	119.5
C(80)-C(75)-C(74)	119.2(6)
C(80)-C(75)-C(76)	121.9(6)
C(74)-C(75)-C(76)	118.9(6)
C(71)-C(76)-C(77)	125.0(5)
C(71)-C(76)-C(75)	118.5(5)
C(77)-C(76)-C(75)	116.5(5)
C(78)-C(77)-C(76)	119.1(5)
C(78)-C(77)-C(68)	115.0(5)
C(76)-C(77)-C(68)	125.2(5)
C(77)-C(78)-C(79)	122.5(6)
C(77)-C(78)-H(78)	118.7
C(79)-C(78)-H(78)	118.7
C(80)-C(79)-C(78)	119.6(6)

C(80)-C(79)-H(79)	120.2
C(78)-C(79)-H(79)	120.2
C(79)-C(80)-C(75)	119.8(6)
C(79)-C(80)-H(80)	120.1
C(75)-C(80)-H(80)	120.1

Symmetry transformations used to generate equivalent atoms:

Table 4. Anisotropic displacement parameters ($Å^2$x 10^3) for 230519a. The anisotropic displacement factor exponent takes the form: $-2\pi^2[h^2 a^{*2}U^{11} + ... + 2 h k a^* b^* U^{12}]$

	U^{11}	U^{22}	U^{33}	U^{23}	U^{13}	U^{12}
N(1)	61(3)	39(3)	58(3)	-5(2)	-9(2)	-5(2)
N(2)	79(3)	51(3)	64(3)	0(3)	23(3)	2(3)
N(3)	95(4)	56(3)	50(3)	-9(3)	-4(3)	7(3)
N(4)	72(3)	39(3)	64(3)	7(2)	26(2)	0(3)
O(1)	85(3)	87(4)	102(4)	-13(3)	5(3)	-43(3)
O(2)	94(4)	135(5)	68(3)	-1(3)	22(3)	-22(4)
O(3)	88(3)	47(3)	83(3)	-10(2)	-15(2)	-5(2)
O(4)	71(3)	120(5)	112(4)	-11(4)	14(3)	-9(3)
O(5)	113(5)	195(8)	190(6)	-65(6)	88(5)	4(5)
O(6)	74(3)	55(3)	66(3)	12(2)	18(2)	12(2)
O(7)	122(5)	122(5)	106(4)	-1(4)	43(4)	-13(4)
O(8)	210(7)	221(9)	77(4)	-42(5)	66(4)	-87(7)
O(9)	97(3)	50(3)	65(3)	-2(2)	1(2)	4(2)
O(10)	81(3)	89(4)	80(3)	23(3)	10(2)	-22(3)
O(11)	72(3)	139(5)	71(3)	21(3)	4(2)	-15(3)
O(12)	67(2)	44(2)	56(2)	9(2)	15(2)	6(2)
C(1)	66(4)	70(4)	60(4)	1(3)	-3(3)	-9(3)
C(2)	56(3)	53(3)	60(3)	-9(3)	2(3)	-7(3)
C(3)	79(4)	62(4)	59(4)	-2(3)	4(3)	-13(3)
C(4)	75(4)	71(4)	67(4)	-3(4)	-3(3)	0(3)
C(5)	126(7)	111(7)	92(6)	27(5)	-21(5)	-6(6)
C(6)	146(8)	180(10)	86(6)	-48(6)	-26(5)	40(8)
C(7)	105(6)	126(7)	128(7)	1(6)	6(5)	-60(5)
C(8)	139(9)	226(13)	190(11)	-14(10)	-7(8)	-110(9)
C(9)	86(5)	90(5)	96(5)	-4(5)	32(4)	2(4)
C(10)	90(4)	63(4)	61(4)	5(3)	19(3)	10(4)
C(11)	116(5)	79(5)	82(5)	-12(4)	5(4)	15(5)
C(12)	148(7)	93(6)	116(6)	-25(5)	-10(6)	4(6)
C(13)	227(11)	126(9)	143(9)	-52(8)	-57(8)	-6(9)
C(14)	167(9)	104(7)	148(9)	29(7)	-33(7)	15(7)
C(15)	74(5)	120(7)	132(7)	4(6)	8(5)	10(5)
C(16)	88(6)	132(9)	160(9)	-22(7)	18(6)	-24(6)
C(17)	55(3)	45(4)	64(4)	-9(3)	10(3)	-8(3)

C(18)	47(3)	43(3)	47(3)	-3(3)	6(2)	-6(3)
C(19)	55(3)	41(3)	61(4)	3(3)	2(3)	0(3)
C(20)	62(4)	46(4)	65(4)	-11(3)	0(3)	-4(3)
C(21)	43(3)	46(3)	53(3)	-5(3)	6(2)	-4(3)
C(22)	53(3)	49(4)	56(3)	4(3)	5(3)	3(3)
C(23)	51(3)	40(3)	63(4)	-9(3)	9(3)	4(3)
C(24)	43(3)	59(4)	64(4)	-3(3)	6(3)	4(3)
C(25)	51(3)	52(4)	51(3)	1(3)	6(2)	4(3)
C(26)	84(4)	60(4)	62(4)	-1(3)	20(3)	9(4)
C(27)	75(4)	58(4)	71(4)	16(4)	16(3)	8(4)
C(28)	49(3)	47(4)	58(4)	-2(3)	4(3)	0(3)
C(29)	59(4)	57(4)	62(4)	-7(3)	15(3)	-2(3)
C(30)	68(4)	49(4)	56(4)	2(3)	8(3)	5(3)
C(31)	54(3)	43(3)	58(3)	2(3)	9(3)	-4(3)
C(32)	67(4)	63(4)	71(4)	-7(4)	15(3)	-8(3)
C(33)	98(6)	74(5)	62(4)	-13(4)	21(4)	-13(4)
C(34)	100(5)	66(5)	52(4)	-4(3)	12(4)	-12(4)
C(35)	74(4)	50(4)	61(4)	9(3)	0(3)	-15(3)
C(36)	60(3)	49(4)	49(3)	2(3)	-3(3)	-5(3)
C(37)	53(3)	54(4)	54(3)	7(3)	0(3)	-5(3)
C(38)	61(4)	101(6)	73(4)	18(4)	5(3)	-6(4)
C(39)	71(4)	103(6)	71(5)	22(5)	-8(4)	-8(4)
C(40)	96(5)	70(5)	57(4)	10(4)	-15(4)	-22(4)
C(41)	106(6)	107(6)	61(4)	0(5)	16(4)	-6(5)
C(42)	100(5)	88(5)	56(4)	-7(4)	9(3)	-15(4)
C(43)	89(5)	140(7)	69(4)	-3(5)	7(4)	-23(5)
C(44)	166(8)	183(9)	102(6)	-34(7)	31(6)	-85(7)
C(45)	132(7)	191(11)	109(7)	12(7)	50(6)	-30(8)
C(46)	254(13)	164(12)	186(10)	-41(10)	55(10)	-80(10)
C(47)	182(10)	212(11)	155(9)	37(9)	37(8)	-5(9)
C(48)	204(14)	350(20)	289(17)	130(16)	42(13)	3(15)
C(49)	53(3)	76(4)	66(4)	9(4)	21(3)	5(3)
C(50)	64(3)	59(4)	59(3)	1(3)	23(3)	9(3)
C(51)	76(4)	80(5)	64(4)	-5(4)	15(3)	-14(4)
C(52)	84(5)	73(6)	66(5)	-14(5)	17(4)	5(5)
C(53)	157(11)	154(12)	77(7)	-8(8)	4(7)	14(10)
C(54)	193(10)	116(8)	127(9)	-47(7)	46(8)	-9(8)
C(52')	84(5)	73(6)	66(5)	-14(5)	17(4)	5(5)

C(53')	157(11)	154(12)	77(7)	-8(8)	4(7)	14(10)
C(54')	193(10)	116(8)	127(9)	-47(7)	46(8)	-9(8)
C(55)	85(5)	132(7)	132(7)	20(6)	19(5)	-39(5)
C(56)	144(9)	217(13)	222(12)	90(11)	-4(8)	-87(9)
C(57)	61(4)	56(4)	56(4)	3(3)	15(3)	-1(3)
C(58)	56(3)	45(4)	53(3)	-3(3)	4(3)	-3(3)
C(59)	71(4)	52(4)	63(4)	-7(3)	7(3)	-5(3)
C(60)	68(4)	60(4)	58(4)	0(3)	1(3)	-4(3)
C(61)	47(3)	57(4)	56(3)	0(3)	8(3)	-10(3)
C(62)	68(4)	54(4)	75(4)	-10(4)	4(3)	-7(3)
C(63)	68(4)	59(4)	71(4)	3(4)	-1(3)	2(3)
C(64)	44(3)	40(3)	52(3)	-2(3)	9(2)	5(2)
C(65)	41(3)	39(3)	56(3)	4(3)	1(2)	-1(2)
C(66)	62(4)	37(3)	54(3)	8(3)	11(3)	14(3)
C(67)	54(3)	50(4)	57(3)	7(3)	16(3)	12(3)
C(68)	48(3)	47(3)	59(4)	2(3)	14(3)	1(3)
C(69)	88(5)	43(4)	74(4)	2(3)	32(4)	-8(3)
C(70)	80(4)	50(4)	69(4)	-8(3)	31(3)	-5(3)
C(71)	54(3)	56(4)	55(3)	0(3)	16(3)	-13(3)
C(72)	48(3)	99(6)	80(5)	-17(4)	18(3)	-23(4)
C(73)	64(4)	128(7)	74(5)	-30(5)	31(4)	-20(5)
C(74)	75(4)	87(5)	68(4)	-20(4)	29(4)	-24(4)
C(75)	68(4)	57(4)	57(4)	-13(3)	16(3)	-22(3)
C(76)	57(3)	55(4)	50(3)	-2(3)	19(3)	-16(3)
C(77)	58(3)	41(3)	59(4)	9(3)	9(3)	-5(3)
C(78)	70(4)	66(5)	72(4)	8(4)	17(3)	10(3)
C(79)	80(5)	78(5)	61(4)	14(4)	-3(3)	-5(4)
C(80)	90(5)	63(4)	59(4)	-5(4)	15(4)	-20(4)

Table 5. Hydrogen coordinates (x 10^4) and isotropic displacement parameters ($Å^2$x 10^3) for 230519a.

	x	y	z	U(eq)
H(1)	7938	1643	7540	65
H(2)	3394	2797	7858	76
H(3)	6823	2839	2188	83
H(4)	11795	1560	2654	68
H(2A)	8411	3599	8298	69
H(3A)	8497	1308	8580	81
H(3B)	9226	1065	8216	81
H(4A)	10140	2439	8830	87
H(5A)	9534	377	9481	170
H(5B)	10484	860	9512	170
H(5C)	10043	38	8981	170
H(6A)	8839	2645	9502	213
H(6B)	9210	3854	9186	213
H(6C)	9782	3130	9697	213
H(7A)	10888	4465	7885	145
H(7B)	10414	5866	7773	145
H(8A)	11193	4921	8817	283
H(8B)	10658	6265	8736	283
H(8C)	11509	6139	8478	283
H(10)	3236	623	8554	85
H(11A)	3128	3406	8795	112
H(11B)	2770	2346	9189	112
H(12)	4449	2609	9024	147
H(13A)	3710	3092	10028	261
H(13B)	4017	4243	9650	261
H(13C)	4686	3263	9996	261
H(14A)	4383	351	9230	219
H(14B)	3854	633	9725	219
H(14C)	4816	1072	9790	219
H(15A)	853	299	7134	132
H(15B)	572	1063	7658	132
H(16A)	945	-920	8220	190

H(16B)	1107	-1670	7663	190
H(16C)	183	-1183	7726	190
H(19)	7316	999	6728	64
H(20)	6481	483	5866	70
H(22)	5437	4178	5819	64
H(23)	6301	4736	6656	62
H(26)	3467	3438	7055	82
H(27)	3576	3975	6127	81
H(29)	4485	262	5896	71
H(30)	4357	-273	6823	69
H(32)	6572	1536	4898	79
H(33)	6111	1504	3915	93
H(34)	4745	2177	3569	87
H(38)	2724	3040	5263	94
H(39)	2320	3129	4286	101
H(40)	3297	2684	3704	93
H(42)	6487	625	1493	98
H(43A)	5449	2965	1530	119
H(43B)	5283	1933	1024	119
H(44)	4391	1768	1671	179
H(45A)	5544	737	2534	212
H(45B)	4566	988	2534	212
H(45C)	5182	2221	2499	212
H(46A)	5020	-666	1607	298
H(46B)	4413	116	1130	298
H(46C)	4105	-224	1712	298
H(47A)	7810	2505	342	218
H(47B)	7488	1170	2	218
H(48A)	6630	3614	-46	423
H(48B)	6244	2285	-346	423
H(48C)	7040	2975	-546	423
H(50)	12654	1740	2027	71
H(51A)	11726	3904	1496	88
H(51B)	11284	2483	1498	88
H(51C)	12167	3479	1288	88
H(51D)	11341	3467	1577	88
H(52)	12826	2796	1039	89
H(53A)	11232	2785	323	197

H(53B)	12119	3158	148	197
H(53C)	11680	4156	529	197
H(54A)	12515	738	596	214
H(54B)	11608	698	785	214
H(54C)	12431	536	1241	214
H(52')	11284	1039	1432	89
H(53D)	10606	1276	495	197
H(53E)	11120	2606	416	197
H(53F)	10424	2604	821	197
H(54D)	12493	219	1222	214
H(54E)	12761	1608	979	214
H(54F)	12134	638	592	214
H(55A)	14500	4808	2138	139
H(55B)	13924	5771	2449	139
H(56A)	13434	6707	1530	296
H(56B)	14205	5952	1326	296
H(56C)	14374	7082	1796	296
H(59)	8191	-92	3318	75
H(60)	8748	654	4213	76
H(62)	7909	4390	3812	80
H(63)	7324	3613	2917	81
H(66)	10815	5014	3468	61
H(67)	10247	4608	4288	64
H(69)	10503	630	4072	80
H(70)	11066	1027	3240	77
H(72)	7465	3846	4739	90
H(73)	7542	4102	5712	104
H(74)	8725	3300	6308	90
H(78)	11348	1631	5128	82
H(79)	11357	1724	6107	89
H(80)	10193	2556	6460	84

Table 6. Torsion angles [°] for 230519a.

C(7)-O(1)-C(1)-O(2)	-6.3(11)
C(7)-O(1)-C(1)-C(2)	171.8(6)
C(17)-N(1)-C(2)-C(1)	-70.0(7)
C(17)-N(1)-C(2)-C(3)	166.8(5)
O(2)-C(1)-C(2)-N(1)	-30.8(9)
O(1)-C(1)-C(2)-N(1)	151.1(5)
O(2)-C(1)-C(2)-C(3)	91.9(8)
O(1)-C(1)-C(2)-C(3)	-86.2(7)
N(1)-C(2)-C(3)-C(4)	-177.4(5)
C(1)-C(2)-C(3)-C(4)	60.3(7)
C(2)-C(3)-C(4)-C(6)	59.9(9)
C(2)-C(3)-C(4)-C(5)	-174.6(6)
C(1)-O(1)-C(7)-C(8)	-158.0(9)
C(15)-O(4)-C(9)-O(5)	-5.4(13)
C(15)-O(4)-C(9)-C(10)	173.8(7)
C(24)-N(2)-C(10)-C(9)	-93.5(7)
C(24)-N(2)-C(10)-C(11)	142.6(6)
O(5)-C(9)-C(10)-N(2)	-139.4(9)
O(4)-C(9)-C(10)-N(2)	41.4(9)
O(5)-C(9)-C(10)-C(11)	-14.5(12)
O(4)-C(9)-C(10)-C(11)	166.3(6)
N(2)-C(10)-C(11)-C(12)	-79.0(9)
C(9)-C(10)-C(11)-C(12)	157.8(8)
C(10)-C(11)-C(12)-C(14)	-56.9(12)
C(10)-C(11)-C(12)-C(13)	-176.6(8)
C(9)-O(4)-C(15)-C(16)	-82.6(10)
C(2)-N(1)-C(17)-O(3)	-6.3(9)
C(2)-N(1)-C(17)-C(18)	173.2(5)
O(3)-C(17)-C(18)-C(23)	-8.7(8)
N(1)-C(17)-C(18)-C(23)	171.8(5)
O(3)-C(17)-C(18)-C(19)	166.1(6)
N(1)-C(17)-C(18)-C(19)	-13.4(8)
C(23)-C(18)-C(19)-C(20)	2.8(8)
C(17)-C(18)-C(19)-C(20)	-172.0(5)
C(18)-C(19)-C(20)-C(21)	-1.0(9)
C(19)-C(20)-C(21)-C(22)	-2.2(9)

C(19)-C(20)-C(21)-C(31)	169.5(5)
C(20)-C(21)-C(22)-C(23)	3.5(8)
C(31)-C(21)-C(22)-C(23)	-168.1(5)
C(21)-C(22)-C(23)-C(18)	-1.8(8)
C(19)-C(18)-C(23)-C(22)	-1.4(8)
C(17)-C(18)-C(23)-C(22)	173.6(5)
C(10)-N(2)-C(24)-O(6)	-13.2(9)
C(10)-N(2)-C(24)-C(25)	164.8(5)
O(6)-C(24)-C(25)-C(26)	173.3(6)
N(2)-C(24)-C(25)-C(26)	-4.8(9)
O(6)-C(24)-C(25)-C(30)	-1.0(8)
N(2)-C(24)-C(25)-C(30)	-179.0(5)
C(30)-C(25)-C(26)-C(27)	2.9(9)
C(24)-C(25)-C(26)-C(27)	-171.4(6)
C(25)-C(26)-C(27)-C(28)	-0.8(10)
C(26)-C(27)-C(28)-C(29)	-1.8(9)
C(26)-C(27)-C(28)-C(37)	168.2(6)
C(27)-C(28)-C(29)-C(30)	2.1(9)
C(37)-C(28)-C(29)-C(30)	-167.6(5)
C(28)-C(29)-C(30)-C(25)	0.0(9)
C(26)-C(25)-C(30)-C(29)	-2.5(9)
C(24)-C(25)-C(30)-C(29)	172.2(5)
C(20)-C(21)-C(31)-C(32)	-54.9(8)
C(22)-C(21)-C(31)-C(32)	116.7(6)
C(20)-C(21)-C(31)-C(36)	135.3(6)
C(22)-C(21)-C(31)-C(36)	-53.2(8)
C(36)-C(31)-C(32)-C(33)	3.5(10)
C(21)-C(31)-C(32)-C(33)	-167.0(6)
C(31)-C(32)-C(33)-C(34)	2.6(11)
C(32)-C(33)-C(34)-C(35)	-6.1(11)
C(33)-C(34)-C(35)-C(40)	-178.1(7)
C(33)-C(34)-C(35)-C(36)	3.5(10)
C(32)-C(31)-C(36)-C(37)	174.1(6)
C(21)-C(31)-C(36)-C(37)	-16.5(9)
C(32)-C(31)-C(36)-C(35)	-5.8(8)
C(21)-C(31)-C(36)-C(35)	163.6(5)
C(34)-C(35)-C(36)-C(37)	-177.4(6)
C(40)-C(35)-C(36)-C(37)	4.2(9)

C(34)-C(35)-C(36)-C(31)	2.5(9)
C(40)-C(35)-C(36)-C(31)	-175.9(5)
C(31)-C(36)-C(37)-C(38)	173.0(6)
C(35)-C(36)-C(37)-C(38)	-7.1(9)
C(31)-C(36)-C(37)-C(28)	-13.1(10)
C(35)-C(36)-C(37)-C(28)	166.8(6)
C(27)-C(28)-C(37)-C(38)	-51.2(8)
C(29)-C(28)-C(37)-C(38)	118.4(7)
C(27)-C(28)-C(37)-C(36)	134.8(7)
C(29)-C(28)-C(37)-C(36)	-55.6(8)
C(36)-C(37)-C(38)-C(39)	6.3(11)
C(28)-C(37)-C(38)-C(39)	-168.2(7)
C(37)-C(38)-C(39)-C(40)	-2.3(12)
C(38)-C(39)-C(40)-C(35)	-0.9(11)
C(34)-C(35)-C(40)-C(39)	-178.6(7)
C(36)-C(35)-C(40)-C(39)	-0.2(10)
C(47)-O(8)-C(41)-O(7)	16.2(16)
C(47)-O(8)-C(41)-C(42)	-161.3(9)
C(57)-N(3)-C(42)-C(41)	-114.7(7)
C(57)-N(3)-C(42)-C(43)	126.4(7)
O(7)-C(41)-C(42)-N(3)	-18.4(13)
O(8)-C(41)-C(42)-N(3)	158.9(8)
O(7)-C(41)-C(42)-C(43)	103.5(10)
O(8)-C(41)-C(42)-C(43)	-79.2(10)
N(3)-C(42)-C(43)-C(44)	-72.2(10)
C(41)-C(42)-C(43)-C(44)	167.9(8)
C(42)-C(43)-C(44)-C(46)	-84.3(14)
C(42)-C(43)-C(44)-C(45)	63.5(14)
C(41)-O(8)-C(47)-C(48)	-107.2(14)
C(55)-O(10)-C(49)-O(11)	3.1(10)
C(55)-O(10)-C(49)-C(50)	-174.6(6)
C(64)-N(4)-C(50)-C(49)	55.8(7)
C(64)-N(4)-C(50)-C(51)	-75.9(7)
O(11)-C(49)-C(50)-N(4)	40.8(9)
O(10)-C(49)-C(50)-N(4)	-141.6(5)
O(11)-C(49)-C(50)-C(51)	170.1(6)
O(10)-C(49)-C(50)-C(51)	-12.2(8)
N(4)-C(50)-C(51)-C(52')	-86.6(10)

C(49)-C(50)-C(51)-C(52')	146.5(9)
N(4)-C(50)-C(51)-C(52)	-151.7(6)
C(49)-C(50)-C(51)-C(52)	81.5(8)
C(52')-C(51)-C(52)-C(53)	94.5(11)
C(50)-C(51)-C(52)-C(53)	-164.0(8)
C(52')-C(51)-C(52)-C(54)	-28.1(13)
C(50)-C(51)-C(52)-C(54)	73.3(11)
C(50)-C(51)-C(52')-C(53')	168.7(14)
C(52)-C(51)-C(52')-C(53')	-84.1(17)
C(50)-C(51)-C(52')-C(54')	-70(2)
C(52)-C(51)-C(52')-C(54')	37(2)
C(49)-O(10)-C(55)-C(56)	-172.6(8)
C(42)-N(3)-C(57)-O(9)	-2.3(10)
C(42)-N(3)-C(57)-C(58)	-179.5(5)
O(9)-C(57)-C(58)-C(63)	-176.2(6)
N(3)-C(57)-C(58)-C(63)	1.0(9)
O(9)-C(57)-C(58)-C(59)	1.2(9)
N(3)-C(57)-C(58)-C(59)	178.4(5)
C(63)-C(58)-C(59)-C(60)	0.9(9)
C(57)-C(58)-C(59)-C(60)	-176.6(5)
C(58)-C(59)-C(60)-C(61)	-0.6(10)
C(59)-C(60)-C(61)-C(62)	-0.4(9)
C(59)-C(60)-C(61)-C(71)	168.3(5)
C(60)-C(61)-C(62)-C(63)	1.2(9)
C(71)-C(61)-C(62)-C(63)	-167.8(5)
C(59)-C(58)-C(63)-C(62)	-0.2(9)
C(57)-C(58)-C(63)-C(62)	177.2(5)
C(61)-C(62)-C(63)-C(58)	-0.9(10)
C(50)-N(4)-C(64)-O(12)	2.7(8)
C(50)-N(4)-C(64)-C(65)	-176.0(4)
O(12)-C(64)-C(65)-C(70)	175.6(6)
N(4)-C(64)-C(65)-C(70)	-5.7(8)
O(12)-C(64)-C(65)-C(66)	-6.0(8)
N(4)-C(64)-C(65)-C(66)	172.7(5)
C(70)-C(65)-C(66)-C(67)	5.1(8)
C(64)-C(65)-C(66)-C(67)	-173.4(5)
C(65)-C(66)-C(67)-C(68)	-0.8(9)
C(66)-C(67)-C(68)-C(69)	-3.2(9)

C(66)-C(67)-C(68)-C(77)	165.4(5)
C(67)-C(68)-C(69)-C(70)	2.9(9)
C(77)-C(68)-C(69)-C(70)	-165.4(6)
C(66)-C(65)-C(70)-C(69)	-5.4(9)
C(64)-C(65)-C(70)-C(69)	173.0(5)
C(68)-C(69)-C(70)-C(65)	1.4(10)
C(62)-C(61)-C(71)-C(72)	50.3(8)
C(60)-C(61)-C(71)-C(72)	-118.0(7)
C(62)-C(61)-C(71)-C(76)	-139.2(6)
C(60)-C(61)-C(71)-C(76)	52.5(8)
C(76)-C(71)-C(72)-C(73)	-6.6(10)
C(61)-C(71)-C(72)-C(73)	164.4(6)
C(71)-C(72)-C(73)-C(74)	-1.1(12)
C(72)-C(73)-C(74)-C(75)	6.0(11)
C(73)-C(74)-C(75)-C(80)	175.7(7)
C(73)-C(74)-C(75)-C(76)	-3.0(10)
C(72)-C(71)-C(76)-C(77)	-169.9(6)
C(61)-C(71)-C(76)-C(77)	19.8(9)
C(72)-C(71)-C(76)-C(75)	9.3(9)
C(61)-C(71)-C(76)-C(75)	-160.9(5)
C(80)-C(75)-C(76)-C(71)	176.6(6)
C(74)-C(75)-C(76)-C(71)	-4.7(8)
C(80)-C(75)-C(76)-C(77)	-4.1(9)
C(74)-C(75)-C(76)-C(77)	174.6(6)
C(71)-C(76)-C(77)-C(78)	-172.8(6)
C(75)-C(76)-C(77)-C(78)	8.0(8)
C(71)-C(76)-C(77)-C(68)	16.7(9)
C(75)-C(76)-C(77)-C(68)	-162.6(5)
C(67)-C(68)-C(77)-C(78)	-103.4(7)
C(69)-C(68)-C(77)-C(78)	64.8(8)
C(67)-C(68)-C(77)-C(76)	67.5(8)
C(69)-C(68)-C(77)-C(76)	-124.3(7)
C(76)-C(77)-C(78)-C(79)	-6.4(10)
C(68)-C(77)-C(78)-C(79)	165.1(6)
C(77)-C(78)-C(79)-C(80)	0.3(11)
C(78)-C(79)-C(80)-C(75)	3.9(11)
C(74)-C(75)-C(80)-C(79)	179.5(6)
C(76)-C(75)-C(80)-C(79)	-1.9(10)

Symmetry transformations used to generate equivalent atoms:

Table 7. Hydrogen bonds for 230519a [Å and °].

D-H	d(D-H)	d(H..A)	<DHA	d(D..A)	A
N1-H1	0.860	2.240	152.15	3.027	O12 [-x+2, y-1/2, -z+1]
N2-H2	0.860	2.405	149.33	3.175	O9 [-x+1, y+1/2, -z+1]
N3-H3	0.860	2.236	154.59	3.035	O6 [-x+1, y+1/2, -z+1]
N4-H4	0.860	2.179	135.79	2.859	O3 [-x+2, y-1/2, -z+1]

----- Write up for **11**

Table 1. Crystal data and structure refinement for 220723f.

Identification code	*220723f*
Empirical formula	*C43 H48 N2 O4 S*
Formula weight	*688.89*
Temperature	*293(2) K*
Wavelength	*0.71073 A*
Crystal system, space group	*Orthorhombic, P2(1)2(1)2(1)*
Unit cell dimensions	*a = 8.9352(8) A alpha = 90 deg.*
	b = 16.4641(15) A beta = 90 deg.
	c = 25.813(2) A gamma = 90 deg.
Volume	*3797.4(6) A^3*
Z, Calculated density	*4, 1.205 Mg/m^3*
Absorption coefficient	*0.129 mm^-1*
F(000)	*1472*

Crystal size ***0.32 x 0.18 x 0.10 mm***

Theta range for data collection ***2.00 to 25.02 deg.***

Limiting indices ***-10<=h<=10, -19<=k<=17, -30<=l<=26***

Reflections collected / unique ***18630 / 6656 [R(int) = 0.0815]***

Completeness to theta = 25.02 ***99.9 %***

Absorption correction ***Semi-empirical from equivalents***

Max. and min. transmission ***0.9872 and 0.9599***

Refinement method ***Full-matrix least-squares on F^2***

Data / restraints / parameters ***6656 / 0 / 459***

Goodness-of-fit on F^2 ***1.042***

Final R indices [I>2sigma(I)] ***R1 = 0.0594, wR2 = 0.1094***

R indices (all data) ***R1 = 0.1229, wR2 = 0.1252***

Absolute structure parameter ***-0.08(11)***

Extinction coefficient ***0.0049(5)***

Largest diff. peak and hole ***0.489 and -0.269 e.A^-3***

Table 2. Atomic coordinates (x 10^4) and equivalent isotropic displacement parameters (A^2 x 10^3) for 220723f. U(eq) is defined as one third of the trace of the orthogonalized Uij tensor.

	x	y	z	U(eq)
N(1)	4466(5)	6950(2)	6738(1)	82(1)
N(2)	-2908(3)	4059(2)	6553(1)	48(1)
O(1)	5038(4)	5641(2)	6589(1)	80(1)
O(2)	3121(8)	7988(3)	6121(2)	180(3)
O(3)	3959(6)	7375(3)	5455(2)	167(2)
O(4)	-4032(3)	3385(2)	7384(1)	75(1)
S(1)	-3494(1)	3227(1)	6853(1)	56(1)
C(1)	3811(16)	7573(5)	5914(3)	188(7)
C(2)	5167(7)	7236(3)	6277(2)	84(2)
C(3)	6449(7)	7812(3)	6377(2)	88(2)
C(4)	7071(7)	8159(3)	5875(2)	108(2)
C(5)	7625(7)	7388(3)	6694(2)	104(2)
C(6)	1792(8)	8275(4)	5779(3)	146(3)
C(7)	4458(5)	6161(3)	6863(2)	63(1)
C(8)	3730(5)	5949(2)	7360(2)	53(1)
C(9)	2575(5)	6404(2)	7573(2)	55(1)
C(10)	2003(5)	6207(2)	8051(2)	56(1)
C(11)	2575(5)	5557(2)	8329(2)	50(1)
C(12)	3691(5)	5097(3)	8110(2)	61(1)
C(13)	4269(5)	5293(3)	7629(2)	61(1)
C(14)	2129(6)	5413(3)	8875(1)	57(1)
C(15)	3278(6)	5368(3)	9218(2)	76(1)
C(16)	3028(9)	5338(3)	9750(2)	102(2)
C(17)	1622(9)	5392(3)	9936(2)	96(2)
C(18)	406(7)	5438(3)	9605(2)	71(2)
C(19)	638(6)	5386(3)	9059(2)	58(1)
C(20)	-678(6)	5351(3)	8744(2)	57(1)
C(21)	-2038(6)	5472(3)	8967(2)	73(2)
C(22)	-2209(8)	5566(3)	9500(2)	93(2)
C(23)	-1013(8)	5540(3)	9810(2)	88(2)
C(24)	-679(5)	5109(2)	8192(2)	50(1)

C(25)	***15(5)***	***4421(2)***	***8026(1)***	***56(1)***
C(26)	***-142(5)***	***4130(2)***	***7523(2)***	***54(1)***
C(27)	***-1008(4)***	***4551(2)***	***7171(1)***	***44(1)***
C(28)	***-1677(5)***	***5266(2)***	***7337(1)***	***51(1)***
C(29)	***-1523(5)***	***5538(2)***	***7831(2)***	***56(1)***
C(30)	***-1310(5)***	***4272(2)***	***6618(1)***	***46(1)***
C(31)	***-366(5)***	***3545(2)***	***6459(1)***	***56(1)***
C(32)	***-530(6)***	***3311(3)***	***5890(2)***	***83(2)***
C(33)	***298(7)***	***2599(3)***	***5733(2)***	***105(2)***
C(34)	***-930(5)***	***4968(2)***	***6255(2)***	***52(1)***
C(35)	***480(6)***	***5305(3)***	***6283(2)***	***71(1)***
C(36)	***915(7)***	***5896(4)***	***5948(2)***	***92(2)***
C(37)	***-79(10)***	***6175(3)***	***5576(2)***	***97(2)***
C(38)	***-1438(8)***	***5867(3)***	***5556(2)***	***85(2)***
C(39)	***-1890(6)***	***5261(3)***	***5891(2)***	***67(1)***
C(40)	***-5175(5)***	***3047(3)***	***6469(2)***	***57(1)***
C(41)	***-6119(5)***	***3780(3)***	***6429(2)***	***89(2)***
C(42)	***-4702(6)***	***2781(3)***	***5937(2)***	***89(2)***
C(43)	***-5962(6)***	***2356(3)***	***6742(2)***	***97(2)***

Table 3. Bond lengths [A] and angles [deg] for 220723f.

N(1)-C(7)	*1.339(5)*
N(1)-C(2)	*1.425(5)*
N(1)-H(1)	*0.8600*
N(2)-C(30)	*1.479(5)*
N(2)-S(1)	*1.659(3)*
N(2)-H(2)	*0.9000*
O(1)-C(7)	*1.226(5)*
O(2)-C(1)	*1.064(10)*
O(2)-C(6)	*1.555(9)*
O(3)-C(1)	*1.236(6)*
O(4)-S(1)	*1.478(3)*
S(1)-C(40)	*1.824(4)*
C(1)-C(2)	*1.628(13)*
C(2)-C(3)	*1.510(7)*
C(2)-H(2A)	*0.9800*
C(3)-C(5)	*1.505(7)*
C(3)-C(4)	*1.521(6)*
C(3)-H(3)	*0.9800*
C(4)-H(4A)	*0.9600*
C(4)-H(4B)	*0.9600*
C(4)-H(4C)	*0.9600*
C(5)-H(5A)	*0.9600*
C(5)-H(5B)	*0.9600*
C(5)-H(5C)	*0.9600*
C(6)-H(6A)	*0.9600*
C(6)-H(6B)	*0.9600*
C(6)-H(6C)	*0.9600*
C(7)-C(8)	*1.480(5)*
C(8)-C(13)	*1.371(5)*
C(8)-C(9)	*1.389(5)*
C(9)-C(10)	*1.376(5)*
C(9)-H(9)	*0.9300*
C(10)-C(11)	*1.385(5)*
C(10)-H(10)	*0.9300*
C(11)-C(12)	*1.374(5)*
C(11)-C(14)	*1.484(5)*
C(12)-C(13)	*1.381(5)*
C(12)-H(12)	*0.9300*
C(13)-H(13)	*0.9300*

C(14)-C(15)	*1.359(6)*
C(14)-C(19)	*1.416(6)*
C(15)-C(16)	*1.393(6)*
C(15)-H(15)	*0.9300*
C(16)-C(17)	*1.348(7)*
C(16)-H(16)	*0.9300*
C(17)-C(18)	*1.385(7)*
C(17)-H(17)	*0.9300*
C(18)-C(23)	*1.384(7)*
C(18)-C(19)	*1.428(6)*
C(19)-C(20)	*1.431(6)*
C(20)-C(21)	*1.359(6)*
C(20)-C(24)	*1.481(5)*
C(21)-C(22)	*1.393(6)*
C(21)-H(21)	*0.9300*
C(22)-C(23)	*1.336(7)*
C(22)-H(22)	*0.9300*
C(23)-H(23)	*0.9300*
C(24)-C(25)	*1.360(5)*
C(24)-C(29)	*1.391(5)*
C(25)-C(26)	*1.391(5)*
C(25)-H(25)	*0.9300*
C(26)-C(27)	*1.380(5)*
C(26)-H(26)	*0.9300*
C(27)-C(28)	*1.387(5)*
C(27)-C(30)	*1.524(5)*
C(28)-C(29)	*1.359(5)*
C(28)-H(28)	*0.9300*
C(29)-H(29)	*0.9300*
C(30)-C(34)	*1.519(5)*
C(30)-C(31)	*1.521(5)*
C(31)-C(32)	*1.526(5)*
C(31)-H(31A)	*0.9700*
C(31)-H(31B)	*0.9700*
C(32)-C(33)	*1.443(6)*
C(32)-H(32A)	*0.9700*
C(32)-H(32B)	*0.9700*
C(33)-H(33A)	*0.9600*
C(33)-H(33B)	*0.9600*
C(33)-H(33C)	*0.9600*
C(34)-C(39)	*1.361(6)*
C(34)-C(35)	*1.378(6)*
C(35)-C(36)	*1.360(6)*
C(35)-H(35)	*0.9300*

C(36)-C(37)	*1.387(7)*
C(36)-H(36)	*0.9300*
C(37)-C(38)	*1.317(8)*
C(37)-H(37)	*0.9300*
C(38)-C(39)	*1.381(6)*
C(38)-H(38)	*0.9300*
C(39)-H(39)	*0.9300*
C(40)-C(41)	*1.476(6)*
C(40)-C(42)	*1.501(6)*
C(40)-C(43)	*1.513(6)*
C(41)-H(41A)	*0.9600*
C(41)-H(41B)	*0.9600*
C(41)-H(41C)	*0.9600*
C(42)-H(42A)	*0.9600*
C(42)-H(42B)	*0.9600*
C(42)-H(42C)	*0.9600*
C(43)-H(43A)	*0.9600*
C(43)-H(43B)	*0.9600*
C(43)-H(43C)	*0.9600*
C(7)-N(1)-C(2)	*121.6(4)*
C(7)-N(1)-H(1)	*119.2*
C(2)-N(1)-H(1)	*119.2*
C(30)-N(2)-S(1)	*116.6(2)*
C(30)-N(2)-H(2)	*107.5*
S(1)-N(2)-H(2)	*107.6*
C(1)-O(2)-C(6)	*110.6(9)*
O(4)-S(1)-N(2)	*112.98(17)*
O(4)-S(1)-C(40)	*105.37(19)*
N(2)-S(1)-C(40)	*98.09(17)*
O(2)-C(1)-O(3)	*135.5(14)*
O(2)-C(1)-C(2)	*111.2(8)*
O(3)-C(1)-C(2)	*112.4(10)*
N(1)-C(2)-C(3)	*113.4(5)*
N(1)-C(2)-C(1)	*105.4(5)*
C(3)-C(2)-C(1)	*116.7(5)*
N(1)-C(2)-H(2A)	*106.9*
C(3)-C(2)-H(2A)	*106.9*
C(1)-C(2)-H(2A)	*106.9*
C(5)-C(3)-C(2)	*109.3(4)*
C(5)-C(3)-C(4)	*112.5(5)*
C(2)-C(3)-C(4)	*111.6(4)*
C(5)-C(3)-H(3)	*107.7*
C(2)-C(3)-H(3)	*107.7*

C(4)-C(3)-H(3)	*107.7*
C(3)-C(4)-H(4A)	*109.5*
C(3)-C(4)-H(4B)	*109.5*
H(4A)-C(4)-H(4B)	*109.5*
C(3)-C(4)-H(4C)	*109.5*
H(4A)-C(4)-H(4C)	*109.5*
H(4B)-C(4)-H(4C)	*109.5*
C(3)-C(5)-H(5A)	*109.5*
C(3)-C(5)-H(5B)	*109.5*
H(5A)-C(5)-H(5B)	*109.5*
C(3)-C(5)-H(5C)	*109.5*
H(5A)-C(5)-H(5C)	*109.5*
H(5B)-C(5)-H(5C)	*109.5*
O(2)-C(6)-H(6A)	*109.5*
O(2)-C(6)-H(6B)	*109.5*
H(6A)-C(6)-H(6B)	*109.5*
O(2)-C(6)-H(6C)	*109.5*
H(6A)-C(6)-H(6C)	*109.5*
H(6B)-C(6)-H(6C)	*109.5*
O(1)-C(7)-N(1)	*122.5(4)*
O(1)-C(7)-C(8)	*121.4(4)*
N(1)-C(7)-C(8)	*116.1(4)*
C(13)-C(8)-C(9)	*119.0(4)*
C(13)-C(8)-C(7)	*118.1(4)*
C(9)-C(8)-C(7)	*122.8(4)*
C(10)-C(9)-C(8)	*120.3(4)*
C(10)-C(9)-H(9)	*119.8*
C(8)-C(9)-H(9)	*119.8*
C(9)-C(10)-C(11)	*120.5(4)*
C(9)-C(10)-H(10)	*119.7*
C(11)-C(10)-H(10)	*119.7*
C(12)-C(11)-C(10)	*118.8(4)*
C(12)-C(11)-C(14)	*119.8(4)*
C(10)-C(11)-C(14)	*121.0(4)*
C(11)-C(12)-C(13)	*120.8(4)*
C(11)-C(12)-H(12)	*119.6*
C(13)-C(12)-H(12)	*119.6*
C(8)-C(13)-C(12)	*120.5(4)*
C(8)-C(13)-H(13)	*119.7*
C(12)-C(13)-H(13)	*119.7*
C(15)-C(14)-C(19)	*119.3(4)*
C(15)-C(14)-C(11)	*115.2(4)*
C(19)-C(14)-C(11)	*125.3(4)*
C(14)-C(15)-C(16)	*121.6(5)*

C(14)-C(15)-H(15)	*119.2*
C(16)-C(15)-H(15)	*119.2*
C(17)-C(16)-C(15)	*119.9(6)*
C(17)-C(16)-H(16)	*120.0*
C(15)-C(16)-H(16)	*120.0*
C(16)-C(17)-C(18)	*121.0(5)*
C(16)-C(17)-H(17)	*119.5*
C(18)-C(17)-H(17)	*119.5*
C(23)-C(18)-C(17)	*119.3(5)*
C(23)-C(18)-C(19)	*121.2(5)*
C(17)-C(18)-C(19)	*119.5(5)*
C(14)-C(19)-C(18)	*117.8(5)*
C(14)-C(19)-C(20)	*125.7(4)*
C(18)-C(19)-C(20)	*116.4(5)*
C(21)-C(20)-C(19)	*119.2(4)*
C(21)-C(20)-C(24)	*116.5(4)*
C(19)-C(20)-C(24)	*124.0(4)*
C(20)-C(21)-C(22)	*122.2(5)*
C(20)-C(21)-H(21)	*118.9*
C(22)-C(21)-H(21)	*118.9*
C(23)-C(22)-C(21)	*120.0(5)*
C(23)-C(22)-H(22)	*120.0*
C(21)-C(22)-H(22)	*120.0*
C(22)-C(23)-C(18)	*120.5(5)*
C(22)-C(23)-H(23)	*119.7*
C(18)-C(23)-H(23)	*119.7*
C(25)-C(24)-C(29)	*117.4(4)*
C(25)-C(24)-C(20)	*121.8(4)*
C(29)-C(24)-C(20)	*120.5(4)*
C(24)-C(25)-C(26)	*122.2(4)*
C(24)-C(25)-H(25)	*118.9*
C(26)-C(25)-H(25)	*118.9*
C(27)-C(26)-C(25)	*119.9(4)*
C(27)-C(26)-H(26)	*120.1*
C(25)-C(26)-H(26)	*120.1*
C(26)-C(27)-C(28)	*117.7(4)*
C(26)-C(27)-C(30)	*124.3(4)*
C(28)-C(27)-C(30)	*118.0(4)*
C(29)-C(28)-C(27)	*121.8(4)*
C(29)-C(28)-H(28)	*119.1*
C(27)-C(28)-H(28)	*119.1*
C(28)-C(29)-C(24)	*121.0(4)*
C(28)-C(29)-H(29)	*119.5*
C(24)-C(29)-H(29)	*119.5*

N(2)-C(30)-C(34)	*108.9(3)*
N(2)-C(30)-C(31)	*108.6(3)*
C(34)-C(30)-C(31)	*107.6(3)*
N(2)-C(30)-C(27)	*110.4(3)*
C(34)-C(30)-C(27)	*108.1(3)*
C(31)-C(30)-C(27)	*113.1(3)*
C(30)-C(31)-C(32)	*113.9(3)*
C(30)-C(31)-H(31A)	*108.8*
C(32)-C(31)-H(31A)	*108.8*
C(30)-C(31)-H(31B)	*108.8*
C(32)-C(31)-H(31B)	*108.8*
H(31A)-C(31)-H(31B)	*107.7*
C(33)-C(32)-C(31)	*115.2(4)*
C(33)-C(32)-H(32A)	*108.5*
C(31)-C(32)-H(32A)	*108.5*
C(33)-C(32)-H(32B)	*108.5*
C(31)-C(32)-H(32B)	*108.5*
H(32A)-C(32)-H(32B)	*107.5*
C(32)-C(33)-H(33A)	*109.5*
C(32)-C(33)-H(33B)	*109.5*
H(33A)-C(33)-H(33B)	*109.5*
C(32)-C(33)-H(33C)	*109.5*
H(33A)-C(33)-H(33C)	*109.5*
H(33B)-C(33)-H(33C)	*109.5*
C(39)-C(34)-C(35)	*118.1(4)*
C(39)-C(34)-C(30)	*123.6(4)*
C(35)-C(34)-C(30)	*118.4(4)*
C(36)-C(35)-C(34)	*121.0(5)*
C(36)-C(35)-H(35)	*119.5*
C(34)-C(35)-H(35)	*119.5*
C(35)-C(36)-C(37)	*119.7(5)*
C(35)-C(36)-H(36)	*120.2*
C(37)-C(36)-H(36)	*120.2*
C(38)-C(37)-C(36)	*119.4(5)*
C(38)-C(37)-H(37)	*120.3*
C(36)-C(37)-H(37)	*120.3*
C(37)-C(38)-C(39)	*121.6(6)*
C(37)-C(38)-H(38)	*119.2*
C(39)-C(38)-H(38)	*119.2*
C(34)-C(39)-C(38)	*120.3(5)*
C(34)-C(39)-H(39)	*119.9*
C(38)-C(39)-H(39)	*119.9*
C(41)-C(40)-C(42)	*109.6(4)*
C(41)-C(40)-C(43)	*112.4(4)*

C(42)-C(40)-C(43)	*109.7(4)*
C(41)-C(40)-S(1)	*112.1(3)*
C(42)-C(40)-S(1)	*108.2(3)*
C(43)-C(40)-S(1)	*104.6(3)*
C(40)-C(41)-H(41A)	*109.5*
C(40)-C(41)-H(41B)	*109.5*
H(41A)-C(41)-H(41B)	*109.5*
C(40)-C(41)-H(41C)	*109.5*
H(41A)-C(41)-H(41C)	*109.5*
H(41B)-C(41)-H(41C)	*109.5*
C(40)-C(42)-H(42A)	*109.5*
C(40)-C(42)-H(42B)	*109.5*
H(42A)-C(42)-H(42B)	*109.5*
C(40)-C(42)-H(42C)	*109.5*
H(42A)-C(42)-H(42C)	*109.5*
H(42B)-C(42)-H(42C)	*109.5*
C(40)-C(43)-H(43A)	*109.5*
C(40)-C(43)-H(43B)	*109.5*
H(43A)-C(43)-H(43B)	*109.5*
C(40)-C(43)-H(43C)	*109.5*
H(43A)-C(43)-H(43C)	*109.5*
H(43B)-C(43)-H(43C)	*109.5*

Symmetry transformations used to generate equivalent atoms:

Table 4. Anisotropic displacement parameters (A^2 x 10^3) for 220723f. The anisotropic displacement factor exponent takes the form: -2 pi^2 [h^2 a*^2 U11 + ... + 2 h k a* b* U12]

	U11	U22	U33	U23	U13	U12
N(1)	118(4)	60(3)	66(2)	17(2)	37(2)	2(2)
N(2)	42(2)	44(2)	57(2)	-1(2)	2(2)	-8(2)
O(1)	97(3)	75(2)	68(2)	-9(2)	25(2)	-4(2)
O(2)	285(8)	164(5)	92(4)	29(3)	67(4)	128(5)
O(3)	166(5)	172(5)	163(4)	22(4)	21(4)	26(4)
O(4)	76(2)	101(2)	48(2)	-2(2)	13(2)	-15(2)
S(1)	53(1)	55(1)	59(1)	5(1)	-1(1)	-6(1)
C(1)	400(19)	109(6)	56(4)	-8(4)	104(8)	-108(9)
C(2)	104(5)	93(4)	56(3)	26(3)	22(3)	-14(4)
C(3)	126(5)	58(3)	79(4)	1(3)	29(4)	-20(4)
C(4)	136(6)	94(4)	95(4)	21(3)	33(4)	-37(4)
C(5)	119(5)	99(4)	94(4)	15(3)	-9(4)	-34(4)
C(6)	143(7)	109(5)	186(7)	53(5)	28(6)	32(5)
C(7)	62(3)	76(4)	52(3)	-3(3)	2(3)	-7(3)
C(8)	62(3)	47(3)	48(3)	-2(2)	3(2)	-5(2)
C(9)	62(3)	47(3)	55(3)	2(2)	2(2)	-1(2)
C(10)	63(3)	49(3)	56(3)	-1(2)	9(2)	-4(2)
C(11)	53(3)	48(3)	49(2)	-3(2)	-1(2)	0(2)
C(12)	71(3)	55(3)	55(3)	10(2)	2(3)	-7(3)
C(13)	56(3)	60(3)	66(3)	-1(2)	5(3)	2(3)
C(14)	78(4)	57(3)	36(2)	0(2)	-8(3)	-10(3)
C(15)	82(4)	92(4)	53(3)	2(3)	-9(3)	-10(3)
C(16)	128(6)	121(5)	56(4)	7(3)	-34(4)	-22(5)
C(17)	136(6)	103(4)	47(3)	5(3)	-6(4)	-33(5)
C(18)	90(4)	76(3)	46(3)	-3(2)	7(3)	-21(3)
C(19)	76(4)	54(3)	44(3)	-1(2)	5(3)	-7(3)
C(20)	72(4)	59(3)	40(2)	-7(2)	12(3)	-11(3)
C(21)	76(4)	82(4)	62(3)	-9(3)	15(3)	3(3)
C(22)	113(6)	102(4)	65(4)	-19(3)	38(4)	-11(4)

C(23)	*108(5)*	*105(4)*	*51(3)*	*-12(3)*	*18(4)*	*-21(4)*
C(24)	*53(3)*	*52(3)*	*46(2)*	*-6(2)*	*11(2)*	*-3(2)*
C(25)	*67(3)*	*55(3)*	*46(2)*	*5(2)*	*-10(2)*	*1(3)*
C(26)	*65(3)*	*49(3)*	*48(2)*	*-2(2)*	*-5(2)*	*4(2)*
C(27)	*43(3)*	*43(2)*	*46(2)*	*-4(2)*	*2(2)*	*-3(2)*
C(28)	*48(3)*	*54(3)*	*50(3)*	*-1(2)*	*-6(2)*	*3(2)*
C(29)	*63(3)*	*49(3)*	*55(3)*	*-5(2)*	*4(3)*	*11(3)*
C(30)	*46(3)*	*43(2)*	*47(2)*	*-6(2)*	*-3(2)*	*-4(2)*
C(31)	*54(3)*	*56(3)*	*58(3)*	*-13(2)*	*0(2)*	*-1(2)*
C(32)	*87(4)*	*93(4)*	*67(3)*	*-34(3)*	*-1(3)*	*19(3)*
C(33)	*130(6)*	*99(4)*	*85(4)*	*-32(3)*	*-2(4)*	*34(4)*
C(34)	*58(3)*	*53(3)*	*44(2)*	*-10(2)*	*9(2)*	*-12(2)*
C(35)	*76(4)*	*65(3)*	*72(3)*	*1(3)*	*18(3)*	*-22(3)*
C(36)	*90(5)*	*103(4)*	*82(4)*	*-8(3)*	*18(4)*	*-42(4)*
C(37)	*158(7)*	*72(4)*	*61(4)*	*3(3)*	*21(4)*	*-25(5)*
C(38)	*118(6)*	*73(4)*	*64(3)*	*0(3)*	*-9(4)*	*-17(4)*
C(39)	*92(4)*	*63(3)*	*47(2)*	*2(2)*	*-2(3)*	*-13(3)*
C(40)	*46(3)*	*62(3)*	*64(3)*	*1(2)*	*-10(2)*	*-9(2)*
C(41)	*53(4)*	*86(4)*	*127(4)*	*5(3)*	*-23(3)*	*1(3)*
C(42)	*89(4)*	*99(4)*	*80(3)*	*-16(3)*	*-11(3)*	*-6(4)*
C(43)	*82(4)*	*100(4)*	*109(4)*	*32(3)*	*-14(3)*	*-49(3)*

Table 5. Hydrogen coordinates (x 10^4) and isotropic displacement parameters (A^2 x 10^3) for 220723f.

	x	y	z	U(eq)
H(1)	4038	7293	6941	98
H(2)	-3451	4479	6673	57
H(2A)	5586	6760	6101	101
H(3)	6063	8266	6583	105
H(4A)	7404	7724	5656	162
H(4B)	7899	8511	5951	162
H(4C)	6303	8462	5700	162
H(5A)	7208	7232	7022	156
H(5B)	8454	7748	6750	156
H(5C)	7964	6912	6513	156
H(6A)	2045	8780	5615	219
H(6B)	920	8348	5991	219
H(6C)	1589	7873	5518	219
H(9)	2187	6844	7391	66
H(10)	1225	6514	8190	67
H(12)	4062	4648	8287	73
H(13)	5028	4978	7487	73
H(15)	4256	5358	9095	91
H(16)	3829	5280	9977	122
H(17)	1466	5399	10292	115
H(21)	-2882	5492	8757	88
H(22)	-3156	5648	9640	112
H(23)	-1135	5590	10166	105
H(25)	615	4134	8257	67
H(26)	335	3653	7424	65
H(28)	-2246	5566	7104	61
H(29)	-1989	6019	7930	67
H(31A)	-645	3083	6672	67
H(31B)	677	3666	6528	67
H(32A)	-1582	3219	5818	99
H(32B)	-207	3766	5679	99
H(33A)	1332	2665	5824	157
H(33B)	211	2529	5365	157

H(33C)	*-100*	*2130*	*5906*	*157*
H(35)	*1143*	*5125*	*6536*	*85*
H(36)	*1875*	*6112*	*5968*	*110*
H(37)	*211*	*6576*	*5342*	*116*
H(38)	*-2110*	*6062*	*5310*	*102*
H(39)	*-2855*	*5053*	*5868*	*81*
H(41A)	*-5577*	*4200*	*6251*	*133*
H(41B)	*-6381*	*3964*	*6770*	*133*
H(41C)	*-7013*	*3653*	*6239*	*133*
H(42A)	*-5570*	*2634*	*5739*	*134*
H(42B)	*-4048*	*2320*	*5965*	*134*
H(42C)	*-4187*	*3218*	*5768*	*134*
H(43A)	*-6789*	*2173*	*6535*	*145*
H(43B)	*-6325*	*2540*	*7072*	*145*
H(43C)	*-5273*	*1916*	*6793*	*145*

Table 6. Torsion angles [deg] for 220723f.

C(30)-N(2)-S(1)-O(4)	*89.5(3)*
C(30)-N(2)-S(1)-C(40)	*-160.0(3)*
C(6)-O(2)-C(1)-O(3)	*14.9(15)*
C(6)-O(2)-C(1)-C(2)	*-177.5(4)*
C(7)-N(1)-C(2)-C(3)	*-117.8(5)*
C(7)-N(1)-C(2)-C(1)	*113.4(5)*
O(2)-C(1)-C(2)-N(1)	*51.6(8)*
O(3)-C(1)-C(2)-N(1)	*-137.8(6)*
O(2)-C(1)-C(2)-C(3)	*-75.3(9)*
O(3)-C(1)-C(2)-C(3)	*95.3(7)*
N(1)-C(2)-C(3)-C(5)	*60.0(6)*
C(1)-C(2)-C(3)-C(5)	*-177.2(5)*
N(1)-C(2)-C(3)-C(4)	*-174.9(5)*
C(1)-C(2)-C(3)-C(4)	*-52.1(7)*
C(2)-N(1)-C(7)-O(1)	*-0.5(7)*
C(2)-N(1)-C(7)-C(8)	*178.3(4)*
O(1)-C(7)-C(8)-C(13)	*30.1(6)*
N(1)-C(7)-C(8)-C(13)	*-148.7(4)*
O(1)-C(7)-C(8)-C(9)	*-152.7(4)*
N(1)-C(7)-C(8)-C(9)	*28.5(6)*
C(13)-C(8)-C(9)-C(10)	*1.3(6)*
C(7)-C(8)-C(9)-C(10)	*-175.9(4)*
C(8)-C(9)-C(10)-C(11)	*0.4(6)*
C(9)-C(10)-C(11)-C(12)	*-2.1(6)*
C(9)-C(10)-C(11)-C(14)	*170.8(4)*
C(10)-C(11)-C(12)-C(13)	*2.1(6)*
C(14)-C(11)-C(12)-C(13)	*-170.9(4)*
C(9)-C(8)-C(13)-C(12)	*-1.2(6)*
C(7)-C(8)-C(13)-C(12)	*176.1(4)*
C(11)-C(12)-C(13)-C(8)	*-0.4(6)*
C(12)-C(11)-C(14)-C(15)	*48.4(5)*
C(10)-C(11)-C(14)-C(15)	*-124.4(5)*
C(12)-C(11)-C(14)-C(19)	*-136.8(5)*
C(10)-C(11)-C(14)-C(19)	*50.3(6)*
C(19)-C(14)-C(15)-C(16)	*-3.3(7)*
C(11)-C(14)-C(15)-C(16)	*171.8(4)*
C(14)-C(15)-C(16)-C(17)	*-3.2(8)*
C(15)-C(16)-C(17)-C(18)	*3.2(9)*
C(16)-C(17)-C(18)-C(23)	*-176.3(6)*

C(16)-C(17)-C(18)-C(19)	*3.2(8)*
C(15)-C(14)-C(19)-C(18)	*9.5(6)*
C(11)-C(14)-C(19)-C(18)	*-165.1(4)*
C(15)-C(14)-C(19)-C(20)	*-173.9(4)*
C(11)-C(14)-C(19)-C(20)	*11.5(7)*
C(23)-C(18)-C(19)-C(14)	*170.1(5)*
C(17)-C(18)-C(19)-C(14)	*-9.5(7)*
C(23)-C(18)-C(19)-C(20)	*-6.9(7)*
C(17)-C(18)-C(19)-C(20)	*173.6(4)*
C(14)-C(19)-C(20)-C(21)	*-168.5(4)*
C(18)-C(19)-C(20)-C(21)	*8.2(6)*
C(14)-C(19)-C(20)-C(24)	*18.0(7)*
C(18)-C(19)-C(20)-C(24)	*-165.4(4)*
C(19)-C(20)-C(21)-C(22)	*-5.1(7)*
C(24)-C(20)-C(21)-C(22)	*169.0(4)*
C(20)-C(21)-C(22)-C(23)	*0.0(8)*
C(21)-C(22)-C(23)-C(18)	*1.5(9)*
C(17)-C(18)-C(23)-C(22)	*-178.4(5)*
C(19)-C(18)-C(23)-C(22)	*2.1(8)*
C(21)-C(20)-C(24)-C(25)	*-124.1(5)*
C(19)-C(20)-C(24)-C(25)	*49.6(6)*
C(21)-C(20)-C(24)-C(29)	*49.3(6)*
C(19)-C(20)-C(24)-C(29)	*-136.9(4)*
C(29)-C(24)-C(25)-C(26)	*-2.3(6)*
C(20)-C(24)-C(25)-C(26)	*171.4(4)*
C(24)-C(25)-C(26)-C(27)	*1.0(7)*
C(25)-C(26)-C(27)-C(28)	*1.0(6)*
C(25)-C(26)-C(27)-C(30)	*-177.3(4)*
C(26)-C(27)-C(28)-C(29)	*-1.5(6)*
C(30)-C(27)-C(28)-C(29)	*176.9(4)*
C(27)-C(28)-C(29)-C(24)	*0.2(6)*
C(25)-C(24)-C(29)-C(28)	*1.7(6)*
C(20)-C(24)-C(29)-C(28)	*-172.0(4)*
S(1)-N(2)-C(30)-C(34)	*171.5(2)*
S(1)-N(2)-C(30)-C(31)	*54.6(3)*
S(1)-N(2)-C(30)-C(27)	*-69.9(3)*
C(26)-C(27)-C(30)-N(2)	*112.8(4)*
C(28)-C(27)-C(30)-N(2)	*-65.5(4)*
C(26)-C(27)-C(30)-C(34)	*-128.1(4)*
C(28)-C(27)-C(30)-C(34)	*53.6(5)*
C(26)-C(27)-C(30)-C(31)	*-9.0(5)*
C(28)-C(27)-C(30)-C(31)	*172.7(4)*
N(2)-C(30)-C(31)-C(32)	*63.2(4)*
C(34)-C(30)-C(31)-C(32)	*-54.6(5)*

C(27)-C(30)-C(31)-C(32)	*-173.9(3)*
C(30)-C(31)-C(32)-C(33)	*-177.2(5)*
N(2)-C(30)-C(34)-C(39)	*-7.3(5)*
C(31)-C(30)-C(34)-C(39)	*110.3(4)*
C(27)-C(30)-C(34)-C(39)	*-127.2(4)*
N(2)-C(30)-C(34)-C(35)	*174.9(3)*
C(31)-C(30)-C(34)-C(35)	*-67.6(4)*
C(27)-C(30)-C(34)-C(35)	*54.9(5)*
C(39)-C(34)-C(35)-C(36)	*-1.9(7)*
C(30)-C(34)-C(35)-C(36)	*176.0(4)*
C(34)-C(35)-C(36)-C(37)	*1.0(8)*
C(35)-C(36)-C(37)-C(38)	*0.6(8)*
C(36)-C(37)-C(38)-C(39)	*-1.2(8)*
C(35)-C(34)-C(39)-C(38)	*1.3(6)*
C(30)-C(34)-C(39)-C(38)	*-176.6(4)*
C(37)-C(38)-C(39)-C(34)	*0.3(8)*
O(4)-S(1)-C(40)-C(41)	*65.9(4)*
N(2)-S(1)-C(40)-C(41)	*-50.7(4)*
O(4)-S(1)-C(40)-C(42)	*-173.2(3)*
N(2)-S(1)-C(40)-C(42)	*70.2(3)*
O(4)-S(1)-C(40)-C(43)	*-56.2(4)*
N(2)-S(1)-C(40)-C(43)	*-172.8(3)*

Symmetry transformations used to generate equivalent atoms:

Table 7. Hydrogen bonds for 220723f [A and deg.].

D-H	*d(D-H)*	*d(H..A)*	*<DHA*	*d(D..A)*	*A*
N1-H1	*0.860*	*2.502*	*153.68*	*3.295*	*O4 [-x, y+1/2, -z+3/2]*
N2-H2	*0.900*	*2.352*	*154.47*	*3.187*	*O1 [x-1, y, z]*

----- Write up for **19**

General Data Collection

Data were collected on a Rigaku XtaLAB Synergy-*i* Kappa diffractometer equipped with a PhotonJet-*i* X-ray source operated at 50 W (50kV, 1 mA) to generate Cu Kα radiation (λ = 1.54178 Å) and a HyPix-6000HE HPC detector. Crystals were transferred from the vial and placed on a glass slide in type NVH immersion oil by Cargille. A Zeiss Stemi 305 microscope was used to identify a suitable specimen for X-ray diffraction from a representative sample of the material. The crystal and a small amount of the oil were collected on a 100 micron MiTeGen cryoloop and transferred to the instrument where it was placed under a cold nitrogen stream (Oxford 700 series) maintained at 100K throughout the duration of the experiment. The sample was optically centered with the aid of a video camera to insure that no translations were observed as the crystal was rotated through all positions.

A unit cell collection was then carried out. After it was determined that the unit cell was not present in the CCDC database a data collection strategy was calculated by *CrysAlis*^{Pro}[1]. The crystal was measured for size, morphology, and color. These values are reported in the accompanying Li23_01_tables file.

Refinement Details

After data collection, the unit cell was re-determined using a subset of the full data collection. Intensity data were corrected for Lorentz, polarization, and background effects using the *CrysAlis*^{Pro}[1]. A numerical absorption correction was applied based on a Gaussian integration over a multifaceted crystal and followed by a semi-empirical correction for adsorption applied using the program *SCALE3 ABSPACK*[2]. The programs *SHELXT*[3] was used for the initial structure solution and *SHELXL*[4] was used for refinement of the structure. Both of these programs were utilized within the OLEX2 software[5]. Hydrogen atoms bound to the carbon and nitrogen atoms were located in the difference Fourier map where possible and were geometrically constrained using the appropriate AFIX commands.

References:

1. CrysAlis^{Pro} (2018) Oxford Diffraction Ltd.
2. SCALE3 ABSPACK (2005) Oxford Diffraction Ltd.
3. Sheldrick, G. M. (2015) *Acta Crystallogr.*, C**71**, 3-8.
4. Sheldrick, G. M. (2015) *Acta Crystallogr.*, A**71**, 3-8.
5. Dolomanov, O. V.; Bourhis, . L. J.; Gildea, R. J.; Howard, J. A. K.; Puschmann. H. (2009) *J. Appl. Cryst.* **42**, 339-341.

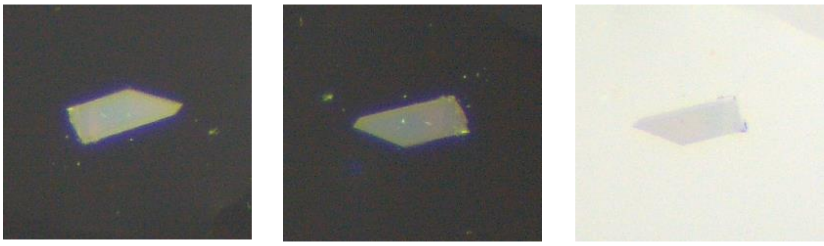

Crystal used for single crystal X-ray diffraction experiment.

Li23_01

Table 1 Crystal data and structure refinement for Li23_01.

Identification code	Li23_01
Empirical formula	$C_{46}H_{46}N_2O_4S$

Formula weight	722.91
Temperature/K	100.3(6)
Crystal system	orthorhombic
Space group	$P2_12_12_1$
a/Å	15.35810(10)
b/Å	15.62710(10)
c/Å	16.10440(10)
α/°	90
β/°	90
γ/°	90
Volume/Å^3	3865.10(4)
Z	4
ρ_{calc}g/cm^3	1.242
μ/mm^{-1}	1.107
F(000)	1536.0
Crystal size/mm^3	0.257 × 0.116 × 0.115
Radiation	Cu Kα (λ = 1.54184)
2Θ range for data collection/°	7.884 to 154.96
Index ranges	-14 ≤ h ≤ 18, -19 ≤ k ≤ 17, -19 ≤ l ≤ 20
Reflections collected	56496
Independent reflections	8103 [R_{int} = 0.0370, R_{sigma} = 0.0205]
Data/restraints/parameters	8103/0/488
Goodness-of-fit on F^2	1.061
Final R indexes [I>=2σ (I)]	R_1 = 0.0282, wR_2 = 0.0709
Final R indexes [all data]	R_1 = 0.0295, wR_2 = 0.0715
Largest diff. peak/hole / e Å^{-3}	0.15/-0.36
Flack parameter	0.005(4)

Table 2 Fractional Atomic Coordinates (×10^4) and Equivalent Isotropic Displacement Parameters (Å^2×10^3) for Li23_01. U_{eq} is defined as 1/3 of the trace of the orthogonalised U_{IJ} tensor.

Atom	*x*	*y*	*z*	U(eq)
S1	809.2(3)	6515.3(3)	5323.7(3)	18.96(10)
O2	1741.8(8)	4013.8(8)	4852.8(8)	23.7(3)
O1	5230.1(9)	4995.3(9)	3041.6(9)	26.7(3)
O3	2964.3(9)	4395.2(9)	4178.0(9)	27.5(3)
O4	962.8(8)	6907.6(9)	6158.9(9)	26.8(3)
N2	1324.8(9)	5595.1(10)	5239.0(10)	18.3(3)
N1	5816.9(10)	6207.6(11)	3561.1(10)	24.9(3)

Table 2 Fractional Atomic Coordinates (×10⁴) and Equivalent Isotropic Displacement Parameters (Å²×10³) for Li23_01. U_{eq} is defined as 1/3 of the trace of the orthogonalised U_{IJ} tensor.

Atom	*x*	*y*	*z*	U(eq)
C27	1342.3(11)	6087.9(12)	2632.5(12)	19.6(4)
C32	2154.4(11)	5516.2(11)	4790.4(11)	17.3(3)
C4	469.0(13)	8334.1(12)	522.9(13)	25.1(4)
C11	3347.4(11)	7197.7(12)	1646.7(11)	18.1(4)
C29	2052.8(11)	5995.3(11)	3967.6(11)	16.9(3)
C9	2766.1(12)	7692.7(11)	1076.9(11)	18.9(3)
C20	7302.3(12)	5672.4(12)	3319.9(12)	21.1(4)
C14	4574.4(11)	6304.8(12)	2616.1(11)	19.5(4)
C37	2950.8(11)	5783.8(11)	5310.5(12)	19.9(4)
C1	1300.7(12)	7420.5(11)	1798.3(12)	19.4(4)
C12	3272.3(11)	6315.0(11)	1769.2(11)	18.4(4)
C31	2130.8(11)	7296.9(12)	3157.1(11)	18.9(4)
C16	4047.4(12)	7622.9(12)	2016.6(12)	20.5(4)
C28	1552.1(11)	5625.8(12)	3338.1(11)	19.5(4)
C26	1623.5(11)	6929.6(12)	2533.4(11)	18.4(4)
C33	2248.7(11)	4546.9(11)	4612.4(11)	18.1(3)
C10	1834.2(12)	7773.3(11)	1148.2(11)	18.4(4)
C15	4655.2(12)	7181.5(12)	2491.7(11)	21.2(4)
C17	5230.4(12)	5782.0(13)	3091.9(11)	21.2(4)
C6	1863.8(14)	8649.1(12)	-132.7(12)	25.9(4)
C13	3869.5(11)	5877.5(12)	2248.4(11)	19.5(4)
C30	2343.9(11)	6831.7(12)	3861.8(11)	18.5(3)
C3	-16.7(13)	7976.5(13)	1137.0(14)	27.7(4)
C5	1390.0(13)	8254.2(11)	513.6(12)	21.8(4)
C42	3772.1(13)	5814.9(14)	4944.2(13)	28.6(4)
C43	-311.7(11)	6090.3(12)	5353.3(12)	21.3(4)
C2	407.5(12)	7526.5(13)	1775.2(13)	24.8(4)
C18	6554.7(12)	5756.9(14)	3931.7(12)	25.2(4)
C38	2881.4(13)	5945.7(13)	6156.8(12)	24.3(4)
C7	2748.7(14)	8587.8(13)	-168.4(12)	27.1(4)
C8	3191.5(13)	8105.4(12)	436.9(12)	23.8(4)
C34	3288.6(13)	3511.9(12)	4113.7(13)	25.3(4)
C45	-487.5(13)	5669.6(14)	4515.5(13)	27.6(4)
C25	7656.4(13)	4886.2(13)	3129.2(13)	26.7(4)
C21	7664.8(13)	6402.8(13)	2960.4(14)	27.7(4)
C39	3618.4(14)	6126.5(14)	6627.4(14)	32.2(5)

Table 2 Fractional Atomic Coordinates (×10^4) and Equivalent Isotropic Displacement Parameters (Å^2×10^3) for Li23_01. U_{eq} is defined as 1/3 of the trace of the orthogonalised U_{IJ} tensor.

Atom	*x*	*y*	*z*	U(eq)
C22	8363.7(14)	6332.7(15)	2422.8(15)	34.4(5)
C44	-425.9(13)	5490.9(14)	6087.6(13)	26.9(4)
C19	6859.9(13)	6220.3(18)	4716.4(15)	39.8(6)
C35	4229.8(14)	3546.9(15)	4364.0(16)	36.4(5)
C46	-879.2(14)	6886.1(14)	5457.4(15)	33.1(5)
C23	8718.7(13)	5541.9(17)	2243.7(13)	34.2(5)
C24	8366.9(14)	4818.0(15)	2596.9(15)	33.3(5)
C40	4430.7(14)	6146.6(16)	6262.7(15)	37.3(5)
C41	4501.9(14)	5988.4(17)	5417.8(15)	39.1(5)
C36	3155(2)	3217.6(18)	3241.5(17)	50.2(7)

Table 3 Anisotropic Displacement Parameters (Å^2×10^3) for Li23_01. The Anisotropic displacement factor exponent takes the form: -2π^2[h^{2}a*^{2}U_{11}+2hka*b*U_{12}+…].

Atom	U_{11}	U_{22}	U_{33}	U_{23}	U_{13}	U_{12}
S1	19.70(19)	14.97(19)	22.2(2)	-1.42(16)	2.26(17)	1.23(16)
O2	23.9(7)	19.4(6)	27.9(7)	1.1(5)	3.8(5)	-0.3(5)
O1	27.4(7)	22.5(7)	30.2(7)	1.3(6)	-6.9(6)	0.9(5)
O3	28.1(7)	16.9(6)	37.6(8)	1.6(6)	15.2(6)	4.8(6)
O4	21.8(7)	26.6(7)	32.0(8)	-12.7(6)	0.0(5)	0.7(6)
N2	18.1(7)	18.0(7)	18.6(7)	2.0(6)	4.4(6)	1.3(6)
N1	18.3(7)	27.2(8)	29.2(9)	-7.2(7)	-5.3(7)	3.6(7)
C27	19.6(9)	18.9(9)	20.3(9)	-1.6(7)	-0.1(7)	-2.2(7)
C32	17.0(8)	16.4(8)	18.5(8)	-0.1(7)	2.9(7)	1.7(6)
C4	29.9(10)	17.5(9)	28.0(10)	1.2(7)	-8.2(8)	5.0(7)
C11	17.5(8)	20.0(9)	16.7(8)	-1.7(7)	4.3(6)	1.1(7)
C29	16.3(8)	16.9(8)	17.6(8)	-0.5(7)	3.0(6)	1.3(6)
C9	23.3(9)	14.4(8)	19.0(9)	-1.9(7)	1.0(7)	-0.2(7)
C20	16.9(8)	23.3(9)	23.1(9)	2.7(7)	-4.6(7)	1.0(7)
C14	17.9(8)	23.4(9)	17.1(9)	-3.8(7)	2.5(7)	1.7(7)
C37	21.0(8)	17.4(8)	21.3(9)	3.5(7)	-2.0(7)	1.5(6)
C1	21.3(9)	15.8(9)	21.1(9)	-1.3(7)	-1.0(7)	-0.3(7)
C12	17.6(8)	19.9(9)	17.6(9)	-3.9(7)	1.2(7)	-1.8(7)
C31	18.3(8)	15.6(8)	22.8(9)	-0.4(7)	2.8(7)	-2.0(6)
C16	21.7(9)	18.4(8)	21.3(9)	-0.9(7)	3.0(7)	-2.0(7)
C28	20.7(9)	16.2(8)	21.6(9)	-0.8(7)	2.1(7)	-2.7(7)
C26	16.4(8)	20.1(9)	18.5(9)	1.0(7)	2.7(6)	0.8(7)

Table 3 Anisotropic Displacement Parameters (Å²×10³) for Li23_01. The Anisotropic displacement factor exponent takes the form: -2π²[h²a*²U11+2hka*b*U12+…].

Atom	U_{11}	U_{22}	U_{33}	U_{23}	U_{13}	U_{12}
C33	18.4(8)	20.6(9)	15.4(8)	1.7(7)	0.3(7)	2.8(7)
C10	23.0(9)	13.9(8)	18.2(8)	-2.5(7)	-1.3(7)	0.3(7)
C15	17.6(8)	24.2(10)	21.7(9)	-4.2(7)	1.4(7)	-2.8(7)
C17	18.0(8)	25.9(10)	19.6(9)	-2.4(7)	0.9(7)	1.1(7)
C6	37.6(11)	18.2(9)	21.8(10)	2.9(7)	-3.4(8)	3.5(8)
C13	19.6(8)	19.0(9)	19.9(9)	-1.9(7)	1.9(7)	1.1(7)
C30	18.0(8)	19.4(9)	18.1(8)	-2.5(7)	0.1(7)	-2.2(7)
C3	22.0(9)	25.6(10)	35.5(12)	2.8(9)	-3.2(8)	2.5(8)
C5	27.7(9)	14.4(8)	23.4(9)	-2.1(7)	-2.3(7)	1.0(7)
C42	22.0(10)	38.2(12)	25.6(10)	3.4(9)	-0.3(8)	-2.9(8)
C43	18.1(8)	20.8(9)	24.8(9)	-4.2(8)	-2.4(7)	3.5(7)
C2	22.2(9)	24.9(10)	27.4(10)	3.8(8)	0.6(8)	0.8(7)
C18	18.2(9)	33.0(11)	24.4(10)	-0.5(8)	-3.7(7)	1.6(8)
C38	23.7(9)	25.4(10)	23.7(9)	-2.0(8)	-0.9(7)	3.5(8)
C7	36.5(11)	23.1(10)	21.5(10)	3.0(8)	5.4(8)	-2.1(8)
C8	25.9(9)	22.4(9)	22.9(10)	-0.9(8)	4.5(8)	-0.4(7)
C34	28.4(10)	16.2(9)	31.4(10)	1.3(8)	8.0(8)	6.6(8)
C45	26.6(10)	28.8(10)	27.3(10)	-6.7(8)	-8.2(8)	5.2(8)
C25	25.4(9)	22.3(10)	32.4(11)	2.9(8)	-1.2(8)	-0.4(8)
C21	23.9(9)	23.0(10)	36.3(11)	4.1(9)	-3.5(8)	0.8(8)
C39	34.2(11)	34.9(12)	27.4(11)	-3.0(9)	-8.4(9)	3.6(9)
C22	26.9(10)	39.7(13)	36.5(12)	16.3(10)	-1.1(9)	-5.4(9)
C44	22.4(9)	29.0(11)	29.3(10)	-1.4(8)	5.7(8)	-5.2(8)
C19	20.6(10)	69.4(17)	29.3(11)	-12.1(11)	-5.5(9)	3.7(10)
C35	30.3(11)	29.8(11)	49.1(13)	7.9(10)	3.0(10)	4.2(10)
C46	25.2(10)	30.1(11)	44.1(13)	-14.8(9)	-9.3(9)	8.7(9)
C23	23.0(10)	54.2(15)	25.4(11)	2.3(10)	2.5(8)	2.6(9)
C24	29.5(11)	32.9(12)	37.5(12)	-5.9(9)	1.0(9)	6.6(9)
C40	26.8(11)	47.5(14)	37.6(13)	3.3(10)	-13.8(9)	-2.5(10)
C41	21.7(10)	57.5(15)	38.1(13)	6.0(12)	-2.7(9)	-5.8(10)
C36	70.2(18)	39.3(14)	41.1(14)	-10.5(11)	-9.8(13)	22.8(13)

Table 4 Bond Lengths for Li23_01.

Atom	Atom	Length/Å	Atom	Atom	Length/Å
S1	O4	1.4968(14)	C14	C13	1.403(3)
S1	N2	1.6473(15)	C37	C42	1.393(3)
S1	C43	1.8458(19)	C37	C38	1.390(3)

Table 4 Bond Lengths for Li23_01.

Atom	Atom	Length/Å	Atom	Atom	Length/Å
O2	C33	1.204(2)	C1	C26	1.495(3)
O1	C17	1.232(2)	C1	C10	1.439(3)
O3	C33	1.324(2)	C1	C2	1.382(3)
O3	C34	1.471(2)	C12	C13	1.380(3)
N2	C32	1.470(2)	C31	C26	1.395(3)
N1	C17	1.351(2)	C31	C30	1.387(3)
N1	C18	1.461(2)	C16	C15	1.390(3)
C27	C28	1.384(3)	C10	C5	1.440(3)
C27	C26	1.394(3)	C6	C5	1.412(3)
C32	C29	1.530(2)	C6	C7	1.364(3)
C32	C37	1.540(2)	C3	C2	1.406(3)
C32	C33	1.548(2)	C42	C41	1.383(3)
C4	C3	1.359(3)	C43	C45	1.525(3)
C4	C5	1.420(3)	C43	C44	1.519(3)
C11	C9	1.496(2)	C43	C46	1.528(3)
C11	C12	1.398(3)	C18	C19	1.530(3)
C11	C16	1.397(3)	C38	C39	1.391(3)
C29	C28	1.397(2)	C7	C8	1.407(3)
C29	C30	1.392(2)	C34	C35	1.502(3)
C9	C10	1.441(3)	C34	C36	1.492(3)
C9	C8	1.380(3)	C25	C24	1.392(3)
C20	C18	1.519(3)	C21	C22	1.383(3)
C20	C25	1.378(3)	C39	C40	1.379(3)
C20	C21	1.396(3)	C22	C23	1.381(3)
C14	C15	1.390(3)	C23	C24	1.377(3)
C14	C17	1.507(3)	C40	C41	1.387(4)

Table 5 Bond Angles for Li23_01.

Atom	Atom	Atom	Angle/°	Atom	Atom	Atom	Angle/°
O4	S1	N2	110.87(8)	O2	C33	O3	125.63(17)
O4	S1	C43	105.74(8)	O2	C33	C32	123.85(16)
N2	S1	C43	97.84(8)	O3	C33	C32	110.51(15)
C33	O3	C34	119.08(14)	C1	C10	C9	126.15(17)
C32	N2	S1	122.05(12)	C1	C10	C5	116.50(16)
C17	N1	C18	120.54(17)	C5	C10	C9	117.35(17)
C28	C27	C26	120.94(17)	C16	C15	C14	120.55(17)
N2	C32	C29	107.23(14)	O1	C17	N1	121.89(18)
N2	C32	C37	113.46(14)	O1	C17	C14	120.49(17)

Table 5 Bond Angles for Li23_01.

Atom	Atom	Atom	Angle/°	Atom	Atom	Atom	Angle/°
N2	C32	C33	104.72(14)	N1	C17	C14	117.62(17)
C29	C32	C37	114.78(15)	C7	C6	C5	120.93(18)
C29	C32	C33	109.15(14)	C12	C13	C14	120.87(17)
C37	C32	C33	106.98(14)	C31	C30	C29	121.10(16)
C3	C4	C5	121.22(18)	C4	C3	C2	118.92(18)
C12	C11	C9	123.20(16)	C4	C5	C10	120.68(18)
C16	C11	C9	118.35(16)	C6	C5	C4	118.86(18)
C16	C11	C12	118.19(17)	C6	C5	C10	120.46(18)
C28	C29	C32	118.84(16)	C41	C42	C37	120.48(19)
C30	C29	C32	122.19(16)	C45	C43	S1	107.30(13)
C30	C29	C28	118.43(16)	C45	C43	C46	110.31(16)
C10	C9	C11	126.08(16)	C44	C43	S1	110.47(13)
C8	C9	C11	114.66(16)	C44	C43	C45	113.74(16)
C8	C9	C10	119.25(17)	C44	C43	C46	110.53(17)
C25	C20	C18	121.35(18)	C46	C43	S1	104.00(13)
C25	C20	C21	118.63(18)	C1	C2	C3	122.65(19)
C21	C20	C18	119.98(18)	N1	C18	C20	111.29(16)
C15	C14	C17	123.23(17)	N1	C18	C19	110.28(17)
C15	C14	C13	118.50(17)	C20	C18	C19	110.20(16)
C13	C14	C17	118.20(17)	C37	C38	C39	120.57(19)
C42	C37	C32	119.87(17)	C6	C7	C8	119.34(18)
C38	C37	C32	121.45(16)	C9	C8	C7	122.60(18)
C38	C37	C42	118.55(18)	O3	C34	C35	105.83(16)
C10	C1	C26	125.70(16)	O3	C34	C36	108.00(17)
C2	C1	C26	114.32(17)	C36	C34	C35	113.3(2)
C2	C1	C10	119.98(17)	C20	C25	C24	120.99(19)
C13	C12	C11	120.86(17)	C22	C21	C20	120.29(19)
C30	C31	C26	120.35(16)	C40	C39	C38	120.6(2)
C15	C16	C11	121.03(17)	C23	C22	C21	120.5(2)
C27	C28	C29	120.53(17)	C24	C23	C22	119.61(19)
C27	C26	C1	118.17(17)	C23	C24	C25	120.0(2)
C27	C26	C31	118.63(17)	C39	C40	C41	119.0(2)
C31	C26	C1	122.97(16)	C42	C41	C40	120.8(2)

Table 6 Torsion Angles for Li23_01.

A	B	C	D	Angle/°	A	B	C	D	Angle/°
S1	N2	C32	C29	-46.07(18)	C26	C31	C30	C29	-0.6(3)
S1	N2	C32	C37	81.75(17)	C33	O3	C34	C35	-128.04(18)

Table 6 Torsion Angles for Li23_01.

A	B	C	D	Angle/°	A	B	C	D	Angle/°
S1	N2	C32	C33	-161.95(12)	C33	O3	C34	C36	110.3(2)
O4	S1	N2	C32	-100.95(14)	C33	C32	C29	C28	38.8(2)
O4	S1	C43	C45	-179.00(13)	C33	C32	C29	C30	-149.84(16)
O4	S1	C43	C44	-54.50(15)	C33	C32	C37	C42	70.7(2)
O4	S1	C43	C46	64.12(15)	C33	C32	C37	C38	-105.22(19)
N2	S1	C43	C45	-64.64(14)	C10	C9	C8	C7	-1.8(3)
N2	S1	C43	C44	59.85(14)	C10	C1	C26	C27	-118.9(2)
N2	S1	C43	C46	178.47(14)	C10	C1	C26	C31	66.7(3)
N2	C32	C29	C28	-74.14(19)	C10	C1	C2	C3	-0.4(3)
N2	C32	C29	C30	97.25(19)	C15	C14	C17	O1	-164.89(18)
N2	C32	C37	C42	-174.35(17)	C15	C14	C17	N1	14.3(3)
N2	C32	C37	C38	9.7(2)	C15	C14	C13	C12	0.4(3)
N2	C32	C33	O2	-3.5(2)	C17	N1	C18	C20	83.8(2)
N2	C32	C33	O3	177.65(14)	C17	N1	C18	C19	-153.53(19)
C32	C29	C28	C27	170.83(16)	C17	C14	C15	C16	177.22(17)
C32	C29	C30	C31	-170.22(16)	C17	C14	C13	C12	-176.63(16)
C32	C37	C42	C41	-174.9(2)	C6	C7	C8	C9	-0.6(3)
C32	C37	C38	C39	175.35(18)	C13	C14	C15	C16	0.3(3)
C4	C3	C2	C1	-1.0(3)	C13	C14	C17	O1	12.0(3)
C11	C9	C10	C1	2.6(3)	C13	C14	C17	N1	-168.75(16)
C11	C9	C10	C5	-177.64(16)	C30	C29	C28	C27	-0.9(3)
C11	C9	C8	C7	178.77(17)	C30	C31	C26	C27	-0.4(3)
C11	C12	C13	C14	-0.9(3)	C30	C31	C26	C1	174.02(17)
C11	C16	C15	C14	-0.6(3)	C3	C4	C5	C10	1.4(3)
C29	C32	C37	C42	-50.6(2)	C3	C4	C5	C6	-179.09(19)
C29	C32	C37	C38	133.53(18)	C5	C4	C3	C2	0.5(3)
C29	C32	C33	O2	-118.00(19)	C5	C6	C7	C8	1.6(3)
C29	C32	C33	O3	63.11(19)	C42	C37	C38	C39	-0.6(3)
C9	C11	C12	C13	174.61(16)	C43	S1	N2	C32	148.84(14)
C9	C11	C16	C15	-174.16(17)	C2	C1	C26	C27	61.3(2)
C9	C10	C5	C4	177.53(17)	C2	C1	C26	C31	-113.1(2)
C9	C10	C5	C6	-2.0(3)	C2	C1	C10	C9	-178.05(18)
C20	C25	C24	C23	-0.9(3)	C2	C1	C10	C5	2.2(3)
C20	C21	C22	C23	-0.9(3)	C18	N1	C17	O1	9.0(3)
C37	C32	C29	C28	158.82(16)	C18	N1	C17	C14	-170.17(16)
C37	C32	C29	C30	-29.8(2)	C18	C20	C25	C24	-177.06(19)
C37	C32	C33	O2	117.25(19)	C18	C20	C21	C22	177.96(18)
C37	C32	C33	O3	-61.64(18)	C38	C37	C42	C41	1.1(3)

Table 6 Torsion Angles for Li23_01.

A	B	C	D	Angle/°	A	B	C	D	Angle/°
C37	C42	C41	C40	-0.9(4)	C38	C39	C40	C41	0.4(4)
C37	C38	C39	C40	-0.1(3)	C7	C6	C5	C4	-179.83(19)
C1	C10	C5	C4	-2.7(3)	C7	C6	C5	C10	-0.3(3)
C1	C10	C5	C6	177.78(17)	C8	C9	C10	C1	-176.77(17)
C12	C11	C9	C10	60.9(3)	C8	C9	C10	C5	2.9(3)
C12	C11	C9	C8	-119.71(19)	C34	O3	C33	O2	-13.4(3)
C12	C11	C16	C15	0.2(3)	C34	O3	C33	C32	165.45(16)
C16	C11	C9	C10	-125.09(19)	C25	C20	C18	N1	-124.9(2)
C16	C11	C9	C8	54.3(2)	C25	C20	C18	C19	112.4(2)
C16	C11	C12	C13	0.5(3)	C25	C20	C21	C22	0.2(3)
C28	C27	C26	C1	-173.97(17)	C21	C20	C18	N1	57.3(2)
C28	C27	C26	C31	0.7(3)	C21	C20	C18	C19	-65.3(2)
C28	C29	C30	C31	1.2(3)	C21	C20	C25	C24	0.7(3)
C26	C27	C28	C29	-0.1(3)	C21	C22	C23	C24	0.7(3)
C26	C1	C10	C9	2.1(3)	C39	C40	C41	C42	0.2(4)
C26	C1	C10	C5	-177.59(17)	C22	C23	C24	C25	0.2(3)
C26	C1	C2	C3	179.42(18)					

Table 7 Hydrogen Atom Coordinates (Å×10^{4}) and Isotropic Displacement Parameters (Å^{2}×10^{3}) for Li23_01.

Atom	*x*	*y*	*z*	U(eq)
H1	5754.31	6760.71	3644.35	30
H27	1001.77	5827.5	2209.73	24
H4	187.22	8643.2	91.83	30
H12	2804.13	6012.48	1519.34	22
H31	2331.37	7868.81	3098.87	23
H16	4108.87	8222.94	1942.4	25
H28	1353.98	5052.81	3394.78	23
H15	5129.83	7481.51	2733.33	25
H6	1561.82	8961.82	-548.57	31
H13	3802.49	5279.05	2330.41	23
H30	2694.41	7088.16	4279.28	22
H3	-632.89	8029.84	1134.89	33
H42	3831.09	5716.06	4364.89	34
H2A	66.5	7285.38	2208.26	30
H18	6358.84	5169.2	4091.34	30
H38	2325.9	5932.83	6416	29
H7	3063.19	8867.98	-597.51	32

Table 7 Hydrogen Atom Coordinates (Å×10^4) and Isotropic Displacement Parameters (Å^2×10^3) for Li23_01.

Atom	*x*	*y*	*z*	U(eq)
H8	3807.54	8062.18	403.59	29
H34	2959.44	3133.44	4503.81	30
H45A	-366.58	6079.36	4069.44	41
H45B	-1098.42	5491.06	4487.18	41
H45C	-110.3	5168.11	4451.36	41
H25	7411.8	4383.27	3364.7	32
H21	7430.59	6950.49	3085.37	33
H39	3561.73	6237.02	7204.93	39
H22	8601.41	6832.35	2174.78	41
H44A	-102.11	4960.66	5986.35	40
H44B	-1045.15	5358.41	6158.68	40
H44C	-204.2	5766.73	6591.5	40
H19A	7074.46	6790.99	4568.8	60
H19B	7329.2	5892.2	4978.46	60
H19C	6371.63	6274.54	5104.61	60
H35A	4537.1	3961.5	4013.85	55
H35B	4492.84	2980.09	4294.54	55
H35C	4273.61	3722.06	4946.78	55
H46A	-720.32	7180.76	5972.79	50
H46B	-1493.1	6716.84	5481.15	50
H46C	-788.16	7271.28	4985.07	50
H23	9202.81	5497.7	1879.02	41
H24	8608.97	4272.27	2477.43	40
H40	4933.91	6266.94	6585.24	45
H41	5058.89	5999.41	5161.53	47
H36A	2534.34	3245.41	3103.56	75
H36B	3360.12	2626.55	3185.32	75
H36C	3483.47	3587.58	2862.82	75
H2	1187(17)	5171(17)	5571(17)	37(7)

Experimental

Single crystals of $C_{46}H_{46}N_2O_4S$ **[Li23_01]** were **[]**. A suitable crystal was selected and **[Crystal was mounted on a 100 um MiTeGen cryoloop.]** on a **XtaLAB Synergy, Dualflex, HyPix** diffractometer. The crystal was kept at 100.3(6) K during data collection. Using Olex2 [1], the structure was solved with the SHELXT [2] structure solution program using Intrinsic Phasing and refined with the SHELXL [3] refinement package using Least Squares minimisation.

1. Dolomanov, O.V., Bourhis, L.J., Gildea, R.J, Howard, J.A.K. & Puschmann, H. (2009), J. Appl. Cryst. 42, 339-341.

2. Sheldrick, G.M. (2015). Acta Cryst. A71, 3-8.
3. Sheldrick, G.M. (2015). Acta Cryst. C71, 3-8.

Crystal structure determination of [Li23_01]

Crystal Data for $C_{46}H_{46}N_2O_4S$ (M =722.91 g/mol): orthorhombic, space group $P2_12_12_1$ (no. 19), a = 15.35810(10) Å, b = 15.62710(10) Å, c = 16.10440(10) Å, V = 3865.10(4) Å^3, Z = 4, T = 100.3(6) K, μ(Cu Kα) = 1.107 mm^{-1}, $Dcalc$ = 1.242 g/cm^3, 56496 reflections measured (7.884° ≤ 2Θ ≤ 154.96°), 8103 unique (R_{int} = 0.0370, R_{sigma} = 0.0205) which were used in all calculations. The final R_1 was 0.0282 (I > 2σ(I)) and wR_2 was 0.0715 (all data).

Refinement model description

Number of restraints - 0, number of constraints - unknown.
Details:
1. Fixed Uiso
 At 1.2 times of:
  All C(H) groups, All N(H) groups
 At 1.5 times of:
  All C(H,H,H) groups
2.a Ternary CH refined with riding coordinates:
 C18(H18), C34(H34)
2.b Aromatic/amide H refined with riding coordinates:
 N1(H1), C27(H27), C4(H4), C12(H12), C31(H31), C16(H16), C28(H28), C15(H15), C6(H6), C13(H13), C30(H30), C3(H3), C42(H42), C2(H2A), C38(H38), C7(H7), C8(H8), C25(H25), C21(H21), C39(H39), C22(H22), C23(H23), C24(H24), C40(H40),
 C41(H41)
2.c Idealised Me refined as rotating group:
 C45(H45A,H45B,H45C), C44(H44A,H44B,H44C), C19(H19A,H19B,H19C), C35(H35A,H35B,
 H35C), C46(H46A,H46B,H46C), C36(H36A,H36B,H36C)

This report has been created with Olex2, compiled on 2022.04.07 svn.rca3783a0 for OlexSys. Please let us know if there are any errors or if you would like to have additional features.

----Write up for **20**

General Data Collection

Data were collected on a Rigaku XtaLAB Synergy-*i* Kappa diffractometer equipped with a PhotonJet-*i* X-ray source operated at 50 W (50kV, 1 mA) to generate Cu Kα radiation (λ = 1.54178 Å) and a HyPix-6000HE HPC detector. Crystals were transferred from the vial and placed on a glass slide in type NVH immersion oil by

Cargille. A Zeiss Stemi 305 microscope was used to identify a suitable specimen for X-ray diffraction from a representative sample of the material. The crystal and a small amount of the oil were collected on a 100 micron MiTeGen cryoloop and transferred to the instrument where it was placed under a cold nitrogen stream (Oxford 700 series) maintained at 100K throughout the duration of the experiment. The sample was optically centered with the aid of a video camera to insure that no translations were observed as the crystal was rotated through all positions.

A unit cell collection was then carried out. After it was determined that the unit cell was not present in the CCDC database a data collection strategy was calculated by *CrysAlis^{Pro}*[1]. The crystal was measured for size, morphology, and color. These values are reported in the accompanying li22_02_tables file.

Refinement Details

After data collection, the unit cell was re-determined using a subset of the full data collection. Intensity data were corrected for Lorentz, polarization, and background effects using the *CrysAlis^{Pro}*[1]. A numerical absorption correction was applied based on a Gaussian integration over a multifaceted crystal and followed by a semi-empirical correction for adsorption applied using the program *SCALE3 ABSPACK*[2]. The programs *SHELXT*[3] was used for the initial structure solution and *SHELXL*[4] was used for refinement of the structure. Both of these programs were utilized within the OLEX2 software[5]. Within this structure, the isopropyl group containing C44 < C46 were positionally disordered. Each atom was split into parts A and B and allowed to freely refine to a stable occupancy of 60% and 40%, respectively. To help maintain reasonable

ADPs for the disordered sites, SIMU and RIGU restraints were applied. Hydrogen atoms bound to the carbon and nitrogen atoms were located in the difference Fourier map where possible and were geometrically constrained using the appropriate AFIX commands.

References:
1. CrysAlis[Pro] (2018) Oxford Diffraction Ltd.
2. SCALE3 ABSPACK (2005) Oxford Diffraction Ltd.
3. Sheldrick, G. M. (2015) *Acta Crystallogr.*, C**71**, 3-8.
4. Sheldrick, G. M. (2015) *Acta Crystallogr.*, A**71**, 3-8.
5. Dolomanov, O. V.; Bourhis, . L. J.; Gildea, R. J.; Howard, J. A. K.; Puschmann. H. (2009) *J. Appl. Cryst.* **42**, 339-341.

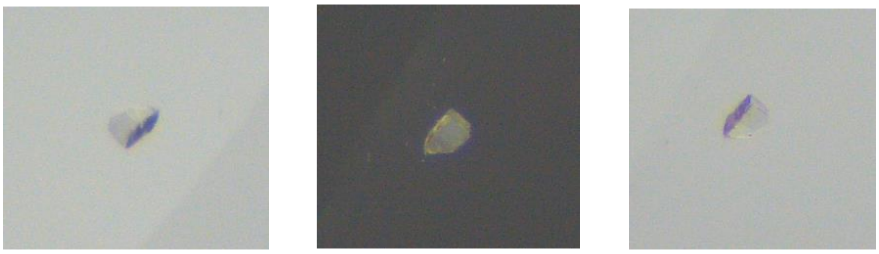

Crystal used for single crystal X-ray diffraction experiment.

Li22_02_auto

Table 1 Crystal data and structure refinement for Li22_02_auto.

Identification code	Li22_02_auto
Empirical formula	$C_{46}H_{46}N_2O_4S$
Formula weight	722.91
Temperature/K	100.01(10)
Crystal system	monoclinic
Space group	$P2_1$
a/Å	9.51160(10)
b/Å	18.03100(10)
c/Å	12.31190(10)
α/°	90
β/°	110.1260(10)
γ/°	90
Volume/Å^3	1982.60(3)
Z	2
ρ_{calc}g/cm^3	1.211
μ/mm^{-1}	1.079

F(000)	768.0
Crystal size/mm^3	0.14 × 0.111 × 0.089
Radiation	Cu Kα (λ = 1.54184)
2Θ range for data collection/°	7.648 to 154.548
Index ranges	-12 ≤ h ≤ 12, -22 ≤ k ≤ 22, -15 ≤ l ≤ 14
Reflections collected	45633
Independent reflections	8203 [R_{int} = 0.0405, R_{sigma} = 0.0248]
Data/restraints/parameters	8203/85/522
Goodness-of-fit on F^2	1.038
Final R indexes [I>=2σ (I)]	R_1 = 0.0284, wR_2 = 0.0709
Final R indexes [all data]	R_1 = 0.0294, wR_2 = 0.0716
Largest diff. peak/hole / e Å^{-3}	0.14/-0.28
Flack parameter	0.011(7)

Table 2 Fractional Atomic Coordinates (×10^4) and Equivalent Isotropic Displacement Parameters (Å^2×10^3) for Li22_02_auto. U_{eq} is defined as 1/3 of the trace of the orthogonalised U_{IJ} tensor.

Atom	*x*	*y*	*z*	U(eq)
S1	9573.2(5)	5964.6(3)	7092.3(4)	18.75(10)
O1	5905.5(15)	6381.3(8)	8556.6(12)	22.2(3)
O2	10756.2(15)	5710.6(8)	8182.7(12)	24.6(3)
O3	6708.4(18)	4782.3(8)	8191.9(13)	28.6(3)
O4	7046(2)	3743.0(8)	7323.3(15)	33.1(4)
N1	4031.7(18)	5710.6(9)	8832.8(13)	19.4(3)
N2	7893.9(17)	5646.3(9)	6975.4(14)	19.6(3)
C17	4635(2)	6113.9(10)	8185.8(15)	17.7(4)
C43	7009(2)	4474.5(11)	7437.6(16)	19.9(4)
C14	3656(2)	6205.3(10)	6948.8(15)	17.6(4)
C13	3699(2)	6856.6(11)	6354.2(16)	19.4(4)
C29	5878(2)	4994.2(11)	5446.5(16)	20.2(4)
C26	3178(2)	5175.1(12)	3572.1(16)	22.5(4)
C12	2786(2)	6938.4(11)	5207.8(16)	21.6(4)
C20	4622(2)	4692.6(11)	10240.0(16)	22.4(4)
C15	2694(2)	5635.1(11)	6379.5(16)	19.4(4)
C27	4232(2)	5742.1(12)	3901.2(16)	22.5(4)
C1	1718(2)	5272.6(12)	2611.6(16)	23.7(4)
C32	7362(2)	4905.2(10)	6472.3(17)	19.5(4)
C37	8538(2)	4499.7(11)	6092.4(18)	22.5(4)
C11	1841(2)	6358.6(11)	4625.9(16)	21.6(4)

Table 2 Fractional Atomic Coordinates (×10⁴) and Equivalent Isotropic Displacement Parameters (Å²×10³) for Li22_02_auto. U_{eq} is defined as 1/3 of the trace of the orthogonalised U_{IJ} tensor.

Atom	*x*	*y*	*z*	U(eq)
C16	1798(2)	5713.7(11)	5231.7(16)	20.8(4)
C10	721(2)	5889.5(12)	2515.2(15)	23.1(4)
C28	5558(2)	5654.6(12)	4832.8(16)	22.0(4)
C42	8346(3)	4390.9(12)	4932(2)	28.8(4)
C5	-512(2)	5964.0(14)	1442.5(16)	25.7(4)
C22	3019(3)	3625.8(13)	10050(2)	30.9(5)
C21	3220(2)	4359.7(12)	9803.0(18)	26.2(4)
C9	829(2)	6432.8(12)	3399.0(17)	24.1(4)
C31	3540(2)	4501.9(12)	4152.8(18)	24.9(4)
C4	-782(2)	5418.0(14)	574.8(18)	29.6(5)
C36	7947(3)	7260.0(13)	6410(2)	34.8(5)
C30	4865(2)	4406.8(12)	5074.0(18)	24.3(4)
C7	-1295(3)	7107.6(13)	2080.9(19)	34.1(5)
C6	-1484(2)	6583.3(13)	1253.6(18)	30.3(5)
C41	9439(3)	4028.2(13)	4616(2)	35.9(5)
C18	4862(2)	5500.8(11)	10027.6(16)	22.4(4)
C38	9843(2)	4238.9(11)	6932(2)	27.1(4)
C8	-144(3)	7025.1(13)	3158.3(19)	32.2(5)
C33	9282(2)	6952.5(11)	7388.2(18)	23.1(4)
C34	9102(3)	7028.9(12)	8564(2)	29.4(5)
C40	10739(3)	3780.4(13)	5461(3)	39.4(6)
C39	10942(3)	3891.6(12)	6613(2)	33.9(5)
C2	1377(3)	4747.5(14)	1742.3(19)	31.6(5)
C3	114(3)	4811.8(15)	725.6(19)	34.7(5)
C35	10736(3)	7326.0(12)	7406(2)	30.8(5)
C19	4431(3)	6002.1(14)	10861.7(18)	35.1(5)
C24	5600(3)	3531.6(17)	11171(3)	56.7(9)
C25	5809(3)	4267.3(15)	10922(3)	45.6(7)
C23	4204(3)	3207.4(14)	10741(2)	40.1(6)
C44A	6811(7)	3311(3)	8287(6)	23.4(12)
C46A	7741(8)	2616(4)	8470(7)	41.7(16)
C45A	5151(5)	3184(3)	7882(4)	41.8(15)
C44B	6313(12)	3284(6)	7983(10)	33(2)
C45B	5137(8)	2772(5)	7218(7)	46(2)
C46B	7664(13)	2884(8)	8739(13)	64(4)

Table 3 Anisotropic Displacement Parameters ($Å^2×10^3$) for Li22_02_auto. The Anisotropic displacement factor exponent takes the form: $-2\pi^2[h^2a*^2U_{11}+2hka*b*U_{12}+\cdots]$.

Atom	U_{11}	U_{22}	U_{33}	U_{23}	U_{13}	U_{12}
S1	16.11(19)	18.2(2)	21.4(2)	0.60(17)	5.84(15)	0.36(17)
O1	18.8(6)	22.6(7)	21.5(6)	1.0(5)	2.3(5)	-2.7(5)
O2	17.4(6)	25.0(7)	28.0(7)	3.0(6)	3.5(5)	1.1(5)
O3	37.5(8)	25.5(8)	25.1(7)	-3.6(6)	13.8(6)	-1.9(6)
O4	52.5(10)	17.1(7)	42.1(9)	2.4(6)	32.1(8)	2.3(7)
N1	14.8(7)	26.3(8)	15.2(7)	2.7(6)	2.9(6)	-0.1(6)
N2	16.9(7)	18.5(8)	22.7(8)	-3.9(6)	5.9(6)	-0.7(6)
C17	16.2(8)	16.7(9)	19.3(8)	-1.8(7)	4.9(6)	2.6(7)
C43	15.9(8)	19.6(9)	21.8(9)	-0.5(7)	3.3(7)	1.4(7)
C14	15.8(8)	20.6(9)	17.2(8)	-1.1(7)	6.5(7)	1.1(7)
C13	20.1(9)	18.3(9)	19.7(9)	-2.5(7)	6.6(7)	-2.6(7)
C29	18.5(9)	22.5(10)	19.5(9)	-2.3(7)	6.5(7)	-0.2(7)
C26	21.6(9)	28.5(10)	17.5(9)	-4.8(8)	6.8(7)	-2.3(8)
C12	26.6(9)	19.5(9)	18.4(9)	1.0(7)	7.4(7)	-1.1(8)
C20	23.0(9)	24.4(10)	19.4(9)	1.7(7)	6.6(8)	2.3(8)
C15	20.0(9)	20.5(9)	18.5(8)	0.7(7)	7.8(7)	-1.2(7)
C27	23.0(9)	26.5(10)	17.4(8)	1.4(7)	6.4(7)	-1.9(8)
C1	22.1(9)	30.5(11)	17.6(9)	-2.7(8)	5.5(7)	-4.8(8)
C32	18.6(9)	16.6(9)	22.3(9)	-1.9(7)	5.7(7)	-0.4(7)
C37	22.2(9)	16.9(9)	30.6(10)	-4.0(8)	11.8(8)	-2.6(7)
C11	21.8(9)	24.4(10)	17.3(9)	-2.3(7)	5.1(7)	-0.9(8)
C16	19.0(8)	22.3(9)	19.9(9)	-4.2(7)	5.1(7)	-4.0(7)
C10	21.6(8)	28.0(10)	17.9(8)	0.9(8)	4.5(7)	-6.1(8)
C28	21.0(9)	25.1(9)	19.3(9)	-1.8(7)	6.2(7)	-4.4(8)
C42	31.3(11)	25.4(11)	32.9(11)	-7.8(9)	15.3(9)	-6.1(9)
C5	22.7(9)	34.3(10)	17.3(8)	1.4(9)	3.5(7)	-5.2(9)
C22	34.7(11)	26.5(11)	31.3(11)	-0.8(9)	11.0(9)	-4.4(9)
C21	25.9(10)	25.2(10)	24.9(10)	2.4(8)	5.4(8)	1.6(8)
C9	25.5(10)	24.1(10)	19.4(9)	0.7(8)	3.5(8)	-2.5(8)
C31	23.2(9)	23.6(10)	26.4(10)	-6.8(8)	6.7(8)	-5.5(8)
C4	22.8(10)	43.4(13)	18.2(9)	-3.6(9)	1.5(8)	-6.9(9)
C36	32.9(12)	20.7(11)	43.7(13)	3.6(10)	4.3(10)	3.7(9)
C30	24.9(10)	20.7(10)	26.1(10)	-1.9(8)	7.4(8)	-0.8(8)
C7	34.5(12)	29.6(12)	27.3(11)	3.6(9)	-3.3(9)	4.3(9)
C6	27.2(10)	35.1(12)	21.1(10)	5.6(8)	-1.2(8)	-3.8(9)
C41	47.3(14)	26.9(11)	45.1(14)	-11.6(10)	30.4(11)	-7.7(10)
C18	21.7(9)	25.7(10)	16.1(9)	3.3(7)	1.5(7)	0.6(8)

Table 3 Anisotropic Displacement Parameters ($Å^2×10^3$) for Li22_02_auto. The Anisotropic displacement factor exponent takes the form: $-2\pi^2[h^2a*^2U_{11}+2hka*b*U_{12}+\cdots]$.

Atom	U_{11}	U_{22}	U_{33}	U_{23}	U_{13}	U_{12}
C38	25.5(10)	19.0(10)	38.7(11)	0.8(8)	13.4(9)	1.5(8)
C8	38.0(12)	26.9(11)	24.3(10)	-1.9(8)	1.4(9)	1.6(9)
C33	21.4(9)	18.0(9)	30.2(10)	-1.4(8)	9.3(8)	-1.0(7)
C34	30.5(11)	26.5(11)	35.4(11)	-10.1(9)	16.8(9)	-7.3(9)
C40	40.4(13)	19.7(11)	72.9(18)	-6.3(11)	38.3(13)	-1.5(9)
C39	27.5(11)	20.7(11)	57.6(15)	3.6(10)	19.9(10)	3.9(9)
C2	28.0(11)	39.4(12)	25.1(10)	-9.5(9)	6.2(9)	-0.7(9)
C3	30.6(11)	47.6(14)	21.7(10)	-14.5(10)	3.6(9)	-5.4(10)
C35	29.8(11)	22.1(11)	44.7(13)	-1.2(9)	18.2(10)	-5.2(8)
C19	55.3(14)	27.7(11)	23.1(10)	-2.6(9)	14.6(9)	-0.8(12)
C24	36.4(14)	41.8(16)	83(2)	31.0(15)	9.2(14)	13.0(12)
C25	24.6(11)	41.5(15)	59.5(17)	22.8(12)	0.1(11)	2.3(10)
C23	44.7(14)	23.9(12)	54.3(15)	10.8(11)	20.2(12)	6.3(10)
C44A	23(2)	21(2)	29(3)	8.5(17)	13.1(18)	4.7(17)
C46A	41(3)	25(3)	67(4)	20(2)	29(3)	15(2)
C45A	24.2(19)	49(3)	52(3)	17(2)	13.5(18)	-0.4(17)
C44B	40(5)	26(3)	37(5)	11(3)	17(3)	4(3)
C45B	44(3)	46(4)	44(4)	14(3)	13(3)	-11(3)
C46B	39(4)	58(8)	81(8)	45(6)	4(4)	-7(4)

Table 4 Bond Lengths for Li22_02_auto.

Atom	Atom	Length/Å	Atom	Atom	Length/Å
S1	O2	1.4962(14)	C37	C42	1.390(3)
S1	N2	1.6563(17)	C37	C38	1.395(3)
S1	C33	1.858(2)	C11	C16	1.390(3)
O1	C17	1.234(2)	C11	C9	1.492(3)
O3	C43	1.198(2)	C10	C5	1.439(2)
O4	C43	1.328(2)	C10	C9	1.441(3)
O4	C44A	1.499(7)	C42	C41	1.393(3)
O4	C44B	1.490(11)	C5	C4	1.410(3)
N1	C17	1.344(2)	C5	C6	1.417(3)
N1	C18	1.460(2)	C22	C21	1.386(3)
N2	C32	1.486(2)	C22	C23	1.380(3)
C17	C14	1.496(2)	C9	C8	1.377(3)
C43	C32	1.550(3)	C31	C30	1.386(3)
C14	C13	1.392(3)	C4	C3	1.359(4)
C14	C15	1.394(3)	C36	C33	1.523(3)

Table 4 Bond Lengths for Li22_02_auto.

Atom	Atom	Length/Å	Atom	Atom	Length/Å
C13	C12	1.387(3)	C7	C6	1.355(3)
C29	C32	1.545(3)	C7	C8	1.407(3)
C29	C28	1.387(3)	C41	C40	1.387(4)
C29	C30	1.399(3)	C18	C19	1.526(3)
C26	C27	1.391(3)	C38	C39	1.386(3)
C26	C1	1.492(3)	C33	C34	1.521(3)
C26	C31	1.391(3)	C33	C35	1.532(3)
C12	C11	1.404(3)	C40	C39	1.379(4)
C20	C21	1.391(3)	C2	C3	1.410(3)
C20	C18	1.512(3)	C24	C25	1.391(4)
C20	C25	1.384(3)	C24	C23	1.379(4)
C15	C16	1.384(3)	C44A	C46A	1.505(7)
C27	C28	1.392(3)	C44A	C45A	1.501(6)
C1	C10	1.440(3)	C44B	C45B	1.505(13)
C1	C2	1.381(3)	C44B	C46B	1.488(13)
C32	C37	1.537(3)			

Table 5 Bond Angles for Li22_02_auto.

Atom	Atom	Atom	Angle/°	Atom	Atom	Atom	Angle/°
O2	S1	N2	112.14(8)	C15	C16	C11	120.81(18)
O2	S1	C33	103.55(9)	C1	C10	C9	126.08(17)
N2	S1	C33	98.31(9)	C5	C10	C1	116.96(18)
C43	O4	C44A	114.6(3)	C5	C10	C9	116.96(19)
C43	O4	C44B	117.3(5)	C29	C28	C27	120.88(18)
C17	N1	C18	122.95(16)	C37	C42	C41	120.4(2)
C32	N2	S1	121.82(13)	C4	C5	C10	120.6(2)
O1	C17	N1	123.75(17)	C4	C5	C6	119.17(18)
O1	C17	C14	121.39(17)	C6	C5	C10	120.2(2)
N1	C17	C14	114.84(16)	C23	C22	C21	120.8(2)
O3	C43	O4	124.36(19)	C22	C21	C20	120.9(2)
O3	C43	C32	122.33(18)	C10	C9	C11	123.85(18)
O4	C43	C32	113.29(16)	C8	C9	C11	116.06(19)
C13	C14	C17	120.52(16)	C8	C9	C10	119.77(18)
C13	C14	C15	119.38(17)	C30	C31	C26	121.54(19)
C15	C14	C17	120.10(16)	C3	C4	C5	121.06(19)
C12	C13	C14	120.24(17)	C31	C30	C29	120.32(19)
C28	C29	C32	120.15(17)	C6	C7	C8	119.6(2)
C28	C29	C30	118.29(17)	C7	C6	C5	121.05(19)

Table 5 Bond Angles for Li22_02_auto.

Atom	Atom	Atom	Angle/°	Atom	Atom	Atom	Angle/°
C30	C29	C32	121.48(18)	C40	C41	C42	120.0(2)
C27	C26	C1	121.66(19)	N1	C18	C20	111.24(16)
C31	C26	C27	117.76(18)	N1	C18	C19	110.30(17)
C31	C26	C1	120.56(18)	C20	C18	C19	111.27(18)
C13	C12	C11	120.44(18)	C39	C38	C37	120.5(2)
C21	C20	C18	122.09(18)	C9	C8	C7	122.1(2)
C25	C20	C21	118.0(2)	C36	C33	S1	109.51(15)
C25	C20	C18	119.9(2)	C36	C33	C35	110.74(18)
C16	C15	C14	120.33(18)	C34	C33	S1	110.28(15)
C26	C27	C28	121.00(19)	C34	C33	C36	112.59(19)
C10	C1	C26	124.27(18)	C34	C33	C35	110.06(18)
C2	C1	C26	116.10(19)	C35	C33	S1	103.26(14)
C2	C1	C10	119.53(18)	C39	C40	C41	119.9(2)
N2	C32	C43	105.26(15)	C40	C39	C38	120.3(2)
N2	C32	C29	109.36(15)	C1	C2	C3	122.2(2)
N2	C32	C37	112.06(15)	C4	C3	C2	119.3(2)
C29	C32	C43	107.49(15)	C23	C24	C25	120.7(2)
C37	C32	C43	111.55(16)	C20	C25	C24	120.9(2)
C37	C32	C29	110.87(16)	C24	C23	C22	118.6(2)
C42	C37	C32	121.82(19)	O4	C44A	C46A	108.1(5)
C42	C37	C38	118.8(2)	O4	C44A	C45A	104.1(4)
C38	C37	C32	119.33(18)	C45A	C44A	C46A	114.8(6)
C12	C11	C9	121.78(18)	O4	C44B	C45B	112.7(8)
C16	C11	C12	118.77(17)	C46B	C44B	O4	98.3(8)
C16	C11	C9	119.38(17)	C46B	C44B	C45B	113.0(10)

Table 6 Torsion Angles for Li22_02_auto.

A	B	C	D	Angle/°	A	B	C	D	Angle/°
S1	N2	C32	C43	121.25(15)	C32	C29	C30	C31	179.85(18)
S1	N2	C32	C29	-123.53(15)	C32	C37	C42	C41	179.31(19)
S1	N2	C32	C37	-0.2(2)	C32	C37	C38	C39	-178.01(19)
O1	C17	C14	C13	-35.0(3)	C37	C42	C41	C40	-0.7(3)
O1	C17	C14	C15	144.73(19)	C37	C38	C39	C40	-1.9(3)
O2	S1	N2	C32	-85.85(16)	C11	C9	C8	C7	-171.2(2)
O2	S1	C33	C36	-172.33(15)	C16	C11	C9	C10	-52.1(3)
O2	S1	C33	C34	-47.90(16)	C16	C11	C9	C8	121.3(2)
O2	S1	C33	C35	69.67(15)	C10	C1	C2	C3	3.3(3)
O3	C43	C32	N2	24.3(2)	C10	C5	C4	C3	-0.3(3)

Table 6 Torsion Angles for Li22_02_auto.

A	B	C	D	Angle/°	A	B	C	D	Angle/°
O3	C43	C32	C29	-92.2(2)	C10	C5	C6	C7	-1.3(3)
O3	C43	C32	C37	146.09(18)	C10	C9	C8	C7	2.5(4)
O4	C43	C32	N2	-156.91(16)	C28	C29	C32	N2	26.2(2)
O4	C43	C32	C29	86.59(19)	C28	C29	C32	C43	140.00(18)
O4	C43	C32	C37	-35.1(2)	C28	C29	C32	C37	-97.8(2)
N1	C17	C14	C13	146.33(18)	C28	C29	C30	C31	-3.4(3)
N1	C17	C14	C15	-33.9(2)	C42	C37	C38	C39	1.4(3)
N2	S1	C33	C36	-57.05(16)	C42	C41	C40	C39	0.2(3)
N2	S1	C33	C34	67.38(15)	C5	C10	C9	C11	167.64(19)
N2	S1	C33	C35	-175.05(14)	C5	C10	C9	C8	-5.6(3)
N2	C32	C37	C42	-109.5(2)	C5	C4	C3	C2	-3.2(4)
N2	C32	C37	C38	69.9(2)	C21	C20	C18	N1	-42.2(3)
C17	N1	C18	C20	-134.46(19)	C21	C20	C18	C19	81.2(2)
C17	N1	C18	C19	101.6(2)	C21	C20	C25	C24	-0.6(4)
C17	C14	C13	C12	-179.87(18)	C21	C22	C23	C24	-0.8(4)
C17	C14	C15	C16	-179.31(17)	C9	C11	C16	C15	-178.32(18)
C43	O4	C44A	C46A	-146.0(5)	C9	C10	C5	C4	-173.99(19)
C43	O4	C44A	C45A	91.5(4)	C9	C10	C5	C6	5.0(3)
C43	O4	C44B	C45B	123.9(7)	C31	C26	C27	C28	-4.4(3)
C43	O4	C44B	C46B	-116.9(9)	C31	C26	C1	C10	131.3(2)
C43	C32	C37	C42	132.8(2)	C31	C26	C1	C2	-52.3(3)
C43	C32	C37	C38	-47.9(2)	C4	C5	C6	C7	177.7(2)
C14	C13	C12	C11	-1.8(3)	C30	C29	C32	N2	-157.12(18)
C14	C15	C16	C11	0.1(3)	C30	C29	C32	C43	-43.3(2)
C13	C14	C15	C16	0.5(3)	C30	C29	C32	C37	78.8(2)
C13	C12	C11	C16	2.4(3)	C30	C29	C28	C27	2.8(3)
C13	C12	C11	C9	179.06(19)	C6	C5	C4	C3	-179.3(2)
C29	C32	C37	C42	13.0(3)	C6	C7	C8	C9	1.4(4)
C29	C32	C37	C38	-167.62(18)	C41	C40	C39	C38	1.1(3)
C26	C27	C28	C29	1.2(3)	C18	N1	C17	O1	-4.6(3)
C26	C1	C10	C5	169.80(18)	C18	N1	C17	C14	174.03(17)
C26	C1	C10	C9	-11.2(3)	C18	C20	C21	C22	-177.3(2)
C26	C1	C2	C3	-173.3(2)	C18	C20	C25	C24	177.2(3)
C26	C31	C30	C29	0.2(3)	C38	C37	C42	C41	-0.1(3)
C12	C11	C16	C15	-1.5(3)	C8	C7	C6	C5	-2.0(4)
C12	C11	C9	C10	131.2(2)	C33	S1	N2	C32	165.78(15)
C12	C11	C9	C8	-55.4(3)	C2	C1	C10	C5	-6.5(3)
C15	C14	C13	C12	0.4(3)	C2	C1	C10	C9	172.5(2)

Table 6 Torsion Angles for Li22_02_auto.

A	B	C	D	Angle/°	A	B	C	D	Angle/°
C27	C26	C1	C10	-50.1(3)	C25	C20	C21	C22	0.4(3)
C27	C26	C1	C2	126.3(2)	C25	C20	C18	N1	140.1(2)
C27	C26	C31	C30	3.7(3)	C25	C20	C18	C19	-96.5(3)
C1	C26	C27	C28	177.03(18)	C25	C24	C23	C22	0.6(5)
C1	C26	C31	C30	-177.69(19)	C23	C22	C21	C20	0.3(4)
C1	C10	C5	C4	5.1(3)	C23	C24	C25	C20	0.1(5)
C1	C10	C5	C6	-175.85(19)	C44A	O4	C43	O3	-5.2(4)
C1	C10	C9	C11	-11.4(3)	C44A	O4	C43	C32	176.1(3)
C1	C10	C9	C8	175.4(2)	C44B	O4	C43	O3	15.8(6)
C1	C2	C3	C4	1.7(4)	C44B	O4	C43	C32	-163.0(5)
C32	C29	C28	C27	179.53(18)					

Table 7 Hydrogen Atom Coordinates (Å×10^4) and Isotropic Displacement Parameters (Å^2×10^3) for Li22_02_auto.

Atom	*x*	*y*	*z*	U(eq)
H13	4355.93	7246.4	6734.79	23
H12	2800.78	7389.83	4813.88	26
H15	2653.26	5190.51	6781.68	23
H27	4043.09	6195.88	3483.67	27
H16	1144.89	5322.02	4853.14	25
H28	6253.6	6052.64	5051.31	26
H42	7462.26	4565.03	4351.26	35
H22	2053.26	3408.03	9739.76	37
H21	2388.86	4639.18	9328.38	31
H31	2862.42	4097.6	3912.48	30
H4	-1603.8	5474.79	-126.49	35
H36A	8059.84	7145.4	5665.69	52
H36B	7898.85	7798.85	6494.73	52
H36C	7023.73	7033.73	6438.09	52
H30	5086.33	3940.56	5454.2	29
H7	-1934.97	7527.99	1934.47	41
H6	-2280.72	6631.91	535.63	36
H41	9295.14	3950.54	3821.55	43
H18	5952.61	5574.63	10167.14	27
H38	9979.16	4299.86	7727.95	33
H8	-34.79	7389.88	3738.36	39
H34A	8139.31	6816.19	8530.25	44
H34B	9132.69	7554.72	8772.31	44

Table 7 Hydrogen Atom Coordinates (Å×10[4]) and Isotropic Displacement Parameters (Å[2]×10[3]) for Li22_02_auto.

Atom	*x*	*y*	*z*	U(eq)
H34C	9917.12	6764.87	9147.23	44
H40	11486.92	3534.67	5245.54	47
H39	11839.74	3729.51	7191.16	41
H2A	2014.12	4329.06	1832.38	38
H3	-108.28	4435.11	151.68	42
H35A	11582.74	7112.43	8029.5	46
H35B	10679.53	7859.51	7537.94	46
H35C	10876.21	7245.13	6663.07	46
H19A	3370.13	5930.9	10753.24	53
H19B	4605.5	6521.06	10708.15	53
H19C	5041.58	5876.15	11659.19	53
H24	6429.24	3249.16	11641.8	68
H25	6779.37	4480.74	11224.29	55
H23	4060.8	2706.37	10918.09	48
H44A	7128.47	3614.26	9010.79	28
H46A	7589.39	2320.67	9088.48	63
H46B	8801.66	2749.87	8688.81	63
H46C	7439.26	2326.63	7753.34	63
H45A	4851.19	2875.62	7183.51	63
H45B	4630.15	3661.4	7707.45	63
H45C	4888.13	2930.98	8490.35	63
H44B	5885.06	3605.22	8454.25	40
H45D	4296	3065.49	6716.46	68
H45E	4780.94	2440.24	7698.2	68
H45F	5567.38	2477.88	6740.61	68
H46D	8039.13	2556.02	8264.85	96
H46E	7401	2588.13	9308.92	96
H46F	8441.18	3243.71	9139.96	96
H2	7530(30)	5753(16)	7540(20)	38(7)
H1	3090(30)	5643(14)	8580(20)	26(6)

Table 8 Atomic Occupancy for Li22_02_auto.

Atom	*Occupancy*	Atom	*Occupancy*	Atom	*Occupancy*
C44A	0.604(9)	H44A	0.604(9)	C46A	0.604(9)
H46A	0.604(9)	H46B	0.604(9)	H46C	0.604(9)
C45A	0.604(9)	H45A	0.604(9)	H45B	0.604(9)
H45C	0.604(9)	C44B	0.396(9)	H44B	0.396(9)

Table 8 Atomic Occupancy for Li22_02_auto.

Atom *Occupancy*	Atom *Occupancy*	Atom *Occupancy*
C45B 0.396(9)	H45D 0.396(9)	H45E 0.396(9)
H45F 0.396(9)	C46B 0.396(9)	H46D 0.396(9)
H46E 0.396(9)	H46F 0.396(9)	

Experimental

Single crystals of $C_{46}H_{46}N_2O_4S$ **[Li22_02_auto]** were **[]**. A suitable crystal was selected and **[Crystal was mounted on a 50 um MiTeGen cryoloop.]** on a **XtaLAB Synergy, Dualflex, HyPix** diffractometer. The crystal was kept at 100.01(10) K during data collection. Using Olex2 [1], the structure was solved with the SHELXT [2] structure solution program using Intrinsic Phasing and refined with the SHELXL [3] refinement package using Least Squares minimisation.

1. Dolomanov, O.V., Bourhis, L.J., Gildea, R.J, Howard, J.A.K. & Puschmann, H. (2009), J. Appl. Cryst. 42, 339-341.
2. Sheldrick, G.M. (2015). Acta Cryst. A71, 3-8.
3. Sheldrick, G.M. (2015). Acta Cryst. C71, 3-8.

Crystal structure determination of [Li22_02_auto]

Crystal Data for $C_{46}H_{46}N_2O_4S$ (M =722.91 g/mol): monoclinic, space group $P2_1$ (no. 4), a = 9.51160(10) Å, b = 18.03100(10) Å, c = 12.31190(10) Å, β = 110.1260(10)°, V = 1982.60(3) Å^3, Z = 2, T = 100.01(10) K, μ(Cu Kα) = 1.079 mm^{-1}, $Dcalc$ = 1.211 g/cm^3, 45633 reflections measured (7.648° ⩽ 2Θ ⩽ 154.548°), 8203 unique (R_{int} = 0.0405, R_{sigma} = 0.0248) which were used in all calculations. The final R_1 was 0.0284 (I > 2σ(I)) and wR_2 was 0.0716 (all data).

Refinement model description

Number of restraints - 85, number of constraints - unknown.

Details:

1. Fixed Uiso

At 1.2 times of:

All C(H) groups

At 1.5 times of:

All C(H,H,H) groups

2. Uiso/Uaniso restraints and constraints

C44A ≈ C46A ≈ C45A ≈ C44B ≈ C45B ≈ C46B: within 2A with sigma of 0.02 and sigma for terminal atoms of 0.04 within 2A

3. Rigid body (RIGU) restrains

C44A, C46A, C45A, C44B, C45B, C46B

with sigma for 1-2 distances of 0.001 and sigma for 1-3 distances of 0.001

4. Others

Sof(C44B)=Sof(H44B)=Sof(C45B)=Sof(H45d)=Sof(H45e)=Sof(H45f)=Sof(C46B)=
Sof(H46d)=Sof(H46e)=Sof(H46f)=1-FVAR(1)

Sof(C44A)=Sof(H44A)=Sof(C46A)=Sof(H46a)=Sof(H46b)=Sof(H46c)=Sof(C45A)=
Sof(H45a)=Sof(H45b)=Sof(H45c)=FVAR(1)

5.a Ternary CH refined with riding coordinates:

C18(H18), C44A(H44A), C44B(H44B)

5.b Aromatic/amide H refined with riding coordinates:
C13(H13), C12(H12), C15(H15), C27(H27), C16(H16), C28(H28), C42(H42), C22(H22), C21(H21), C31(H31), C4(H4), C30(H30), C7(H7), C6(H6), C41(H41), C38(H38), C8(H8), C40(H40), C39(H39), C2(H2a), C3(H3), C24(H24), C25(H25), C23(H23)

5.c Idealised Me refined as rotating group:
C36(H36a,H36b,H36c), C34(H34a,H34b,H34c), C35(H35a,H35b,H35c), C19(H19a,H19b, H19c), C46A(H46a,H46b,H46c), C45A(H45a,H45b,H45c), C45B(H45d,H45e,H45f), C46B(H46d,H46e,H46f)

This report has been created with Olex2, compiled on 2022.04.07 svn.rca3783a0 for OlexSys. Please let us know if there are any errors or if you would like to have additional features.
